# Blood DNA methylomic signatures associated with CSF biomarkers of Alzheimer's disease in the EMIF‐AD study

**DOI:** 10.1002/alz.14098

**Published:** 2024-08-28

**Authors:** Rebecca G. Smith, Ehsan Pishva, Morteza Kouhsar, Jennifer Imm, Valerija Dobricic, Peter Johannsen, Michael Wittig, Andre Franke, Rik Vandenberghe, Jolien Schaeverbeke, Yvonne Freund‐Levi, Lutz Frölich, Philip Scheltens, Charlotte E. Teunissen, Giovanni Frisoni, Olivier Blin, Jill C. Richardson, Régis Bordet, Sebastiaan Engelborghs, Ellen de Roeck, Pablo Martinez‐Lage, Miren Altuna, Mikel Tainta, Alberto Lleó, Isabel Sala, Julius Popp, Gwendoline Peyratout, Laura Winchester, Alejo Nevado‐Holgado, Frans Verhey, Magda Tsolaki, Ulf Andreasson, Kaj Blennow, Henrik Zetterberg, Johannes Streffer, Stephanie J. B. Vos, Simon Lovestone, Pieter Jelle Visser, Lars Bertram, Katie Lunnon

**Affiliations:** ^1^ Department of Clinical and Biomedical Sciences Faculty of Health and Life Sciences University of Exeter Exeter Devon UK; ^2^ Department of Psychiatry and Neuropsychology, School for Mental Health and Neuroscience (MHeNs), Faculty of Health, Medicine and Life Sciences (FHML) Maastricht University Maastricht The Netherlands; ^3^ Lübeck Interdisciplinary Platform for Genome Analytics (LIGA) University of Lübeck Lübeck Germany; ^4^ Danish Dementia Research Centre, Rigshospitalet Copenhagen Denmark; ^5^ Institute of Clinical Molecular Biology Christian‐Albrechts‐University of Kiel Kiel Germany; ^6^ Laboratory for Cognitive Neurology KU Leuven, Leuven Brain Institute Leuven Belgium; ^7^ Department of Clinical Science and Education Södersjukhuset, Karolinska Institutet Stockholm Sweden; ^8^ School of Medical Sciences Örebro University Örebro Sweden; ^9^ Department of Geriatrics Södertälje Hospital Södertälje Sweden; ^10^ Department of Geriatric Psychiatry Central Institut of Mental Health Medical Faculty Mannheim/Heidelberg University Mannheim Germany; ^11^ Alzheimer Center Amsterdam, Department of Neurology, Amsterdam Neuroscience Vrije Universiteit Amsterdam, Amsterdam UMC Amsterdam The Netherlands; ^12^ Neurochemistry Laboratory Department of Laboratory Medicine, Amsterdam Neuroscience Vrije Universiteit Amsterdam, Amsterdam UMC Amsterdam The Netherlands; ^13^ Memory center Geneva University and University Hospitals; on behalf of the AMYPAD consortium Geneva Switzerland; ^14^ Aix‐Marseille University‐CNRS Marseille France; ^15^ Neuroscience Therapeutic Area, GlaxoSmithKline R&D Stevenage Hertfordshire UK; ^16^ Université de Lille Lille Cedex France; ^17^ Department of Biomedical Sciences University of Antwerp Antwerp Belgium; ^18^ Neuroprotection & Neuromodulation (NEUR) Research Group, Center for Neurosciences (C4N) Vrije Universiteit Brussel (VUB), Jette Brussels Belgium; ^19^ Center for Research and Advanced Therapies Fundación CITA‐Alzhéimer Fundazioa San Sebastian Gipuzkoa Spain; ^20^ Servicio de Neurología, Centre of Biomedical Investigation Network for Neurodegenerative Diseases (CIBERNED) Hospital Sant Pau Barcelona Spain; ^21^ University Hospital of Psychiatry Zürich, University of Zürich Zürich Switzerland; ^22^ Department of Psychiatry University Hospital of Lausanne (CHUV) Lausanne Switzerland; ^23^ Department of Psychiatry University of Oxford Oxford UK; ^24^ 1st Department of Neurology School of Medicine Laboratory of Neurodegenerative Diseases Center for Interdisciplinary Research and Innovation Aristotle University of Thessaloniki, and Alzheimer Hellas Thessaloniki Greece; ^25^ Institute of Neuroscience and Physiology Department of Psychiatry and Neurochemistry The Sahlgrenska Academy at University of Gothenburg Göteborg Sweden; ^26^ Paris Brain Institute ICM, Pitié‐Salpêtrière Hospital Sorbonne University Paris France; ^27^ Neurodegenerative Disorder Research Center Division of Life Sciences and Medicine and Department of Neurology Institute on Aging and Brain Disorders University of Science and Technology of China and First Affiliated Hospital of USTC Hefei PR China; ^28^ Department of Neurodegenerative Disease UCL Institute of Neurology Queen Square London UK; ^29^ UK Dementia Research Institute at UCL London UK; ^30^ Hong Kong Center for Neurodegenerative Diseases, N.T. Shatin Hong Kong China; ^31^ Wisconsin Alzheimer's Disease Research Center University of Wisconsin School of Medicine and Public Health, University of Wisconsin‐Madison Madison Wisconsin USA; ^32^ Translational Medicine Neuroscience UCB Biopharma SRL Brussels Belgium; ^33^ Currently at: Johnson & Johnson Innovative Medicines Beerse Belgium

**Keywords:** Alzheimer's disease (AD), amyloid, biomarker, blood, cerebrospinal fluid (CSF), DNA methylation, epigenetics, epigenome‐wide association study (EWAS), genome‐wide association study (GWAS), methylation quantitative trait loci (mQTL), mild cognitive impairment (MCI), neurofilament light (NfL), protein quantitative trait loci (pQTL), tau, YKL‐40

## Abstract

**INTRODUCTION:**

We investigated blood DNA methylation patterns associated with 15 well‐established cerebrospinal fluid (CSF) biomarkers of Alzheimer's disease (AD) pathophysiology, neuroinflammation, and neurodegeneration.

**METHODS:**

We assessed DNA methylation in 885 blood samples from the European Medical Information Framework for Alzheimer's Disease (EMIF‐AD) study using the EPIC array.

**RESULTS:**

We identified Bonferroni‐significant differential methylation associated with CSF YKL‐40 (five loci) and neurofilament light chain (NfL; seven loci) levels, with two of the loci associated with CSF YKL‐40 levels correlating with plasma YKL‐40 levels. A co‐localization analysis showed shared genetic variants underlying YKL‐40 DNA methylation and CSF protein levels, with evidence that DNA methylation mediates the association between genotype and protein levels. Weighted gene correlation network analysis identified two modules of co‐methylated loci correlated with several amyloid measures and enriched in pathways associated with lipoproteins and development.

**DISCUSSION:**

We conducted the most comprehensive epigenome‐wide association study (EWAS) of AD‐relevant CSF biomarkers to date. Future work should explore the relationship between *YKL‐40* genotype, DNA methylation, and protein levels in the brain.

**Highlights:**

- Blood DNA methylation was assessed in the EMIF‐AD MBD study.
- Epigenome‐wide association studies (EWASs) were performed for 15 Alzheimer's disease (AD)–relevant cerebrospinal fluid (CSF) biomarker measures.
- Five Bonferroni‐significant loci were associated with YKL‐40 levels and seven with neurofilament light chain (NfL).
- DNA methylation in *YKL‐40* co‐localized with previously reported genetic variation.
- DNA methylation potentially mediates the effect of single‐nucleotide polymorphisms (SNPs) in *YKL‐40* on CSF protein levels.

## 1 BACKGROUND

Because populations are now living for longer, the number of dementia cases is increasing, and it is estimated that by 2050 there will be 115 million people living with dementia worldwide. Alzheimer's disease (AD) is a chronic neurodegenerative disease that accounts for ~70% of dementia cases. The disease is characterized by the aggregation of two proteins: amyloid beta (Aβ), forming senile plaques, and hyperphosphorylated tau forming neurofibrillary tangles (NFTs).^2^, ^3^, ^4^ Pathology begins decades before symptoms appear, meaning that by the time an individual receives a clinical diagnosis there is already considerable neuropathology present.^5^, ^6^, ^7^, ^8^ In recent years there has been much research interest in detecting AD‐relevant biomarkers prior to the onset of clinical symptoms.^9^ Several different types of biomarkers could have utility for prodromal AD diagnosis. This includes the measurement of aberrant levels of proteins in the blood,^10^ cerebrospinal fluid (CSF),^11^, ^12^, ^13^ and brain^14^, ^15^ as well as imaging the structure and function of the brain.^16^, ^17^

CSF biomarkers have been used for diagnostic and monitoring purposes as the interstitial fluid of the brain is in direct contact with the CSF and, therefore, the CSF can offer an accurate reflection of disease progression. Through the sampling of CSF, it is possible to detect AD‐relevant changes including changes to the levels of Aβ40 or Aβ42,^18^ the Aβ40/42 ratio,^11^, ^12^ total tau (t‐tau) levels^19^ and phosphorylated tau (p‐tau) levels.^20^ More recently neurofilament light (NfL),^21^ neurogranin,^22^, ^23^ and chitinase‐3‐like protein 1 (CHI3L1, also known as YKL‐40)^24^ have also shown utility as CSF biomarkers for neurodegeneration, neuroinflammation, or synaptic damage in AD and other neurodegenerative diseases, and they are thus becoming more routinely measured in a research context. Lumbar puncture to collect CSF is less expensive in contrast to other methodologies such as imaging‐based techniques;^25^ however, it is still a relatively invasive process. As such, a number of studies have recently focused on identifying protein changes in the blood that mirror AD‐relevant protein changes in the CSF,^26^ with the view of developing a more accessible biomarker.

There is a growing interest in studying epigenetic mechanisms in AD, mediating interactions between genetic and environmental risks. Epigenetic processes regulate gene expression, with the most well characterized being that of DNA methylation. Several epigenome‐wide association studies (EWASs) have identified robust and reproducible alterations in DNA methylation in the AD brain.^27^, ^28^, ^29^, ^30^, ^31^, ^32^ Similarly, alterations in blood have been identified in individuals with AD or mild cognitive impairment (MCI) compared to non‐demented controls,^33^, ^34^, ^35^ or associated with cognitive performance in healthy older people.^36^ A recent study has used blood DNA methylation profiles as surrogates for modifiable and non‐modifiable risk factors for dementia, in order to assess dementia risk.^37^ Another recent study has identified blood DNA methylomic alterations associated with levels of three AD‐relevant biomarkers, namely Aβ42, p‐tau181, and t‐tau.^38^ In the current study we investigated whether DNA methylation patterns in the blood were associated with alterations in 15 AD‐relevant CSF biomarker levels and explored how these relate to previously described genetic variants associated with these traits in this cohort. Finally, we used network approaches to identify modules of co‐methylated loci associated with the different CSF biomarkers, performing gene ontology (GO) analysis to identify altered pathways.

## 2 METHODS

### 2.1 Subjects and samples

This study was undertaken as a part of the European Medical Information Framework for Alzheimer's Disease Multimodal Biomarker Discovery (EMIF‐AD MBD) study.^39^ One aim of EMIF‐AD MDB is to improve the access to, and use of, health‐related data, with emphasis on AD‐related biomarker research. To achieve this, the EMIF‐AD MBD study retrospectively combined clinical data, sample collections, biofluid‐based biomarker analyses, and imaging scans from 1221 subjects that had been collected across different centers, with details of this provided elsewhere.^39^ This cohort consisted predominantly of individuals of European ancestry. For our study, whole‐blood DNA samples were utilized from a subset of 953 participants, of which 805 were extracted locally at the respective recruitment centers and 148 at the University of Lübeck. In total 936 samples remained after DNA quality control (QC) which included agarose gel electrophoresis, determination of A260/280 and A260/230 ratios, and PicoGreen quantification.^40^

### 2.2 Bisulfite treatment and Illumina Infinium bead array

DNA was bisulfite treated using the EZ‐DNA methylation kit (Zymo Research, Orange, CA, USA). Samples were then analyzed using the Illumina Infinium Human Methylation EPIC BeadChip Array (EPIC array) (Illumina, USA), which interrogates >850,000 methylation sites throughout the genome, with all arrays run in one consecutive batch at the Institute of Clinical and Medical Biology (UKSH, Campus‐Kiel, Germany). For each probe on the array, DNA methylation levels were indexed by beta values, that is, the ratio of the methylated signal divided by the sum of the methylated and unmethylated signal (M/[M + U]).

**RESEARCH IN CONTEXT**

1. **Systematic review**: Robust and reproducible alterations in DNA methylation have been identified in the Alzheimer's disease (AD) brain, and more recently in the blood. However, studies exploring blood DNA methylation patterns associated with cerebrospinal fluid (CSF) biomarker measures are in their infancy.
2. **Interpretation**: Our work represents the largest epigenome‐wide association study (EWAS) of AD‐relevant CSF biomarker measures, both in terms of sample size (*N* = 885) and the number of biomarker measures assessed (*N* = 15). We showed evidence that DNA methylation in the *YKL‐40* gene may mediate the previously described protein quantitative trait loci (pQTL) for this gene, where single‐nucleotide polymorphisms (SNPs) have been reported to alter CSF protein levels.
3. **Future directions**: Our study has nominated several differentially methylated loci, which warrant further investigation as to their functional role in AD. In addition, future research should further explore how DNA methylation potentially mediates the *cis* effect of genetic variation on CSF YKL‐40 levels.

### 2.3 Microarray QC and data normalization

Initial QC of the resulting EPIC array data was conducted using GenomeStudio (version 2011.1) to visually check the status of staining, extension, hybridization, target removal, sodium bisulfite conversion, specificity, and non‐polymorphic and negative controls. Subsequently, IDAT files were loaded into R (version 3.5.2) using the methylumi package^41^ to create a *MethylumiSet* object. Samples were excluded from further steps if the mean background intensity of negative probes was <1000, the mean detection *P*‐values were >0.05, the bisulfite conversion efficiency was <80%, or there was a mismatch between reported and predicted sex. Sample and probe exclusion was performed using the *pfilter* function within the wateRmelon package;^42^ samples were removed with a detection *P* > 0.05 in more than 5% of probes, probes with <3 bead count in 5% of samples, and probes having 1% of samples with a detection *P* > 0.05. Duplicate and related samples were also removed. After QC, 861,666 probes and 885 samples remained. Quantile normalization was applied using the *dasen* function in the wateRmelon package.

### 2.4 Data analysis

In the current study, we utilized DNA methylation data generated in this subset of 885 subjects for whom various CSF biomarker measures had been collected in parallel with the EPIC DNA methylation profiles (Table 1). In total we analyzed 15 (partially correlated) biomarker traits in the current study, including tau CSF levels as quantitative (p‐tau assay Z‐score, t‐tau assay Z‐score) and binary variables (abnormal p‐tau, abnormal t‐tau), amyloid CSF levels as quantitative (Aβ38 levels, Aβ40 levels, Aβ42 levels, Aβ Z‐score, Aβ42/40 ratio) and binary variables (Aβ42/40 ratio dichotomized, amyloid status, abnormal Aβ42), and CSF levels of three other protein biomarkers of neurodegeneration (NfL), neuroinflammation (YKL‐40), and synaptic dysfunction (neurogranin), as well as disease status (control, MCI, AD). P‐tau assay Z‐score, t‐tau assay Z‐score, Aβ42 levels, and Aβ42/40 ratio were log‐transformed prior to analyses due to high inflation of the test statistics, as described previously.^40^ Some variables were correlated (Figure S1) and details on biomarker measurements can be found in Table 1. Samples with a biomarker value more than three standard deviations (SD) from the mean were excluded from the corresponding analysis. Linear regression analyses were performed to examine the association of DNA methylation with each of the 15 CSF biomarker variables, while controlling for the effects of age, sex, bisulfite plate, center, the first four genetic principal components (PCs: as described in ref.^40^), and cell type proportions (calculated using the *estimateCellCounts* function in the minfi package^43^). Our samples were predominantly of European ancestry (*N* = 864 + 11 outliers), with a small number of individuals of African (*N* = 1), Hispanic (*N* = 3), East Asian (*N* = 1 + 2 outliers), or South Asian (*N* = 3) descent, and the inclusion of the four genetic PCs captured and controlled for this ethnic diversity. Bonferroni significance was used to account for multiple testing of 861,666 CpG probes (*P* < 5.80 × 10^−8^). We also repeated these analyses with disease status as a categorical variable (control, MCI, AD) as an additional covariate to control for diagnosis, which could confound results. To investigate the association of blood DNA methylation with disease status we used an analysis of variance (ANOVA), with post hoc Tukey's Honest Significant Difference (HSD) tests to identify differentially methylated positions (DMPs) between the different groups, which adjusts for the number of comparisons with adjusted *P* values (*P*_adj_) that account for the three comparisons reported. To identify differentially methylated regions (DMRs) consisting of multiple adjacent DMPs we used comb‐p^44^ with a distance of 500 bp and a seeded *P* = 1.0 × 10^−4^, with DMRs defined as containing at least three probes and having a Šidák‐corrected *P* < 0.05 (herein referred to as *P*_adj_). DMPs and DMRs were annotated in tables using both the Illumina (UCSC) gene annotation (which is derived from the genomic overlap of probes with RefSeq genes or up to 1500 bp of the transcription start site [TSS] of a gene) and the “Genomic Regions Enrichment of Annotations Tool” (GREAT)^45^ annotation (which annotates a probe to genes with a TSS within 5 kb upstream or 1 kb downstream).

**TABLE 1:** Demographic information on the samples and CSF measures included in the analysis.

	Variable	Description	Variable type	Number of samples	Diagnosis (C/MCI/AD)	Transformation	Units	Measure, mean (± SD)	Normal/ Abnormal	Age, mean (± SD)	Gender M/F
**Tau Protein**	t‐tau assay Z‐score	Z‐score for CSF t‐tau pathology (local)	Quantitative	701	158/396/147	Log‐transformed	NA	−0.671 (± 1.25)	NA	68.80 (± 8.65)	336/365
	Abnormal t‐tau	Dichotomous CSF t‐tau assessment (local)	Binary	718	160/400/158	NA	NA	NA	342/376	68.82 (± 8.67)	341/377
	p‐tau assay Z‐score	Z‐score for CSF p‐tau pathology (local)	Quantitative	694	158/393/143	Log‐transformed	NA	‐0.457 (± 1.15)	NA	68.76 (± 8.65)	331/363
	Abnormal p‐tau	Dichotomous CSF p‐tau assessment (local)	Binary	719	160/400/159	NA	NA	NA	371/348	68.82 (± 8.66)	341/378
**Amyloid Protein**	Abnormal Aβ42	Dichotomous CSF Aβ42 assessment (local)	Binary	719	160/400/159	NA	NA	NA	329/390	68.82 (± 8.66)	341/378
	Aβ42	Aβ42 CSF assessment (central)	Quantitative	660	118/390/152	Log‐transformed	pg/ml	292.54 (± 158.17)	NA	69.46 (± 8.38)	315/345
	Aβ40	Aβ40 CSF assessment (central)	Quantitative	668	121/395/152	NA	pg/ml	5001.67 (± 1753.25)	NA	69.44 (± 8.36)	322/346
	Aβ38	Aβ38 CSF assessment (central)	Quantitative	663	121/391/151	NA	pg/ml	2143.63 (± 811.29)	NA	69.46 (± 8.39)	318/345
	Aβ Z‐score	Z‐score for CSF Aβ pathology (central)	Quantitative	879	321/399/159	NA	NA	‐0.47 (± 1.02)	NA	68.82 (± 8.27)	431/448
	Aβ42/40 ratio	Ratio of CSF Aβ42/40 (central)	Quantitative	672	121/397/154	Log‐transformed	NA	0.060 (± 0.02)	NA	69.47 (± 8.36)	323/349
	Aβ42/40 ratio dichotomized	Dichotomous CSF Aβ42/40 ratio (central)	Binary	673	121/398/154	NA	NA	NA	259/414	69.46 (± 8.36)	324/349
	Amyloid status	Dichotomous amyloid classification	Binary	885	324/402/159	NA	NA	NA	427/458	68.80 (± 8.25)	435/450
**Other**	YKL‐40	YKL‐40 CSF assessment (central)	Quantitative	664	120/394/150	NA	pg/mL	169221.50 (± 59991.00)	NA	69.30 (± 8.33)	320/344
	NfL	Neurofilament light CSF assessment (central)	Quantitative	662	121/389/152	NA	pg/mL	1060.38 (± 762.18)	NA	69.45 (± 8.38)	316/346
	Neurogranin	Neurogranin CSF assessment (central)	Quantitative	613	100/368/145	NA	pg/mL	131.95 (± 117.08)	NA	69.49 (± 8.40)	286/327
	Diagnosis	Diagnosis of control, MCI, or AD	Categorical	885	324/402/159	NA	NA	NA	NA	68.80 (± 8.25)	435/450

*Note*: Sample numbers, the mean of each measure, the split of normal/abnormal classification, the mean age at which the measure was taken, and the split of males (M) and females (F) (M/F) is shown for each of the 15 measures taken as a part of this study. The terms “local” and “central” refer to the location of the data analysis, with “local” analyses being undertaken separately at each collection site and “central” analyses being undertaken together at one central location. If both “local” and “central” measures were available for a given CSF variable, we used the “central” measure for our analyses. NA refers to not applicable in that assessment. The cohort was predominantly of European ancestry (*N* = 875), with 10 individuals from other ethnic backgrounds (African: *N* = 1, Hispanic: *N* = 3, East Asian: *N* = 3, South Asian: *N* = 3).

Abbreviations: Aβ, amyloid beta; AD, Alzheimer's disease; CSF, cerebrospinal fluid; F, female; MCI, mild cognitive impairment; M, male; NA, not applicable; NfL, neurofilament light; SD, standard deviation.

### 2.5 Overlap with genetic variants identified from CSF biomarker GWAS

Linkage disequilibrium (LD) regions were generated for significant SNPs from the Hong et al. ^40^ and Hong et al. ^46^ genome‐wide association studies (GWASs) using the *LDproxy* function in the LDlinkR package^47^ using a *D* value of 0.1 to determine LD regions. For each biomarker EWAS, we took forward all probes that reached a *P*‐value threshold of < 1 × 10^−4^ and assessed whether these fell into the LD region for their associated biomarker analysis from the GWAS analyses.

In addition, we conducted a Bayesian co‐localization test to demonstrate co‐localization between the genomic region associated with the CSF YKL‐40 marker (chr1:203115267:203181560), as reported in the GWAS by Hong et al.^46^ and CpGs annotated to *CHI3L1* (*YKL‐40)*. We obtained *cis*‐methylation quantitative trait loci (mQTLs: genetic variation affecting DNA methylation) with a *P*‐value threshold of < 1 × 10^−5^ associated with eight DMPs linked to CSF YKL‐40 from the Genetics of DNA Methylation Consortium (goDMC).^48^ We tested for a potential pleiotropic effect between DNA methylation and CSF protein markers using the coloc.abf function in the coloc R package (version 5.1.0.1).^49^ A posterior probability larger than 0.99 for having one common causal variant for both traits in the co‐localization analysis was considered for further causal inference tests (CITs).

We examined the mediation of DNA methylation on the association between the SNPs in *CHI3L1* (*YKL‐40*) and CSF YKL‐40 levels using the R package ‘cit’^50^ and the ‘cit.cp’ function with 500 permutations for conditional analysis. The overall *P*‐value (omnibus *p*‐value) was reported based on collective conditional analysis, and a threshold of *P*  <  0.05 was considered nominally significant. In order to focus on independent genetic variants within the genomic region of interest, we performed clumping on the CSF *YKL‐40* GWAS summary stats reported by Hong et al.^46^ with the following parameters: –clump‐p1 1.0E‐5 –clump‐p2 0.05 –clump‐r2 0.6 –clump‐kb 250.

### 2.6 Assessment of plasma YKL‐40 protein levels

Plasma YKL‐40 protein levels were assessed in a subset of 568 of the subjects using the Somalogic 4k array on the SOMAscan assay platform as described previously.^51^ Data were COMBAT normalized to remove batch effects^52^ and, after outlier removal, 560 samples were taken forward for analysis. To control for confounding variables, the effects of age, sex, center, the first four genetic PCs (to control for ethnicity), and cell proportions (estimated in the DNA methylation data) were regressed out. We then used Pearson's correlation to assess the correlation between plasma YKL‐40 protein levels and DNA methylation levels for the Bonferroni‐significant differentially methylated loci in the *YKL‐40* gene. Subsequently, we examined the correlation between plasma YKL‐40 protein levels and CSF YKL‐40 protein levels. For this analysis we also regressed out the effects of age, sex, center, and genetic PCs from the CSF YKL‐40 protein data.

### 2.7 Weighted gene correlation network analysis (WGCNA)

Weighted gene correlation network analysis (WGCNA) was performed to identify modules of highly co‐methylated probes using the *blockwiseModules* function in the WGCNA package.^53^ Analyses were performed on the normalized data, where the effects of confounding variables identified in Section 2.4 were removed (e.g., age, sex, bisulfite plate, center, the first four genetic PCs, cell type proportions). We performed un‐signed analyses to determine correlations in positive and negative directions, on data regressed for all covariates (listed previously). Generated modules were then correlated with all diagnosis and biomarker phenotypic measures, and modules that reached a multiple testing threshold of *P* < 9.62 × 10^−4^ (to account for the number of modules) were taken forward for downstream analyses. Hub probes were identified with the *chooseTopHubInEachModule* function.

Genes annotated to the significantly associated modules were analyzed using the STRING Database^54^, ^55^ to determine protein–protein interactions (PPIs). GO pathway analysis was performed on significantly associated modules using the *gometh* function from the missMethyl package^56^ to apply gene set testing on GO categories.

### 2.8 Data and code availability

Genome‐wide DNA methylation data for the 885 EMIF‐AD MBD samples are stored on an online data platform using the “tranSMART” data warehouse framework. Access can be requested from the corresponding author who will forward the request to the EMIF‐AD data access team. All analytical scripts/code are available at https://github.com/UoE‐Dementia‐Genomics/EMIF_Biomarkers_Methylation

## 3 RESULTS

### 3.1 EWAS on disease status

First, we used an ANOVA, with post hoc Tukey's HSD tests to compare DNA methylation differences between the three diagnostic groups: control, MCI, and AD (Table S1). Although no probes passed our stringent Bonferroni significance threshold, the most significant DMP was annotated to the gene body of *OLFM3* (cg03104428: *F* = 10.58, *P* = 2.89 × 10^−5^), and was nominally significantly differentially methylated between control and AD (*P*_adj_ = 9.58 × 10^−3^) and MCI and AD subjects (*P*_adj_ = 1.53 × 10^−5^). Of interest, OLFM3 was recently identified, and independently replicated, in an ultra‐deep CSF proteomics study as a novel AD biomarker, in addition to being elevated in both MCI and AD post‐mortem human brain samples.^57^ Our regional analysis, to identify DMRs consisting of multiple adjacent DMPs, did not reveal any significant regions.

### 3.2 EWAS on CSF tau variables

Next, we performed EWASs exploring the association of DNA methylation with our four CSF tau variables (t‐tau assay Z‐score: Table S2, abnormal t‐tau: Table S3, p‐tau assay Z‐score: Table S4, abnormal p‐tau: Table S5). Although we did not identify any significant DMPs after Bonferroni correction in these analyses, a number of loci nearly passed this conservative significance threshold. Given that there was a modest correlation between these measures and disease status (Figure S1), we also performed EWASs, where we additionally controlled for this covariate, although this did not substantially affect the results (Tables S2–S5). Our regional analysis identified three significant DMRs associated with p‐tau assay Z‐score (Table S6A), including a four‐probe DMR residing in *LINC00857* (Figure S2A: *P*_adj_ = 1.97 × 10^−6^), a three‐probe DMR residing in *C3* (Figure S2B: *P*_adj_ = 3.77 × 10^−6^), and an eight‐probe DMR located 191 bp from the *CMYA5* gene transcription start site (TSS) (Figure S2C: *P*_adj_ = 8.72 × 10^−6^). We also identified three significant DMRs associated with abnormal p‐tau levels (Table S6B), which included a 10‐probe DMR residing in *S100A13* (Figure S3A: *P*_adj_ = 8.48 × 10^−8^), a 13‐probe DMR in *ZBTB22* (Figure S3B: *P*_adj_ = 4.27 × 10^−6^), and a four‐probe DMR in *SPATS2* (Figure S3C: *P*_adj_ = 6.72 × 10^−6^). We did not identify any DMRs associated with the t‐tau assessments.

### 3.3 EWAS on CSF amyloid measures

Next, we probed for DMPs associated with eight CSF amyloid measures (abnormal Aβ42: Table S7, Aβ42 levels: Table S8, Aβ40 levels: Table S9, Aβ38 levels: Table S10, Aβ Z‐score: Table S11, Aβ42/40 ratio: Table S12, Aβ42/40 ratio dichotomized: Table S13, and amyloid status: Table S14). As for the analyses with CSF tau variables, none passed the conservative Bonferroni‐significance threshold. However, a number of significant DMRs emerged for the various amyloid measures. We identified five DMRs associated with abnormal Aβ42 (Table S15A), including five probes residing in *MX2* (Figure S4A: *P*_adj_ = 1.35 × 10^−11^), five probes in *ABCG2* (Figure S4B: *P*_adj_ = 1.29 × 10^−8^), four probes in *RHOH* (Figure S4C: *P*_adj_ = 9.47 × 10^−8^), 12 probes in *ZBTB22* (Figure S4D: *P*_adj_ = 2.98 × 10^−5^), and a four‐probe DMR in *SLFN12* (Figure S4E: *P*_adj_ = 4.30 × 10^−5^). We identified eight DMRs associated with Aβ42 levels (Table S15B), including six probes in *AKR1E2* (Figure S5A: *P*_adj_ = 2.05 × 10^−7^), seven probes in *ADHFE1* (Figure S5B: *P*_adj_ = 5.15 × 10^−7^), five probes in *ANKMY1* (Figure S5C: *P*_adj_ = 7.68 × 10^−5^), seven probes in *RGMA* (Figure S5D: *P*_adj_ = 4.69 × 10^−6^), five probes in *RBBP7* (Figure S5E: *P*_adj_ = 1.04 × 10^−4^), four probes in *LOC101929241* (Figure S5F: *P*_adj_ = 3.11 × 10^−4^), three probes in *MX2* (Figure S5G: *P*_adj_ = 9.63 × 10^−4^), and eight probes in *VARS2* (Figure S5H: *P*_adj_ = 5.62 × 10^−3^). In our Aβ40 analysis we identified two DMRs (Table S15C), including five probes residing 18 bp from the *TGFBI* gene TSS (Figure S6A: *P*_adj_ = 1.82 × 10^−7^) and five probes in *ANKMY1* (Figure S6B: *P*_adj_ = 5.92 × 10^−7^). Two DMRs were identified in the Aβ38 analysis (Table S15D), which included four probes in *STRA6* (Figure S7A: *P*_adj_ = 1.27 × 10^−7^) and five probes in *TGFBI* (Figure S7B: *P*_adj_ = 5.93 × 10^−7^). The Aβ Z‐score analysis identified four DMRs (Table S15E), which included five probes in *MX2* (Figure S8A: *P*_adj_ = 1.61 × 10^−12^), 15 probes in *ZFP57* (Figure S8B: *P*_adj_ = 2.61 × 10‐^12^), six probes in *FURIN* (Figure S8C: *P*_adj_ = 1.73 × 10^−6^), and five probes in *CD24* (Figure S8D: *P*_adj_ = 4.11 × 10^−6^). We identified one 12‐probe DMR associated with Aβ42/40 ratio (Table S15F), which resided in *ZFP57* (Figure S9: *P*_adj_ = 7.13 × 10^−8^). Similarly, we identified one five‐probe DMR in the amyloid status analysis (Table S15G), which resided in *MX2* (Figure S10: *P*_adj_ = 2.14 × 10^−12^).

### 3.4 EWAS on other disease‐relevant CSF biomarkers

**FIGURE 1:**
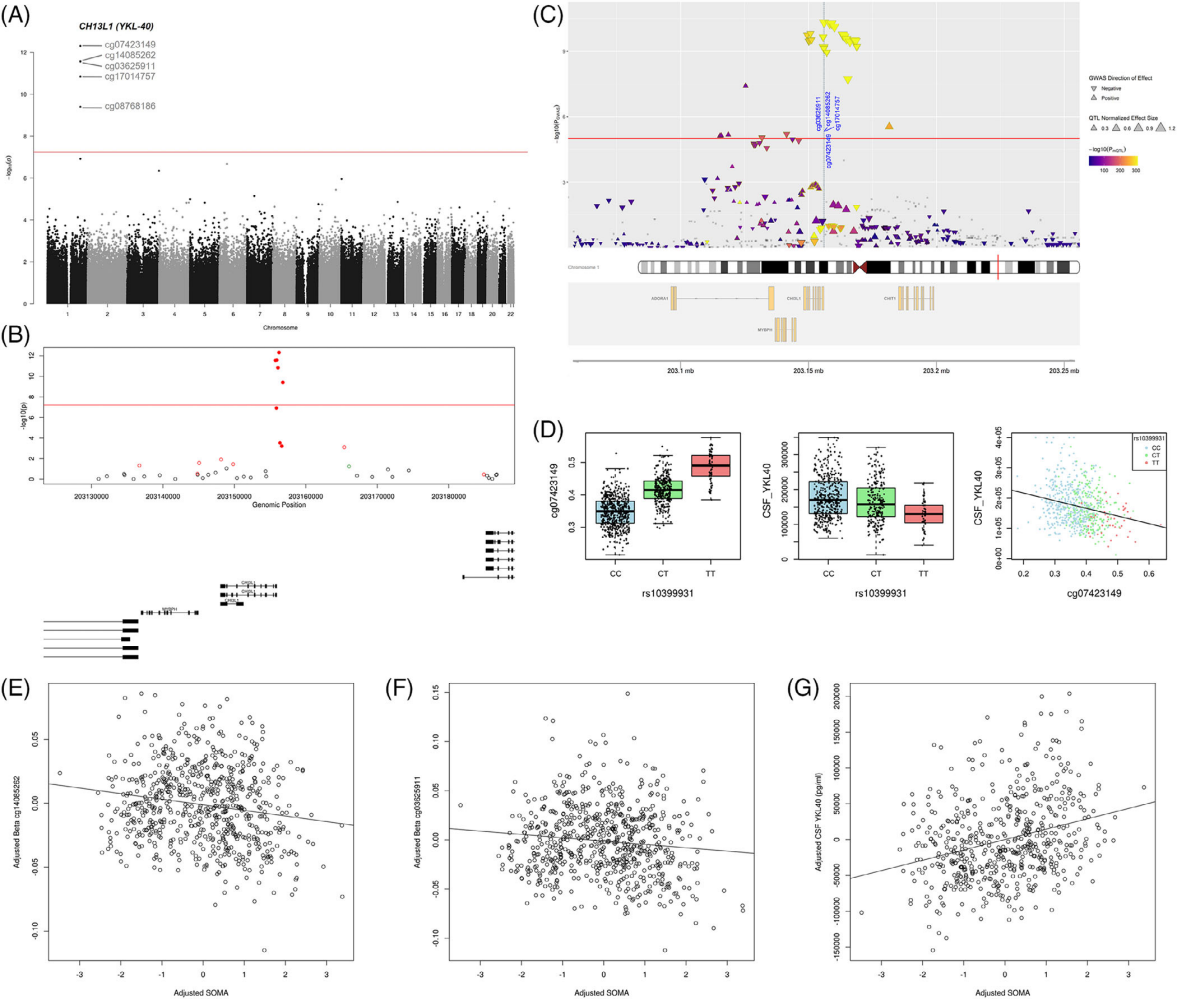
A DMR in the *CHI3L1* gene in blood is associated with CSF YKL‐40 levels. (A) A Manhattan plot highlighting five Bonferroni‐significant DMPs in the *CHI3L1 (*YKL‐40) gene identified from linear regression analysis. The X‐axis shows chromosomes 1–22, followed by the X (23) and Y (24) chromosomes, whereas the Y‐axis shows –log10(p). The horizontal red line denotes Bonferroni significance (*P* = 5.80 × 10^−8^). The 1000 most significant DMPs can be found in Table S16. (B) The most significant DMR in the YKL‐40 analysis featured eight probes in the *CHI3L1 (YKL*‐*40)* gene (chr1:203155737‐203156784). The X‐axis shows genomic position, whereas the Y‐axis shows –log10(p). A horizontal red line denotes Bonferroni significance (*P* = 5.80 × 10^−8^). Red probes represent a positive effect size (ES) ≥1%, green probes represent a negative ES ≥1% across the range of the analysis. Filled circles denote the probes in the DMR. ES is defined as the % methylation difference across the range of values. Underneath the gene tracks are shown in black. Full details on the DMR can be found in Table S19A. (C) Co‐localization analysis of CSF YKL‐40–associated differential methylation and its GWAS‐nominated genomic region. Shown are overlapping SNPs observed between the region reported in the GWAS of CSF *YKL‐40* levels by Hong et al. and *cis*‐mQTLs near cg03625911, cg14085262, cg07423149, and cg17014757, also corresponding to our four most significant DMPs in our EWAS. (D) Evidence that DNA methylation in *CH13L1* (*YKL‐40*) may mediate the association between SNP variation and CSF protein levels, shown as an example for cg07423149 and rs10399931. Shown are a robust mQTL corresponding to the association between genotype at rs10399931 (CC, CT, TT) and DNA methylation at cg07423149 (corrected beta levels) (left panel), a robust pQTL corresponding to the association between rs10399931 genotype and CSF YKL‐40 levels (middle panel), and the correlation between DNA methylation at cg07423149 and CSF YKL‐40 levels, colored by genotype at rs10399931 (right panel). Correlation of blood DNA methylation at (E) cg14085262 and (F) cg03625911 in the *YKL‐40* gene (Y‐axis) with YKL‐40 plasma protein levels (X‐axis), with both DNA methylation and protein data. (G) Correlation of CSF YKL‐40 protein levels (Y‐axis) with plasma protein levels (X‐axis). Data shown in E–G have been regressed for the effects of covariates. Abbreviations: CSF, cerebrospinal fluid; DMPs, differentially methylated positions; DMR, differentially methylated region; ES, effect size; EWAS, epigenome‐wide association study; GWAS, genome‐wide association study; mQTL, methylation quantitative trait loci; pQTL, protein quantitative trait loci; SNP, single‐nucleotide polymorphism.

**FIGURE 2:**
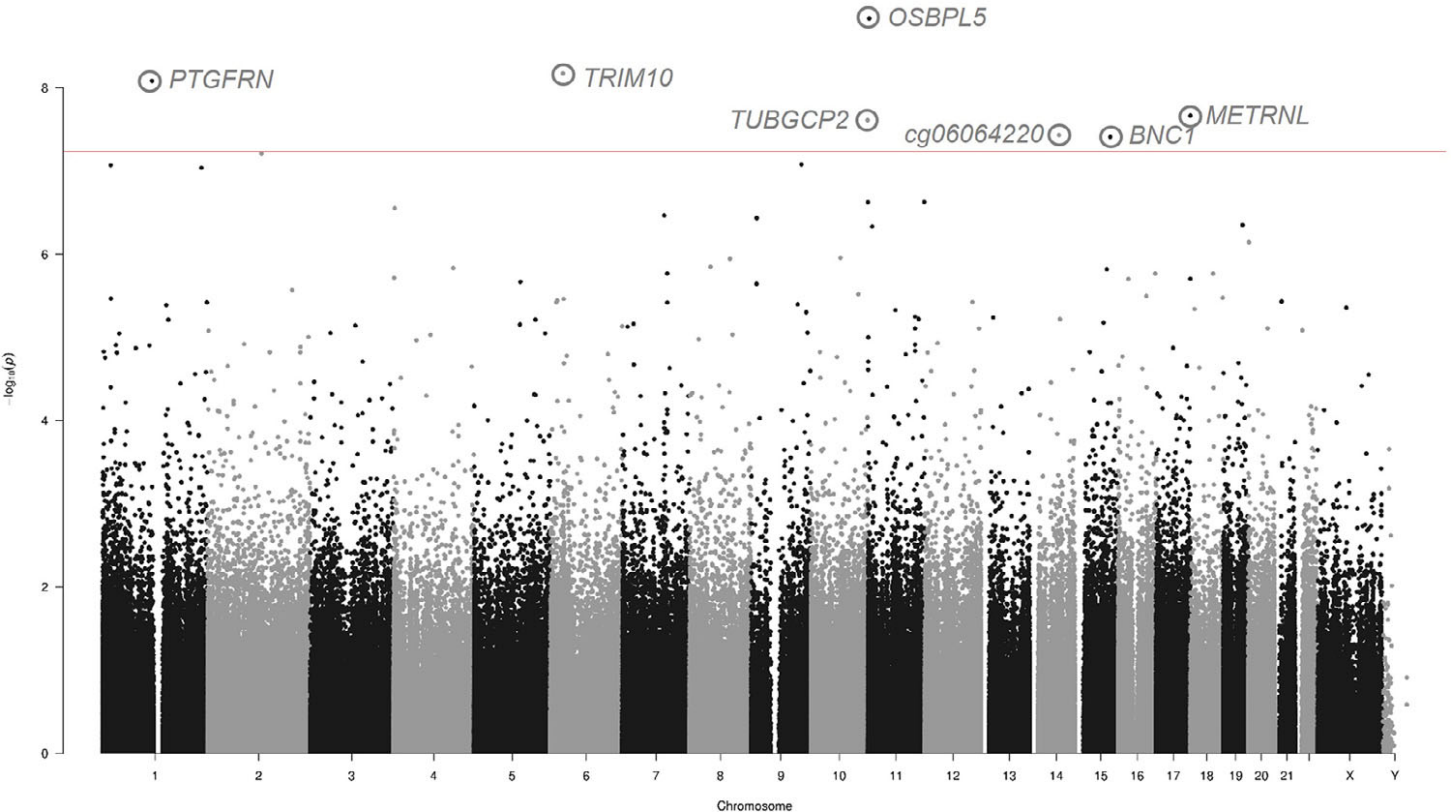
Differential methylation in the blood is associated with CSF NfL levels. Shown is a Manhattan plot, with the seven Bonferroni‐significant DMPs labeled with gene name or cg ID (if unannotated). The X‐axis shows chromosomes 1–22, followed by the X (23) and Y (24) chromosomes, whereas the Y‐axis shows –log10(p). The horizontal red line denotes Bonferroni significance (*P* = 5.80 × 10^−8^). Abbreviations: CSF, cerebrospinal fluid; DMPs, differentially methylated positions; NfL, neurofilament light.

Aside from CSF tau and amyloid measures, we also had available CSF biomarkers of neuroinflammation (YKL‐40), neurodegeneration (NfL), and synaptic dysfunction (neurogranin). In our EWAS of YKL‐40 (Table S16 and Figure 1A), we identified five Bonferroni‐significant probes, all residing within ~1 kb of each other in the *CHI3L1* gene, which encodes the YKL‐40 protein (cg07423149: *P *= 4.91 × 10^−13^, cg14085262: *P *= 2.62 × 10^−12^, cg03625911: *P *= 2.77 × 10^−12^, cg17014757: *P *= 1.44 × 10^−11^, cg08768186: *P *= 3.99 × 10^−10^). All these probes remained significant after including disease status as an additional covariate. For the EWAS on NfL, we identified seven Bonferroni‐significant loci (Table S17 and Figure 2), (cg16073540 [*OSBPL5*]: *P *= 1.46 × 10^−9^, cg24329658 [*TRIM10*]: *P *= 6.71 × 10^−9^, cg26422266 [*PTGFRN*]: *P *= 8.28 × 10^−9^, cg12817352 [*METRNL*]: *P *= 2.16 × 10^−8^, cg16625929 [*TUBGCP2*]: *P *= 2.45 × 10^−8^, cg06064220 [unannotated]: *P *= 3.69 × 10^−8^, cg14894702 [*BNC1*]: *P *= 3.89 × 10^−8^), with all except cg16625929 remaining significant after controlling for disease status. We did not identify any Bonferroni‐significant loci associated with neurogranin (Table S18).

Our regional analysis identified three significant DMRs associated with YKL‐40 levels (Table S19A), including an eight‐probe DMR within the *CH13L1* (*YKL‐40*) gene (Figure 1B: *P*_adj_ = 1.51 × 10^−47^), a seven‐probe DMR in the *HS3ST3B1* gene (Figure S11A: *P*_adj_ 1.83 × 10^−8^), and a four‐probe DMR in *CYP26C1* (Figure S11B: *P*_adj_ = 6.68 × 10^−3^). The NfL regional analysis identified eight significant DMRs (Table S19B), including a six‐probe DMR 24 bp from the *TEX12* TSS (Figure S12A: *P*_adj_ = 1.85 × 10^−11^), eight probes in the *SORD* gene (Figure S12B: *P*_adj_ = 1.26 × 10^−9^), ten probes in the *S100A13* gene (Figure S12C: *P*_adj_ = 8.47 × 10^−9^), three probes in *CCDC71L* (Figure S12D: *P*_adj_ = 2.08 × 10^−7^), five probes in *NAALAD2* (Figure S12E: *P*_adj_ = 1.43 × 10^−6^), five probes in *STK16* (Figure S12F: *P*_adj_ = 3.00 × 10^−6^), three probes in *CNTN3* (Figure S12G: *P*_adj_ = 5.06 × 10^−3^), and four probes in *PRDM9* (Figure S12H: *P*_adj_ = 6.28 × 10^−3^). Finally, the neurogranin regional analysis identified four significant DMRs (Table S19C), including six probes in *AVP* (Figure S13A: *P*_adj_ = 1.52 × 10^−7^), seven probes in *SORD* (Figure S13B: *P*_adj_ = 1.11 × 10^−5^), four probes in *STRA6* (Figure S13C: *P*_adj_ = 1.45 × 10^−5^) and a three‐probe DMR in *FAR2* (Figure S13D: *P*_adj_ = 4.43 × 10^−5^).

### 3.5 Eight DMRs overlap between different CSF biomarker analyses

**FIGURE 3:**
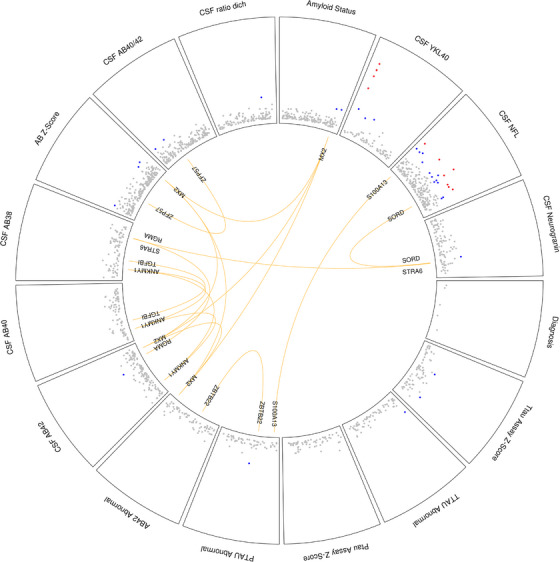
A number of common DMRs featured in more than one CSF biomarker analysis. Three DMRs overlapping in CSF tau analyses, four other DMRs overlapping in CSF amyloid analyses, and four DMRs overlapping between different CSF protein biomarker analyses. Each segment on the plot shows probes that pass different significant thresholds (gray < 1 × 10^−4^, blue < 1 × 10^−6^, red < Bonferroni threshold) with orange lines connecting overlapping or identical DMRs from different CSF analysis. Abbreviations: CSF, cerebrospinal fluid; DMRs, differentially methylated regions.

We identified eight overlapping or identical DMRs that featured in more than one of the CSF biomarker analyses (Figure 3). The DMR spanning three or five overlapping probes in *MX2* featured in four of the amyloid analyses (abnormal Aβ42, Aβ42 levels, Aβ Z‐score, amyloid status), which are measures that all show a modest degree of correlation (Figure S1). Several other DMRs featured in more than one of the amyloid analyses. A five‐probe DMR was identified in *ANKMY1* in the Aβ42 and Aβ40 levels analyses, which are measures with a modest degree of correlation (*r *= 0.62). The five‐probe DMR in *TGFBI* found in the Aβ40 and Aβ38 levels analyses, and the DMR spanning at least 12 probes in the *ZFP57* gene in the Aβ Z‐score and Aβ42/40 ratio analyses, are not surprising given that these measures are very highly correlated. Of interest, we identified common DMRs between different types of CSF biomarkers. A four‐probe DMR in *STRA6* was identified in the Aβ38 levels and neurogranin levels analyses, which are measures with a modest degree of correlation (Figure S1: *r* = 0.49). It is interesting to note that we identified some common DMRs associated with biomarker measures that showed a lower degree of correlation. An overlapping DMR spanning 12 or 13 probes in the *ZBTB22* gene was identified in the abnormal Aβ42 and abnormal p‐tau analyses, which are measures that show a more modest degree of correlation (Figure S1: *r* = 0.33). The overlapping DMR of seven or eight probes in *SORD* in the NfL and neurogranin analyses was of interest, as these measures show a low degree of correlation (Figure S1: *r* = 0.22). Similarly, abnormal p‐tau and NfL levels have a low degree of correlation (Figure S1: *r* = 0.18), yet we identified an identical 10‐probe DMR in *S100A13*.

### 3.6 Overlap with GWAS results from AD biomarker analyses

Previous work from our group has identified genome‐wide associations between several genetic variants and the CSF biomarker measurements in a largely overlapping portion of the EMIF cohort.^40^, ^46^ Here, we were interested in investigating whether the genomic loci identified in these prior GWAS may also show signals of differential methylation, which could suggest that DNA methylomic variation is driven by SNP variation in *cis*. Given that for the majority of our EWAS, we did not identify any Bonferroni‐significant DMPs and as this represented a candidate‐based analysis, we used a less conservative threshold (*P* < 1 × 10^−4^) to identify DMPs from the EWAS that resided in genomic regions where SNPs had been identified in the GWAS for each respective biomarker. For the tau analyses we identified four DMPs that co‐localized with reported GWAS signals. This included one DMP in *GALNT14* (cg14501323) in the t‐tau Z‐score analysis (Table S20A), one DMP in *TMEM136* (cg22210493) in the abnormal t‐tau analysis (Table S20B), and two DMPs located in, or proximal to, the *ANKRD11* gene (cg07619583, cg17289913) in the abnormal p‐tau analysis (Table S20C). We identified co‐localization of DMPs with GWAS hits for seven of the eight amyloid biomarker measures. In the Aβ42 levels analysis we identified co‐localized DMPs in *AKAP8L* (cg27090975), *PPP6R2* (cg18512769), and *HDAC9* (cg27365190) (Table S21A). In the Aβ40 levels analysis we identified three co‐localized DMPs (Table S21B), residing in *TUBGCP6* (cg09169375), *ZNF254* (cg09777776), and one unannotated, but located ~1 kb from the *FAM171A1* gene (cg02680903). For the Aβ38 levels analysis two co‐localized DMPs were identified (Table S21C), one located in *OAS2* (cg11318133) and one in *TUBGCP* (cg09169375), which co‐localized with the same SNP we had identified in the Aβ40 level co‐localization analysis. In the Aβ Z‐score analysis, three DMPs co‐localized with the GWAS signals (Table S21D), including the *APOC1* gene (cg13880303), the *UBXN10/UBXN10‐AS1* region (cg18722282), and *HLA‐DMA* (cg22961241). The same probe in *APOC1* was also identified in the Aβ42/40 ratio co‐localization analysis (and had been the most significant probe in the EWAS: *P* = 2.71 × 10^−7^), along with a probe in *APOE* (cg06750524), (Table S21E). We identified one co‐localized probe in the Aβ42/40 ratio dichotomized analysis (Table S21F) in *CCL20* (cg25384507) and one in the amyloid status analysis (Table S21G) that was unannotated, but located near the *APOC1* region and co‐localized with many of the same SNPs that were identified in the Aβ Z‐score and Aβ42/40 ratio analyses. We identified eight DMPs in the NfL analysis that co‐localized with the corresponding GWAS (Table S22A), including two unannotated loci (cg23455685, cg14464583), and loci in *TMEM232* (cg10285888), *ATXN1* (cg07885635), *TMEM106B* (cg05433004), *ZNF385C* (cg27283039), *PPP1R2* (cg24326567), and *SORD* (cg00891891), which interestingly resided in the DMR identified in the NfL and neurogranin analyses. We also identified two DMPs in the neurogranin analysis that co‐localized with the previous GWAS (Table S22B), located in *GPSM1* (cg14203108) and *PAEP* (cg12053709).

It is notable that for YKL‐40 we observed co‐localization of the five Bonferroni‐significant DMPs and one additional DMP in *CH13L1* (*YKL‐40*) with SNPs in this region reported in the corresponding GWAS (Table S22C), suggesting that the methylation patterns we observed are driven by genetic variation at this locus. To explore this further we used a Bayesian approach to test whether the risk variants residing in the genomic region associated with CSF *YKL‐40* (reported by Hong and colleagues [chr1:203115267‐203181560]^46^) are associated with the DMPs we had identified in this gene. Taking forward eight DMPs corresponding to *cis*‐mQTLs (*P* < 1 × 10^−5^) in this region in the goDMC database,^48^ we tested for possible pleiotropic effects between DNA methylation and CSF protein levels. We identified common causal variants associated with CSF YKL‐40 levels and DNA methylation at six CpG sites in this gene (cg03625911, cg02097014, cg07423149, cg14085262, cg17014757, and cg19081101), (Table S23 and Figure 1C) and so examined this further with CIT. Indeed, DNA methylation at all six CpG sites showed evidence of mediating the association between genetic variation and CSF YKL‐40 levels (Table S24 and Figure 1D).

### 3.7 Relationship of YKL‐40 blood DNA methylation with plasma protein levels

Next, as we had related DNA methylation patterns in blood to CSF protein levels in the CSF, we decided to explore whether there was a relationship with plasma YKL‐40 levels. For the five Bonferroni‐significant differentially methylated loci we had identified in our CSF YKL‐40 EWAS, two of these showed a significant correlation with plasma YKL‐40 levels (cg14085262: *r* = −0.17, *P *= 1.02 × 10^−5^, Figure 1E; cg03625911: *r* = −0.11, *P* = 3.23 × 10^−3^; Figure 1F). Furthermore, we observed a significant correlation between plasma YKL‐40 levels and CSF YKL‐40 levels (*r* = 0.266, *P* = 1.84 × 10^−10^, Figure 1G).

### 3.8 Blood DNA co‐methylation networks associated with CSF biomarker measurements

**FIGURE 4:**
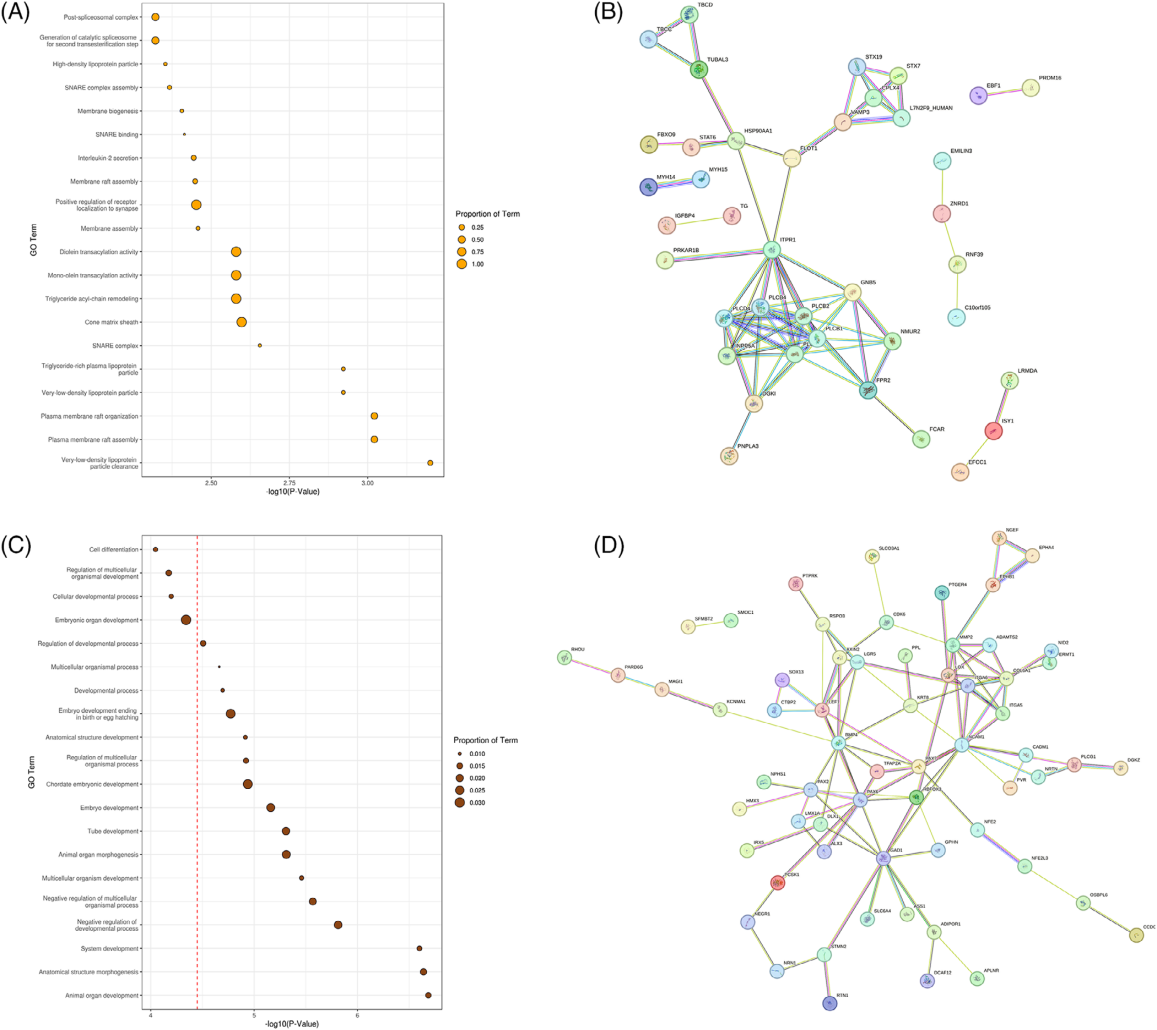
WGCNA identified two significant blood DNA methylation modules associated with CSF biomarker levels. (A) Shown are the top 20 most significant terms for GO enrichment analyses in the orange module, consisting of 167 probes, which was Bonferroni significantly associated with CSF Aβ42, Aβ40, and Aβ38 levels. The term titles are displayed along the Y‐axes, with –log10(P) for enrichment significance shown along the X‐axis. Points are sized by the proportion of the overall GO numbers represented in that specific module. (B) PPI analysis of the genes annotated to the module highlighted several proteins that were highly connected, including ITPR1 and HSP90AA1. (C) Shown are the top 20 most significant GO terms for the saddlebrown module, consisting of 157 probes, which was Bonferroni significantly associated with CSF Aβ42/40 ratio dichotomized, as well as nominally significantly associated with abnormal Aβ42 levels, Aβ Z‐score, Aβ42/40 ratio, and amyloid status. The term titles are displayed along the Y‐axis, with –log10(P) for enrichment significance shown along the X‐axis. The red dashed vertical line denotes the FDR significance threshold. (D) PPI analysis identified several highly connected proteins, including NCAM1. Abbreviations: Aβ, amyloid beta; CSF, cerebrospinal fluid; FDR, false discovery rate; GO, gene ontology; PPI, protein–protein interaction; WGCNA, weighted gene correlation network analysis.

Next we investigated whether any loci showed co‐methylation patterns that were correlated with the various CSF biomarker measures. To this end, we used WGCNA and identified 52 modules of highly co‐methylated probes before testing their association with disease status and the 15 CSF biomarker measurements (Figure S14). These analyses revealed two modules that showed a significant correlation with our traits of interest after correcting for multiple testing (*P* < 9.62 × 10^−4^), which were the orange and saddle brown modules (Figure S14A). The orange module, which consisted of 167 probes (Table S25), showed a significant negative correlation with CSF Aβ42 levels (*r *= −0.13, *P* = 5.99 × 10^−5^), Aβ40 levels (*r* = −0.12, *P* = 4.07 × 10^−4^), and Aβ38 levels (*r* = −0.13, *P* = 1.22 × 10^−4^). GO analysis highlighted several pathways associated with the splicesome, lipoproteins, and membranes (Figure 4A). PPI analysis for genes annotated to the probes within the orange module identified a number of connected proteins, including ITPR1 and HSP90AA1 (Figure 4B). The saddlebrown module, consisting of 157 probes (Table S26), showed a significant correlation with CSF Aβ42/40 ratio dichotomized after correcting for multiple testing (*r* = −0.11, *P* = 9.12 × 10^−4^), as well as a nominally significant correlation with several other amyloid measures (abnormal Aβ42 levels: *r* = −0.072, *P* = 0.033; Aβ Z‐score: *r *= 0.073, *P* = 0.029; Aβ42/40 ratio: *r* = 0.095, *P* = 4.51 × 10^−3^; amyloid status: *r* = −0.08, *P* = 0.017), in addition to abnormal t‐tau (*r *= −0.072, *P* = 0.032) and YKL‐40 (*r* = −0.081, *P* = 0.016) levels. This module was enriched for developmental pathways, with four false discovery rate (FDR)–significant terms (Figure 4C). PPI analysis highlighted a number of highly connected proteins in the module, most notably NCAM1, which interacts with 14 other proteins within this module (Figure 4D).

## 4 DISCUSSION

To our knowledge, our study represents the largest and most comprehensive EWAS of CSF biomarker levels of amyloid, tau, neurodegeneration, and neuroinflammation to date, investigating 15 different CSF measures in 885 individuals. The most significant DMPs we identified were in the YKL‐40 analysis, where we identified a region of differential methylation spanning ~1 kb in the *CHI3L1* gene close to the TSS, which encodes the YKL‐40 protein. YKL‐40 constitutes a glycoprotein that is secreted by activated macrophages, chondrocytes, neutrophils, and synovial cells, and is thought to play a role in the process of inflammation and tissue remodeling. In addition to being a CSF biomarker of neuroinflammation, YKL‐40 protein concentration has been shown to be significantly increased in the plasma of early AD patients, and positively correlated with neuropsychological test scores.^58^ Similarly, in Parkinson's disease (PD), YKL‐40 protein levels in peripheral blood mononuclar cells (PBMCs) have been shown to be three‐fold higher than in healthy controls, and this was correlated with alterations in mitochondrial function.^59^ *YKL‐40* gene expression has also been reported to be increased in the cerebellum of women with AD when compared to men with AD.^60^ A previous GWAS in the same cohort had already highlighted genetic variation at this locus, altering protein expression in *cis*,^46^ and DNA methylation in this region itself appears to be largely determined by genetic factors, as indicated by our co‐localization analysis with these GWAS data previously generated in the same samples.^46^ We provide evidence that DNA methylation in *CH13L1* (*YKL‐40*) may mediate this protein quantitative trait loci (pQTL: genetic variation affecting protein expression), thereby altering CSF YKL‐40 protein levels. One caveat of this approach was that we assessed DNA methylation in blood, whereas YKL‐40 protein levels were assessed in the CSF. Although proteins in the CSF do originate from the brain, it has been reported that ~80% of CSF proteins are derived from the blood,^61^ and therefore it is plausible that the DNA methylation alterations in *YKL‐40* that we observed in blood are directly mediating the CSF pQTL. In a subset of our cohort we had access to plasma YKL‐40 protein levels, and we showed a significant correlation between CSF and plasma protein levels. Furthermore, two of the Bonferroni‐significant DMPs in *YKL‐40* also showed a correlation with plasma protein levels. Given that Schilde et al. showed that YKL‐40 protein levels are altered in PBMCs, it is plausible that CSF YKL‐40 protein levels are reflecting changes in blood cells, rather than brain cells, and this could represent a downstream peripheral consequence of neuroinflammation. It would be of interest, therefore, to investigate *YKL‐40* DNA methylation and protein expression in the brain in relation to genetic variation to assess whether the alterations we observed in the CSF likely reflect changes in the brain or in the blood.

In addition to the robust associations identified in the CSF YKL‐40 analysis, we also identified seven Bonferroni‐significant DMPs in the CSF NFL analysis, annotated to *OSBPL5, TRIM10, PTGFRN, METRNL, TUBGCP2*, and an intergenic probe. *TRIM10* is a gene that has been shown previously to be hypermethylated in both the brain and blood of patients with PD compared to controls,^62^ which is interesting as serum NfL is associated with cognitive decline in patients with PD.^63^ Of interest, PTGFRN protein levels have previously shown to be altered in CSF‐derived extracellular vesicles in patients with AD relative to MCI.^64^

A recent study had reported several differentially methylated loci in blood that were associated with CSF Aβ42, p‐tau181, and t‐tau levels in 202 individuals in the Alzheimer's Disease Neuroimaging Initiative (ADNI) cohort.^38^ The authors stratified their analysis, performing separate EWAS in the AD and control subjects, and identified four FDR‐significant loci in their p‐tau181 analysis (all in the AD group), four in the t‐tau analysis (three in the AD group, one in the control group), and 112 loci in the Aβ42 analysis (all in the AD group). However, none of these FDR‐significant loci featured in the 1000 most significant loci we identified in any of our EWASs. It is worth noting, however, that for our corresponding analyses (e.g., Aβ42, p‐tau Z‐score, t‐tau Z‐score) we did not identify any significant loci after multiple‐testing (Bonferroni) correction. Similarly, another key difference between our studies includes the stratification by disease status (*N* = 123 control, *N* = 79 AD), and the absence of MCI subjects in the ADNI analyses, which may explain our lack of replication of their findings.

In total, significant DMRs were identified in 12 of the 15 analyses, with eight overlapping regions featuring in multiple CSF measures analyses. A DMR in *MX2* (MX dynamin‐like GTPase 2) was identified in four of the amyloid analyses (abnormal Aβ42, Aβ42, Aβ Z‐score, amyloid status) and is an interferon‐regulated gene that has been shown previously in microglia to be responsive to Aβ.^65^ A DMR in *TGFBI* (transforming growth factor beta‐induced) featured in the Aβ40 and Aβ38 analyses, which is interesting because the protein has been previously shown to be upregulated in plasma in MCI subjects.^66^ The other two genes that featured DMRs in multiple amyloid analyses resided in *ZFP57* (zinc finger protein 57) (Aβ Z‐score, Aβ42/40 ratio) and *ANKMY1* (ankyrn repeat and MYND domain containing 1) (Aβ42, Aβ40), which to our knowledge have not been robustly associated with AD previously.

We identified several regions that overlapped between the different types of CSF protein biomarker analyses; for example, *ZBTB22* (zinc finger and BTB domain containing 22) in the abnormal p‐tau and abnormal Aβ42 analyses, *S100A13 (*S100 calcium binding protein 13) between the abnormal p‐tau and NfL analyses, *STRA6* (signaling receptor and transporter of retinol) between the Aβ38 and neurogranin analyses, and *SORD* (sorbitol dehydrogenase) between the NfL and neurogranin analyses. Lower expression of S100A13 protein in plasma has been observed in individuals with a high polygenic risk score (PRS) for AD,^67^ whereas serum protein expression has been associated with *APOE* genotype.^68^ One noteworthy DMR that only featured in one DMR analysis was *RGMA* (repulsive guidance molecule BMP co‐receptor A), which was identified in the Aβ42 levels analysis, and is located within 81 bp of two CpG sites that were identified in a large meta‐analysis of DNA methylomic studies in AD post‐mortem cortex.^31^

Having observed that the Bonferroni‐significant DMPs in the *CH13L1* (*YKL‐40*) gene in the YKL‐40 CSF EWAS were driven by genetic variation in *cis*, we were also interested to investigate whether any DMPs in our other EWASs resided in genomic regions identified in the corresponding GWASs undertaken previously in the same cohort. We identified four DMPs across the four tau analyses, 16 across the amyloid analyses, eight in the NfL analysis, and two in the neurogranin analysis. Of particular interest was a CpG in the *APOE gene*, which we identified in the Aβ42/40 ratio analysis alongside a CpG in the nearby *APOC1* gene, which also featured in the Aβ Z‐score analysis. The ε4 allele of the *APOE* gene is the greatest genetic risk factor for sporadic AD,^69^ and a recent EWAS reported this CpG to be significantly differentially methylated in blood in non‐demented *APOE* ε4 carriers compared to ε2.^70^ In the future it will be of interest to explore whether the presence of an *APOE* ε4, or indeed ε2 allele, affects methylation across the entire gene.

We used WGCNA to identify modules of co‐methylated loci that were associated significantly with CSF biomarker levels, identifying two modules that passed the Bonferroni significance threshold. The first module (orange), which was correlated with CSF Aβ42 levels, Aβ40 levels, and Aβ38 levels, was enriched for lipoprotein and plasma membrane pathways, with the hub gene *HSP90AA1* having been previously shown to be differentially expressed in MCI and AD blood, and is correlated with the levels of various immune cells, whereas in the brain, its expression levels are correlated with α‐ and β‐secretase activity.^71^ The second module (saddlebrown), which was Bonferroni significantly correlated with Aβ42/40 ratio dichotomized, as well as being nominally significantly correlated with several amyloid measures (abnormal Aβ42 levels, Aβ42/40 ratio, amyloid status), abnormal t‐tau, and YKL‐40 levels was enriched for developmental pathways. Of interest, NCAM1, which was the most connected module from the PPI analysis, has been previously shown to be differentially expressed in the CSF of patients with AD.^72^

Blood is a heterogeneous fluid and we have reported previously that AD is associated with small changes in blood cell proportions.^73^ Therefore, one limitation of our study is that we have used whole blood for DNA methylomic profiling. Although we have attempted to control for this in our analyses by including cell proportions as covariates, future studies performed on DNA isolated from specific cell types would allow the identification of cell type–specific signatures associated with these biomarkers. A second potential limitation is that our study uses a multi‐center design, utilizing whole blood samples collected previously as part of existing large AD biomarker studies from different clinics. However, by adjusting for a sample origin variable (“center”) as a potential confounder on the level of recruitment in our analyses we likely minimized the effect of this heterogeneity in our data. More importantly, all laboratory experiments for DNA methylation profiling on the EPIC array were performed in one batch, which should minimize technical variation as a source of bias. Still, it will be important to replicate all DMPs and DMRs identified in our study in samples from other, ideally more homogenous, populations. It is worth noting that our study was undertaken predominantly in individuals of European ancestry; however, it is important that future EWASs are undertaken in more ethnically diverse populations or in different ethnic groups. Third, with the exception of the YKL‐40 analysis, where we observed DNA methylation alterations of up to 17% (cg17014757), the vast majority of differentially methylated loci we identified in our DMP and DMR analyses for the different CSF biomarkers were relatively modest. Although these individual CpGs are, therefore, unlikely to have utility as single biomarkers, there is the possibility that these could be combined into panels, or combined with other omic data modalities as multimodal markers. In addition, as discussed previously, cell type–specific DNA methylation profiling will be important in the future as these will likely yield larger effect sizes (ESs). Fourth, aside from the *YKL‐40* analysis, the majority of our analyses do not allow any direct inference on potential downstream (or upstream) mechanisms, for example, on gene or protein expression and how differential expression may lead to the observed impact on the AD‐relevant CSF outcome variables analyzed here. Furthermore, even though our CIT provided evidence that *YKL‐40* DNA methylation mediates the pQTL for CSF YKL‐40 protein levels, it is worth noting that the mQTL we identified was in the blood, whereas the pQTL is within the CSF. Therefore, future studies should aim to explore the relationship between YKL‐40 genetic variation, DNA methylation, and protein levels within the same tissue. Finally, EWASs in general do not allow one to distinguish cause–effect relationships, meaning that all observed associations may reflect changes in DNA methylation either preceding (and perhaps partially causing) variation in biomarker levels or they may reflect (at least partially) a direct consequence of the disease process. Future studies integrating different “omic” data sets and/or employing targeted molecular experiments using in vitro or in vivo models will allow a more informed functional interpretation of nominated loci. Notwithstanding, we have undertaken a large and comprehensive study of DNA methylation with respect to various CSF biomarker levels and identified a number of DMPs and DMRs of interest. Most notably we have demonstrated that DNA methylation in the *CH13L1* (*YKL‐40*) gene may mediate its protein expression and suggests the pQTL previously identified for CSF YKL‐40 levels is in part regulated by epigenetic mechanisms. Looking to the future it will be of interest to explore the relationship between *YKL‐40* genetic variation, DNA methylation, and protein expression in the brain.

## CONFLICT OF INTEREST STATEMENT

**R.V**. is principal investigator on institutional clinical trial agreements with Alector, Biogen, Denali, J&J, Lilly, NovoNordisk, and UCB, and has consultancy agreements (as Data Safety Monitoring Board chair) with AC Immune and Novartis. **P.S**. is co‐chair of the EVOKE program for NovoNordisk, and principal investigator on phase 1b studies for UCB and AC Immune, phase 2a for Alzheon, and phase 2b for Toyama. He is a member of the Data Safety Monitoring Board for Immunobrain Checkpoint and is chair of the World Dementia Council. **C.E.T**. is employed by Amsterdam UMC. She has grants or contracts for Research of the European Commission (Marie Curie International Training Network, grant agreement No 860197 (MIRIADE), Innovative Medicines Initiatives 3TR (Horizon 2020, grant no 831434) EPND (IMI 2 Joint Undertaking (JU), grant No. 101034344) and JPND (bPRIDE), National MS Society (Progressive MS alliance), Alzheimer's Drug Discovery Foundation, Alzheimer's Association, Health Holland, the Dutch Research Council (ZonMW), including TAP‐dementia, a ZonMw funded project (#10510032120003) in the context of the Dutch National Dementia Strategy, The Selfridges Group Foundation, Alzheimer Netherlands. She is recipient of ABOARD, which is a public‐private partnership receiving funding from ZonMW (#73305095007) and Health∼Holland, Topsector Life Sciences & Health (PPP‐allowance; #LSHM20106). She is also a contract researcher for ADx Neurosciences, AC‐Immune, Aribio, Axon Neurosciences, Beckman‐Coulter, BioConnect, Bioorchestra, Brainstorm Therapeutics, Celgene, Cognition Therapeutics, EIP Pharma, Eisai, Eli Lilly Fujirebio, Grifols, Instant Nano Biosensors, Merck, Novo Nordisk, Olink, PeopleBio, Quanterix, Roche, Siemens, Toyama, Vivoryon, and the European Commission. She has received payment or honoraria from Roche, Novo Nordisk, and Grifols, where all payments were made to her institution. She also serves on editorial boards of *Medidact Neurologie/Springer*; and in *Neurology: Neuroimmunology & Neuroinflammation*. She is editor of *Alzheimer Research and Therapy*. **G.B.F**. has received funding through the Private Foundation of Geneva University Hospitals from: A.P.R.A.—Association Suisse pour la Recherche sur la Maladie d'Alzheimer, Genève; Fondation Segré, Genève; Ivan Pictet, Genève; Race Against Dementia Foundation, London, UK; Fondation Child Care, Genève; Fondation Edmond J. Safra, Genève; Fondation Minkoff, Genève; Fondazione Agusta, Lugano; McCall Macbain Foundation, Canada; Nicole et René Keller, Genève; Fondation AETAS, Genève. He has received funding through the University of Geneva or Geneva University Hospitals for IISSs from ROCHE Pharmaceuticals, OM Pharma, EISAI Pharmaceuticals, Biogen Pharmaceuticals, and Novo Nordisk; for competitive research projects from H2020, Innovative Medicines Initiative (IMI), IMI2, Swiss National Science Foundation, and VELUX Foundation. He has received consulting fees from Biogen, Diadem, and Roche, where all payments were made to his institution. He has received payment or honoraria for lectures, presentations, speaker's bureaus, manuscript writing, or educational events from Biogen, Roche, Novo Nordisk, and GE HealthCare, which were all paid to his institution. **J.R**. participates in the Discovery Alliance Advisory Group (ADAG) for the ARUK Drug Discovery Institute (DDI), the MND Dementia Research Institute (DRI) and is vice chair of the My Name's Doddie Foundation Research Review Committee, all of which are unpaid. **S.E**. received unrestricted grants from Janssen Pharmaceutica and ADx Neurosciences (paid to the institution) and served as consultant and on advisory boards for Biogen, Eisai, Icometrix, Novartis, Pfizer, and Roche. A.L. participated in advisory boards from Biogen, Eisai, Fujirebio‐Europe, Grifols, Novartis, Roche, Otsuka Pharmaceutical, Nutricia, Zambón, and Novo Nordisk. He is a member of the executive board and co‐chair of the European Alzheimer's Disease Consortium (EADC), co‐chair of the Dementia Panel of the European Academy of Neurology and is vice‐president of the Belgian Dementia Council (BeDeCo). **A.L**. declares a filed patent application (WO2019175379 A1 Markers of synaptopathy in neurodegenerative disease). He has also received consulting fees from Grifols and Lilly. **F.V**. is a member of the Dutch advisory board for Eisai. **M.T**. has received honoraria for presentations to Brain Therapeutics, Roche, and Lavipharm. She has had grants/contracts with Altoida and Novo Nordisk, and has had a fiduciary role for Alzheimer Hellas, the Greek Federation of Alzheimer's Disease, and the National Observatory on Dementia. **K.B**. has served as a consultant and at advisory boards for Abbvie, AriBio, AC Immune, Acumen, ALZPath, BioArctic, Biogen, Eisai, Lilly, Moleac Pte. Ltd, Novartis, Ono Pharma, Prothena, Roche Diagnostics, and Siemens Healthineers; has served on data monitoring committees for Julius Clinical and Novartis; has given lectures, produced educational materials, and participated in educational programs for AC Immune, Biogen, Celdara Medical, Eisai, and Roche Diagnostics; and is a co‐founder of Brain Biomarker Solutions in Gothenburg AB (BBS), which is a part of the GU Ventures Incubator Program, outside the work presented in this paper. **H.Z**. has served at scientific advisory boards and/or as a consultant for Abbvie, Acumen, Alector, Alzinova, ALZPath, Annexon, Apellis, Artery Therapeutics, AZTherapies, Cognito Therapeutics, CogRx, Denali, Eisai, Merry Life, Nervgen, Novo Nordisk, Optoceutics, Passage Bio, Pinteon Therapeutics, Prothena, Red Abbey Labs, reMYND, Roche, Samumed, Siemens Healthineers, Triplet Therapeutics, and Wave; has given lectures in symposia sponsored by Alzecure, Biogen, Cellectricon, Fujirebio, Lilly, and Roche; and is a co‐founder of Brain Biomarker Solutions in Gothenburg AB (BBS), which is a part of the GU Ventures Incubator Program (outside submitted work). He is chair of the Alzheimer's Association Global Biomarker Standardization Consortium. **P.J.V**. has received honoraria (paid to institution) by Stiftung Synapsis, Alzheimer Forschung Schweiz AFS, is a member of the executive board for EADC, and has a patent on AD subtypes (PCT/NL2020/050216). The other authors (**R.G.S**., **E.P**., **M.K**., **J.I**., **V.D**., **P.J**., **M.W**., **A.F**., **J.S**., **Y.F.‐L**., **L.F**., **O.B**., **B.R**., **E.D.R**., **P.M.‐L**., **M.A**., **M.T**., **I.S**., **J.P**., **G.P**., **L.W**., **A.N.‐H**., **J.S**., **S.J.B.V**., **S.L**., **L.B**., **K.L**.) declare that they have nothing to disclose. Author disclosures are available in the Supporting Information.

## CONSENT STATEMENT

All human subjects provided informed consent.

## Supporting information

Supporting Information

Supporting Information

Supporting Information
