# Supplementary material for: Blood DNA methylomic signatures associated with CSF biomarkers of Alzheimer's disease in the EMIF‐AD study
Source: Alzheimers Dement. 2024 Aug 28;20(10):6722–39. doi: 10.1002/alz.14098 (PMC11485320; doi:10.1002/alz.14098)

**Supplementary Figure 1: A heatmap highlighting the correlation of various tau, amyloid and other CSF AD-relevant biomarkers**. Shown is the Pearson’s correlation coefficient (*r*) between diagnosis (disease status), four tau measures (p-tau assay Z-score, abnormal p-tau, t-tau assay z-score, abnormal t-tau), eight amyloid measures (abnormal Aβ42, Aβ42, Aβ40, Aβ38, Aβ Z-score, Aβ42/40 ratio, Aβ42/40 ratio dichotomized, amyloid status) and three other biomarkers of neuroinflammation (YKL-40), neurodegeneration (NFL) and synaptic dysfunction (neurogranin). Blue represents a positive correlation, whilst red indicates a negative correlation.


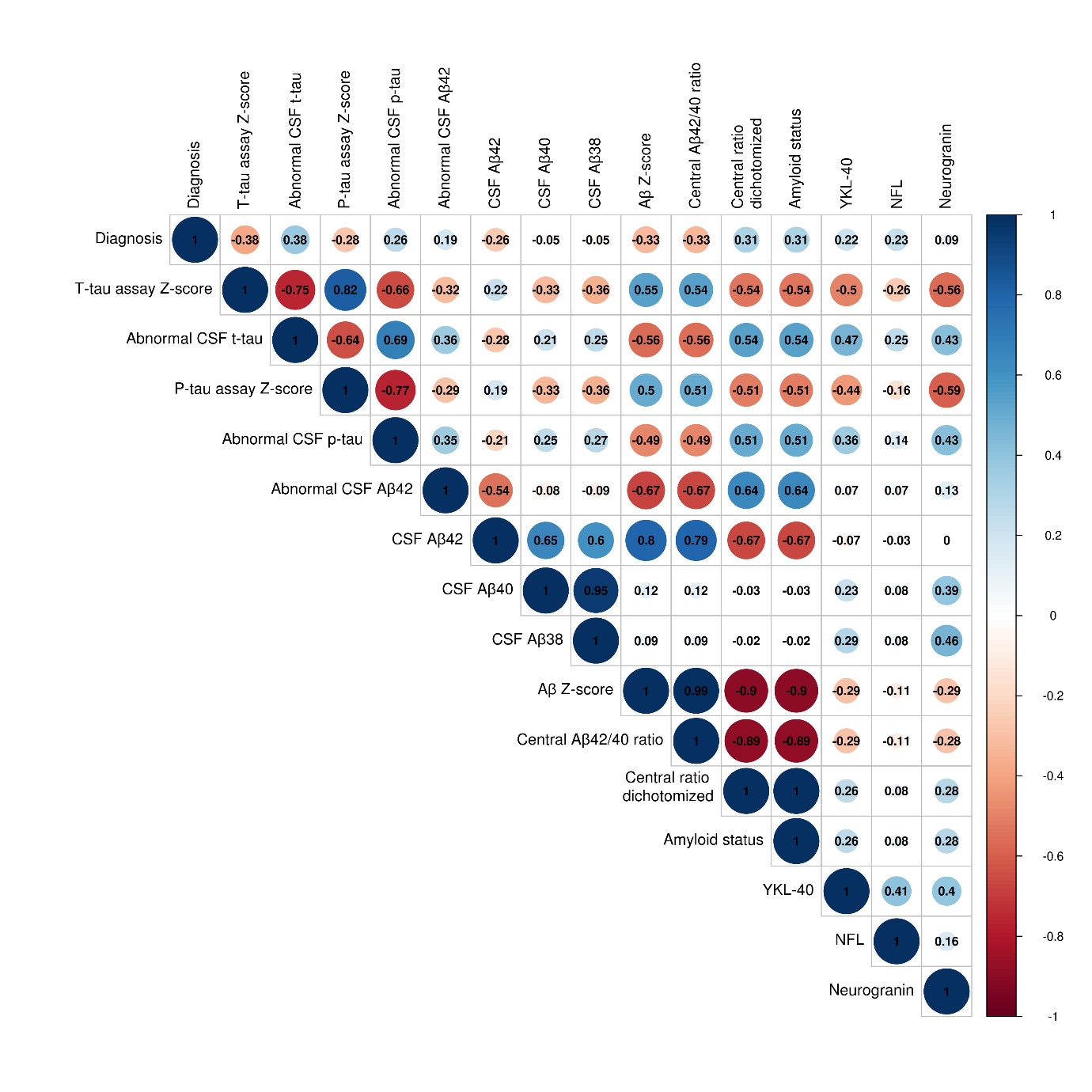


**Supplementary Figure 2: Differentially methylated regions (DMRs) associated with CSF p-tau Z-score**. We identified three DMRs, including (**A**) four probes in the *LINC00857* gene (chr10:81967195-81967284), (**B**) three probes in the *C3* gene (chr19:6721855-6721965) and (**C**) eight probes in the *CMYA5* gene (chr5:78985425-78985592). The X-axis shows genomic position, whilst the Y-axis shows -log10(p). Red probes (circles) represent a positive effect size (ES) ≥ 1%, green probes (circles) represent a negative ES ≥ 1% and black probes (circles) represent an ES smaller than 1%. Filled circles denote the probes in the DMR. ES is defined as the % methylation difference across the range of values. The gene tracks are shown in black underneath, with CpG islands in green. Full details on the DMRs can be found in Supplementary Table 6A.

**A**

**
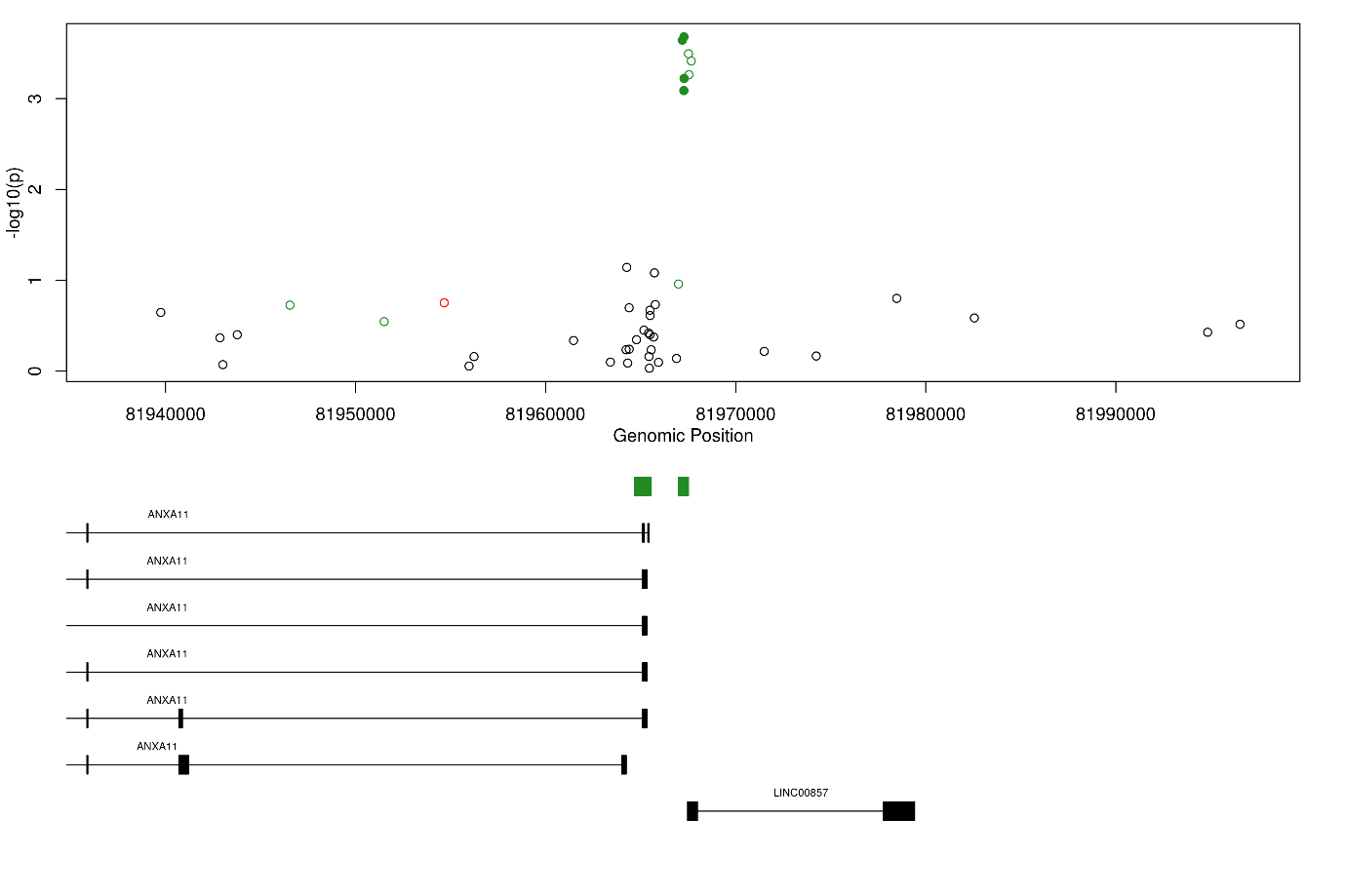
**

**Supplementary Figure 2 cont.**

**B**


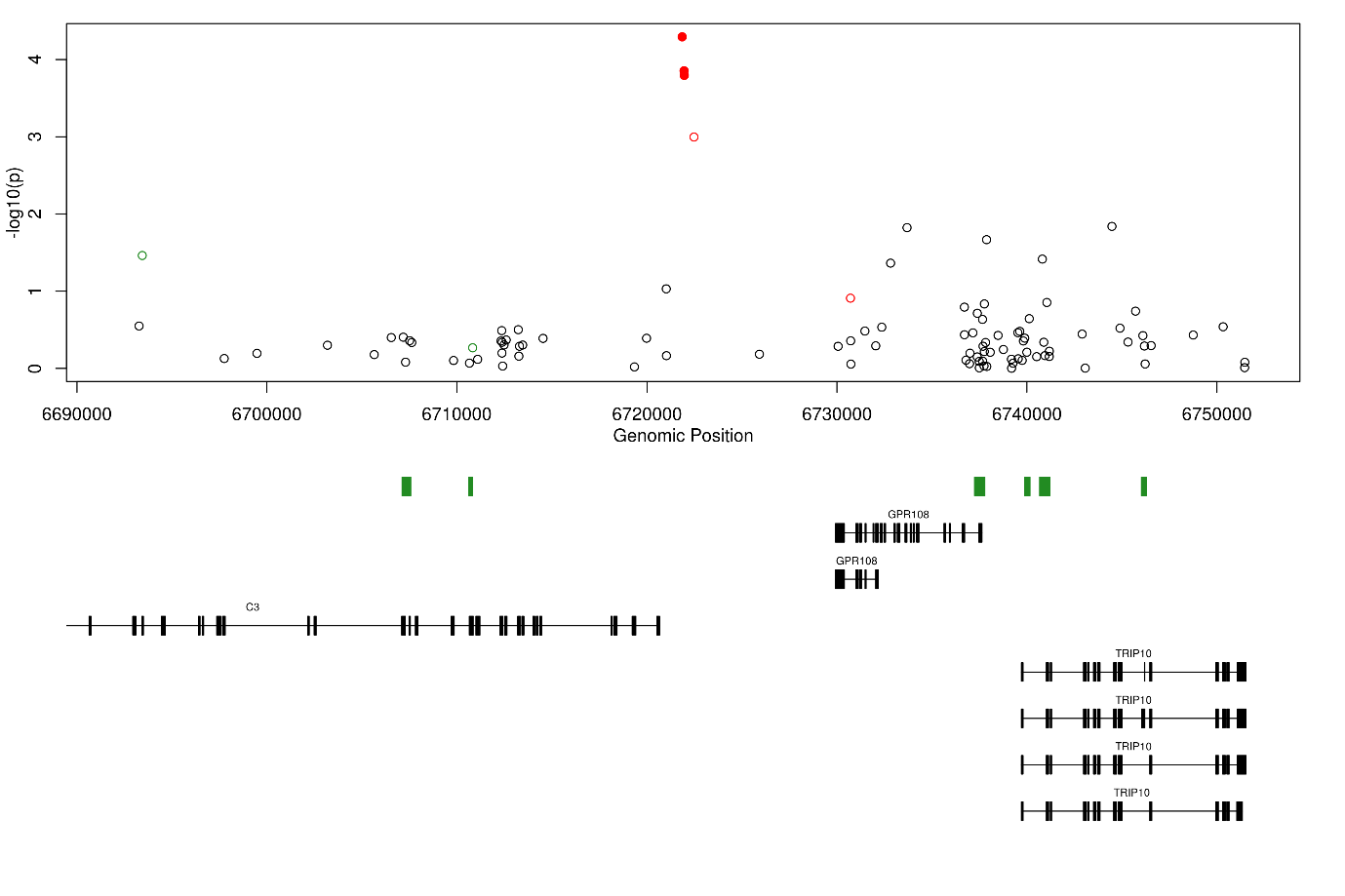


**C**


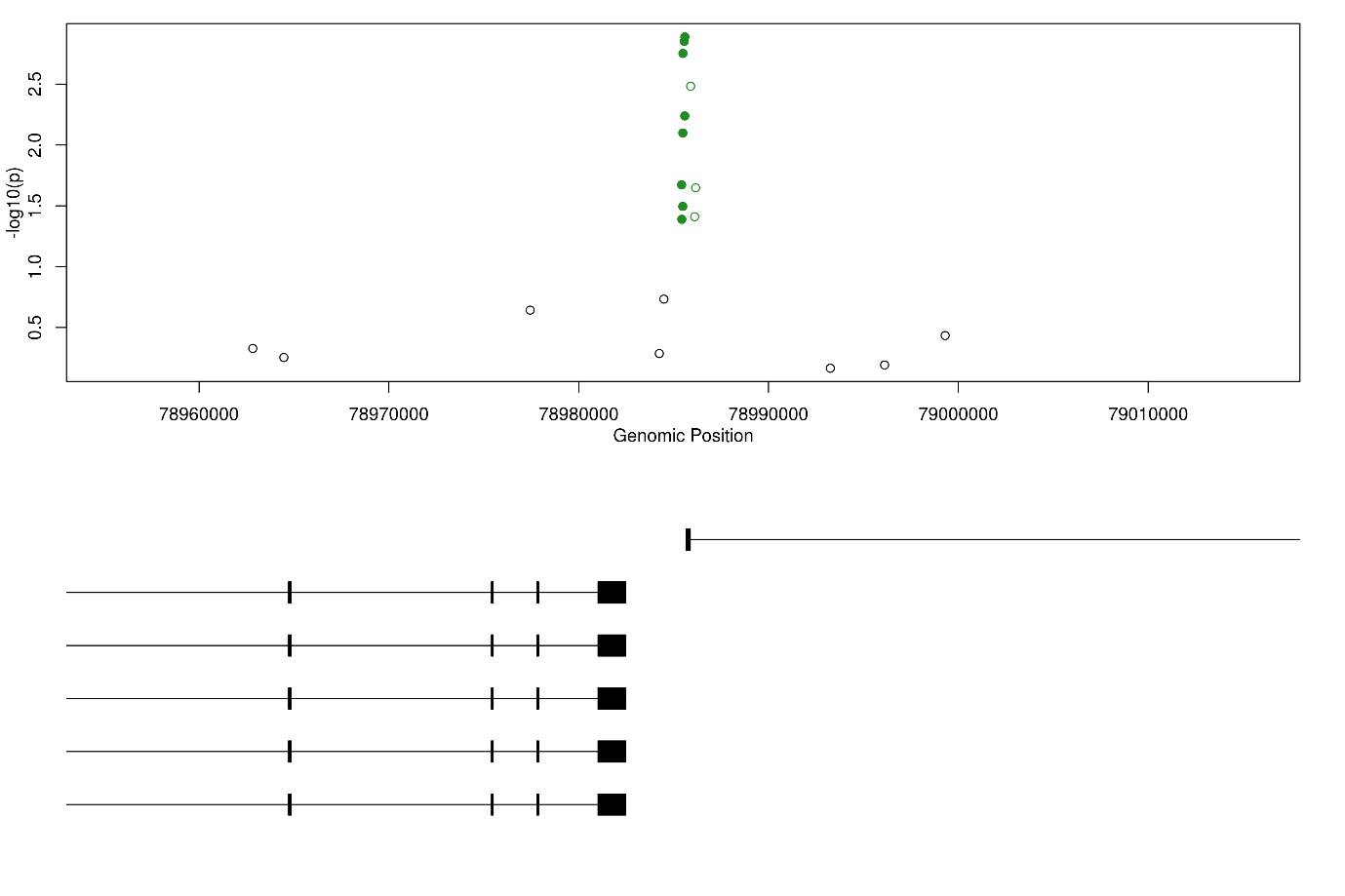


**Supplementary Figure 3: Differentially methylated regions (DMRs) associated with CSF abnormal p-tau**. We identified three DMRs, including (**A**) ten probes in the *S100A13* gene (chr1:153599573-153599831), (**B**) 13 probes in the *ZBTB22* gene (chr6:33282971-33283189) and (**C**) four probes in the *SPATS2* gene (chr12:49783072-49783222). The X-axis shows genomic position, whilst the Y-axis shows -log10(p). Red probes (circles) represent a positive effect size (ES) ≥ 1%, green probes (circles) represent a negative ES ≥ 1% and black probes (circles) represent an ES smaller than 1%. Filled circles denote the probes in the DMR. ES is defined as the % methylation difference across the range of values. The gene tracks are shown in black underneath, with CpG islands in green. Full details on the DMRs can be found in Supplementary Table 6C.

**A**


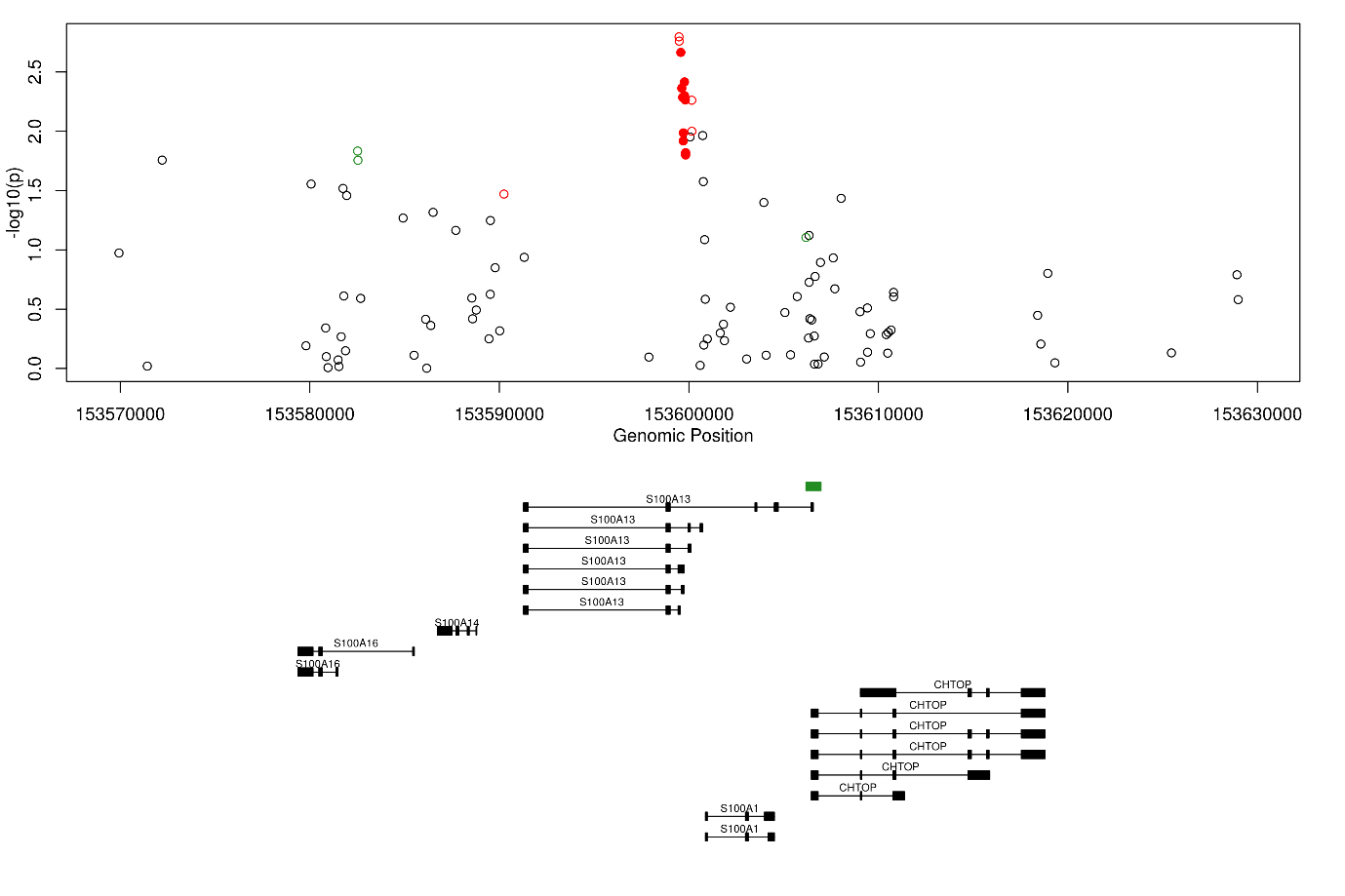


**Supplementary Figure 3 cont.**

**B**


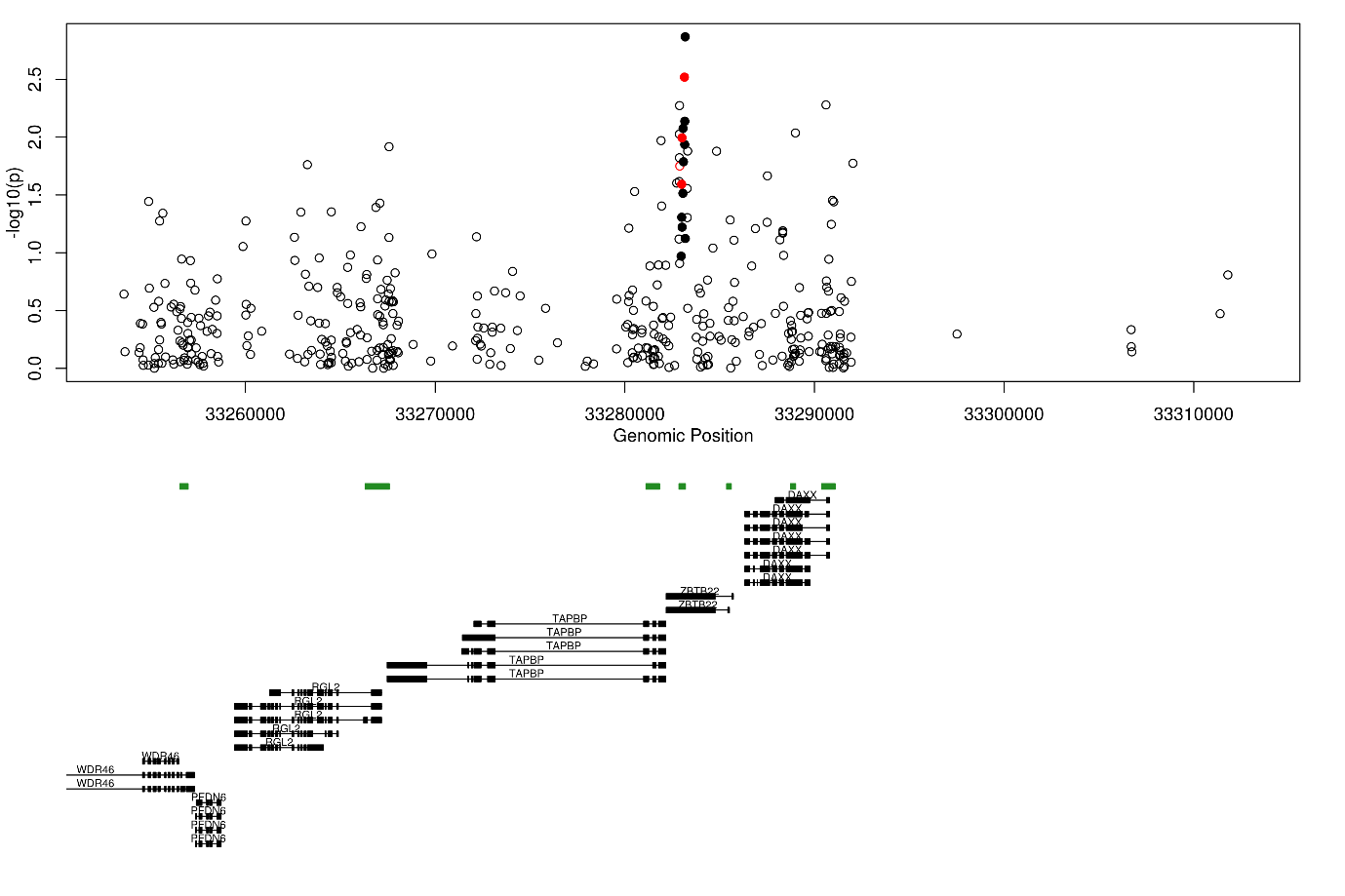


**C**


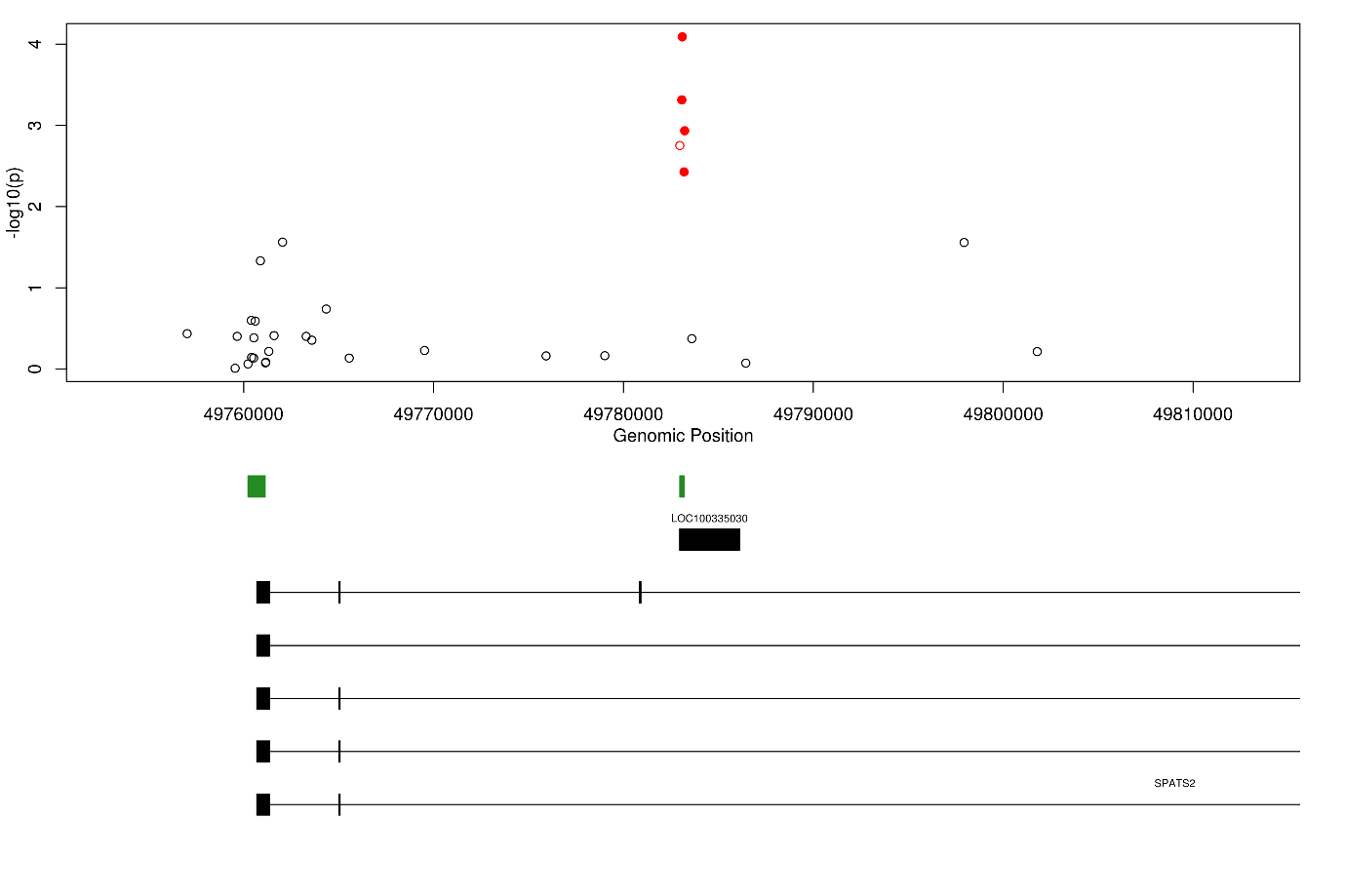


**Supplementary Figure 4: The differentially methylated region (DMR) associated with abnormal CSF Aβ42**. We identified five DMRs including (**A**) five probes in the *MX2* gene (chr21:42741698-42741991), (**B**) five probes in the *ABCG2* gene (chr4:89152511-89152696), (**C**) four probes in the *RHOH* gene (chr4:40192546-40192715), (**D**) 12 probes in the *ZBTB22* gene (chr6:33282856-33283021) and (**E**) four probes in the *SLFN12* gene (chr17:33759929-33759986). The X-axis shows genomic position, whilst the Y-axis shows -log10(p). Red probes (circles) represent a positive effect size (ES) ≥ 1%, green probes (circles) represent a negative ES ≥ 1% and black probes (circles) represent an ES smaller than 1%. Filled circles denote the probes in the DMR. ES is defined as the % methylation difference across the range of values. The gene tracks are shown in black underneath, with CpG islands in green. Full details on the DMRs can be found in Supplementary Table 15A.

**A**

**A**


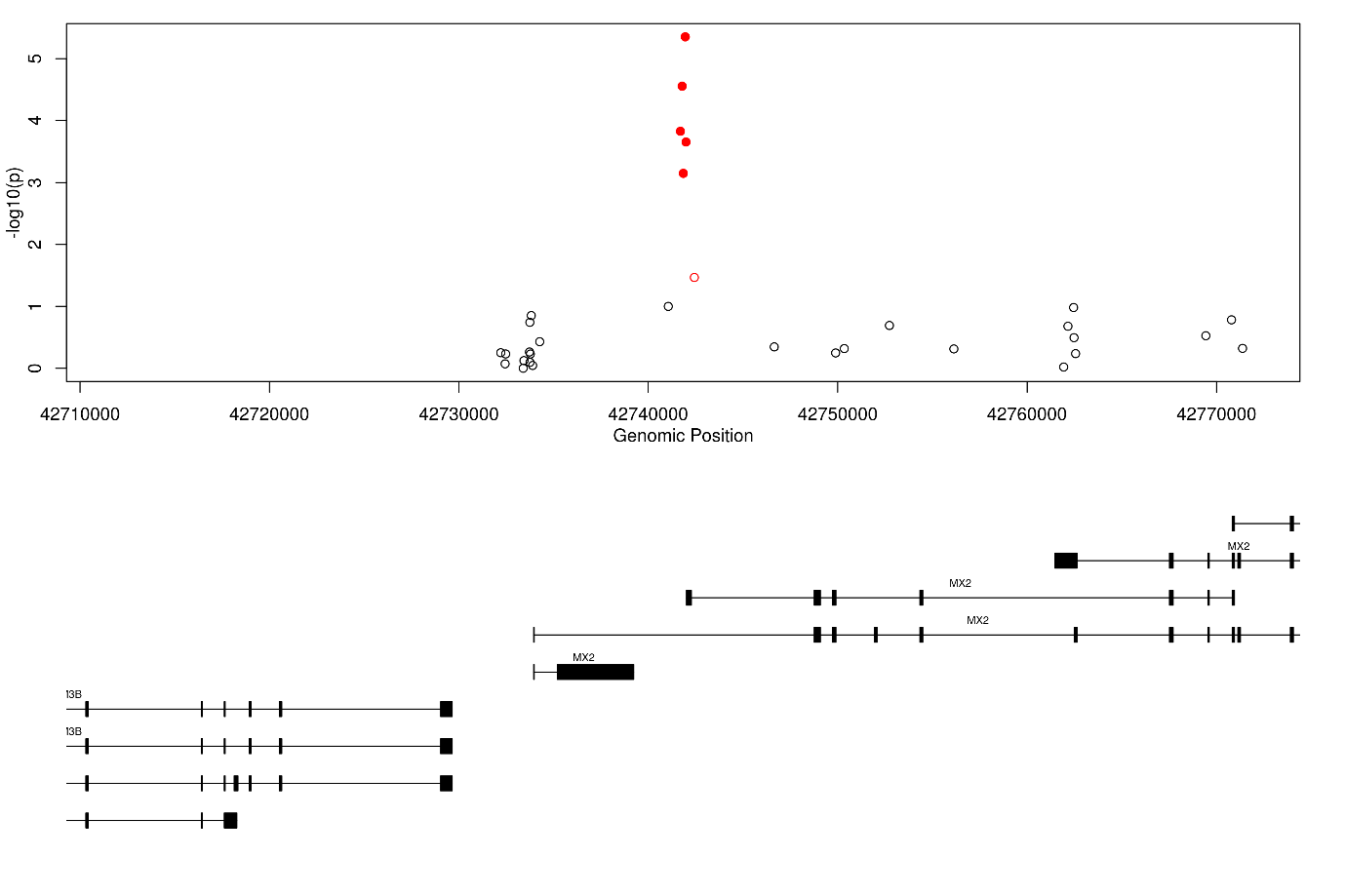


**Supplementary Figure 4 cont.**

**B**

**
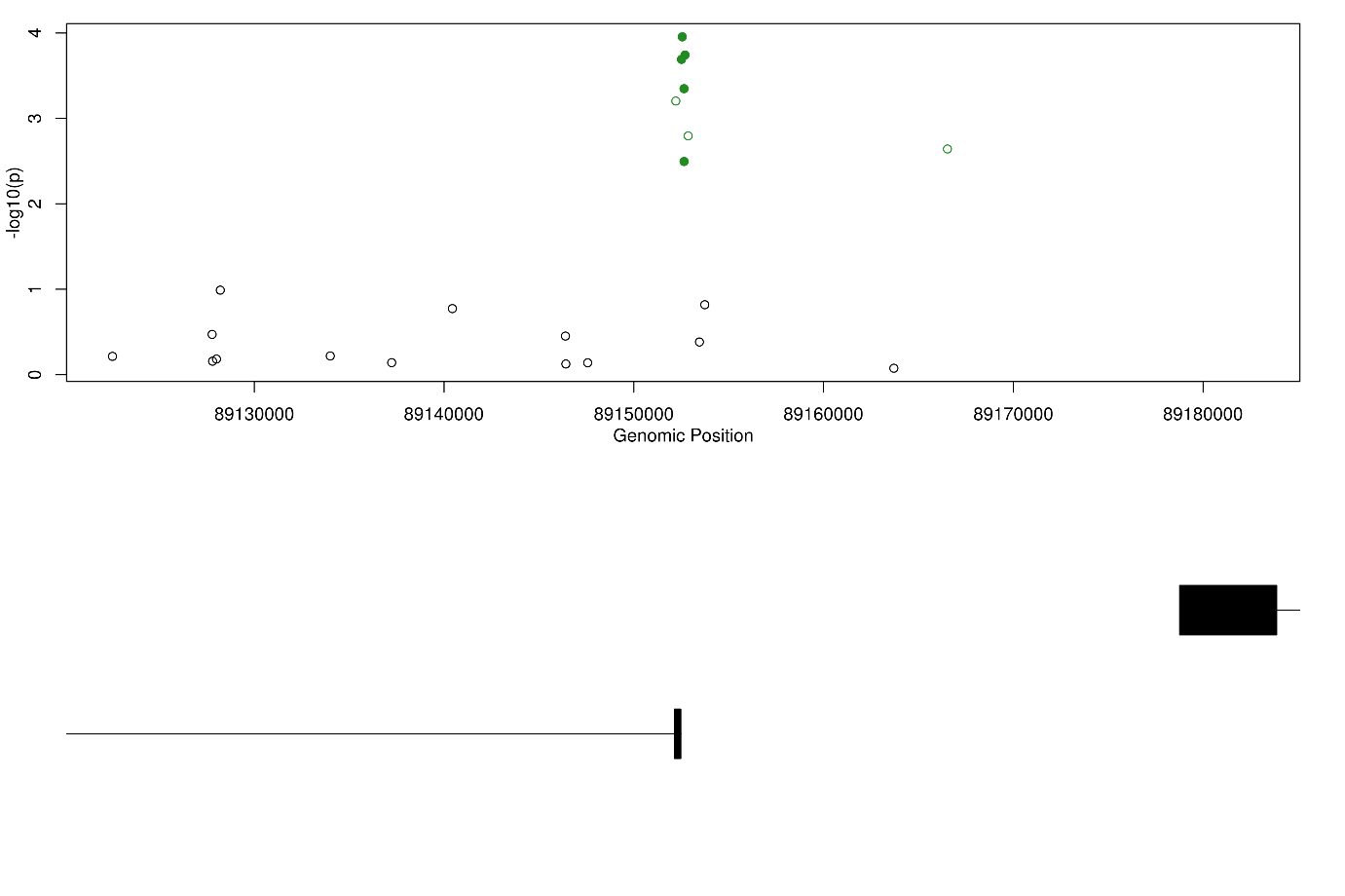
**

**C**


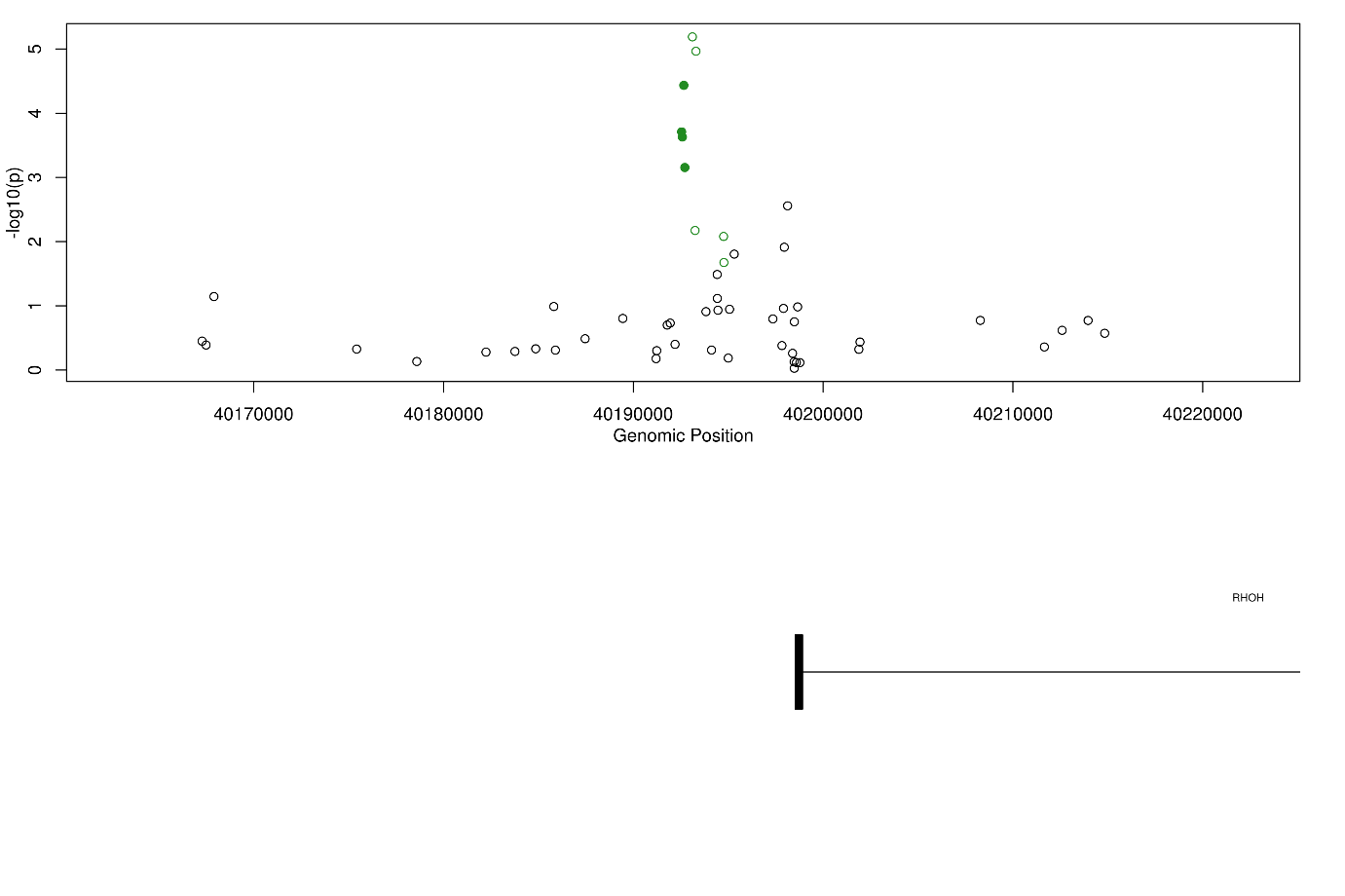


**Supplementary Figure 4 cont.**

**D**


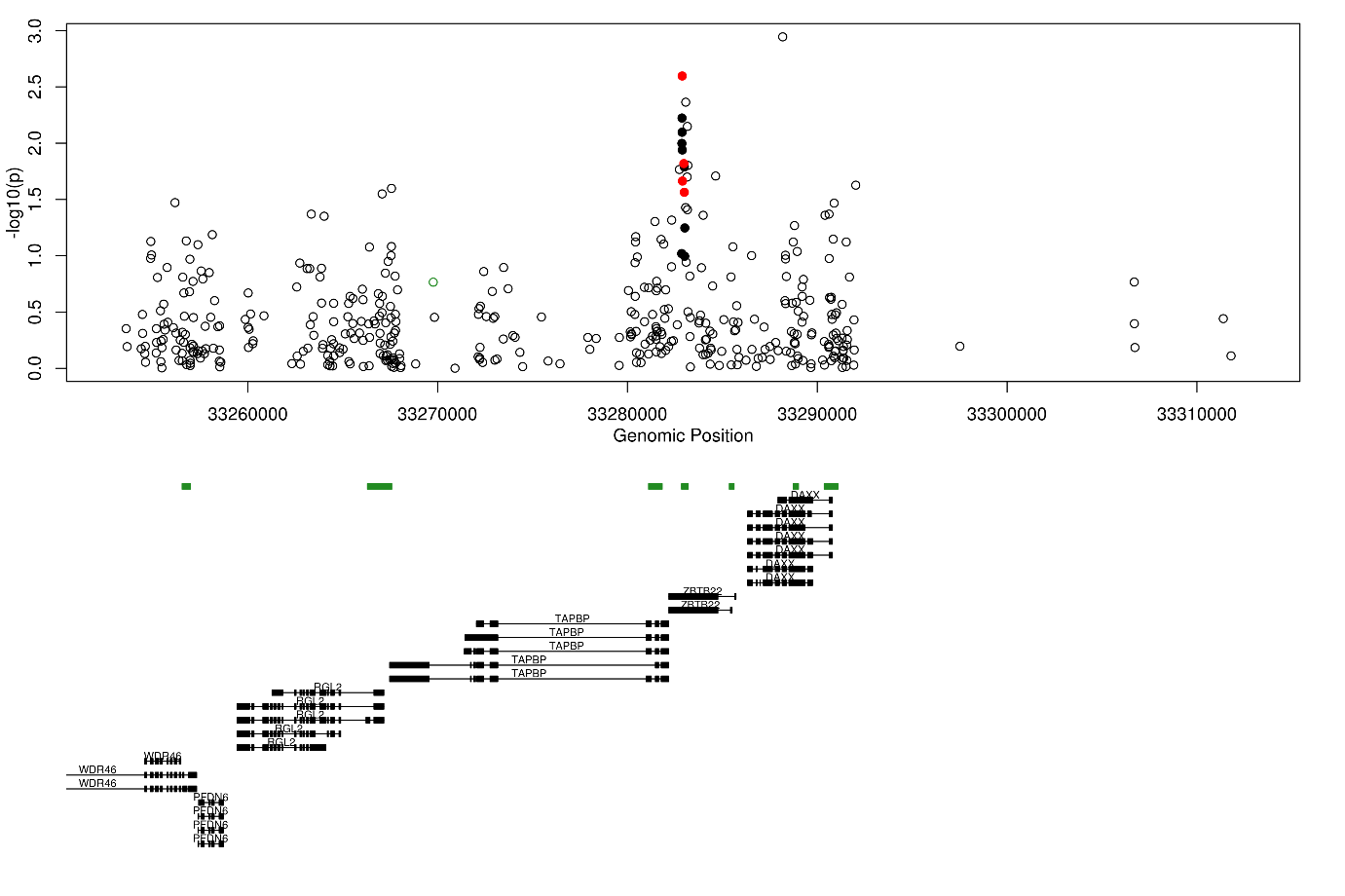


**E**

**
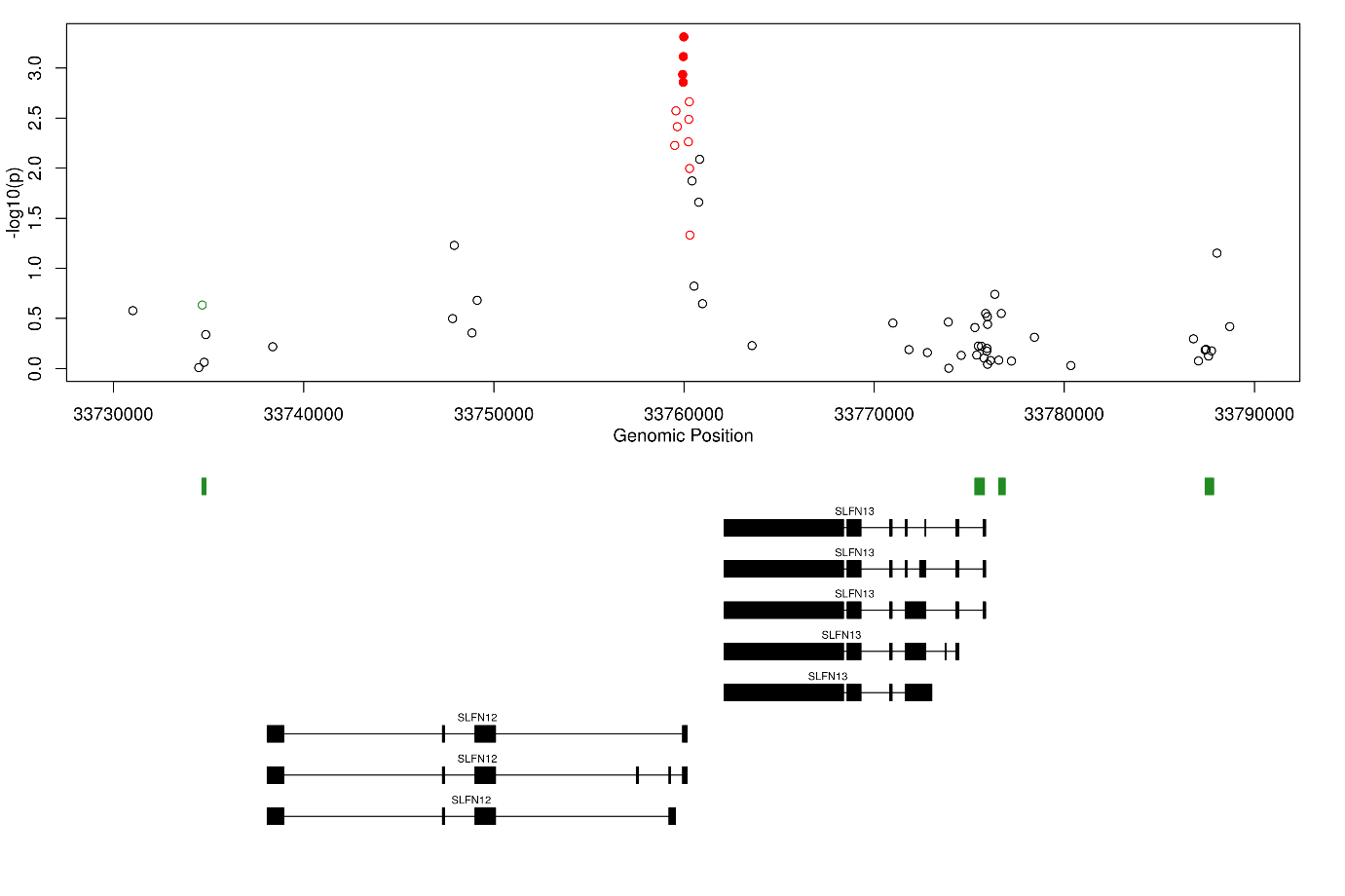
**

**Supplementary Figure 5: Differentially methylated regions (DMRs) associated with CSF Aβ42 levels**. We identified eight DMRs, including (**A**) six probes in the *AKR1E2* gene (chr10:4868328-4868398), (**B**) seven probes in the *ADHFE1* gene (chr8:67344553-67344720), (**C**) five probes in the *ANKMY1* gene (chr2:241497412-241497554), (**D**) seven probes in the *RGMA* gene (chr15:93616943-93617080), (**E**) five probes in the *RBBP7* gene (chrX:16888596-16888633), (**F**) four probes in *LOC101929241* (chr14:97925015-97925164), (**G**) three probes in the *MX2* gene (chr21:42741788-42741952) and (**H**) eight probes in the *VARS2* gene (chr6:30881560-30881664). The X-axis shows genomic position, whilst the Y-axis shows -log10(p). Red probes (circles) represent a positive effect size (ES) ≥ 1%, green probes (circles) represent a negative ES ≥ 1% and black probes (circles) represent an ES smaller than 1%. Filled circles denote the probes in the DMR. ES is defined as the % methylation difference across the range of values. The gene tracks are shown in black underneath, with CpG islands in green. Full details on the DMRs can be found in Supplementary Table 15B.

**A**


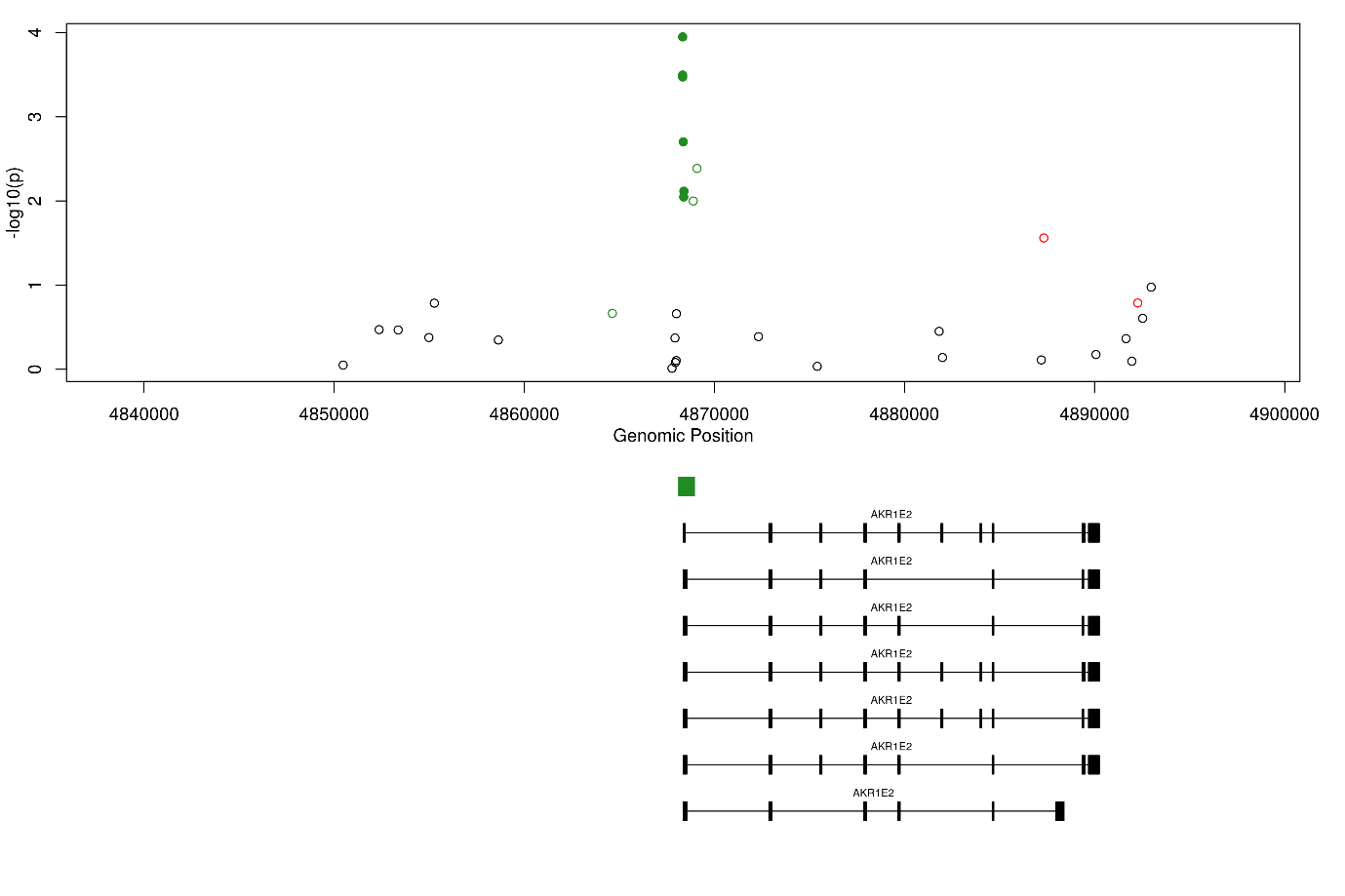


**Supplementary Figure 5 cont.**

**B**


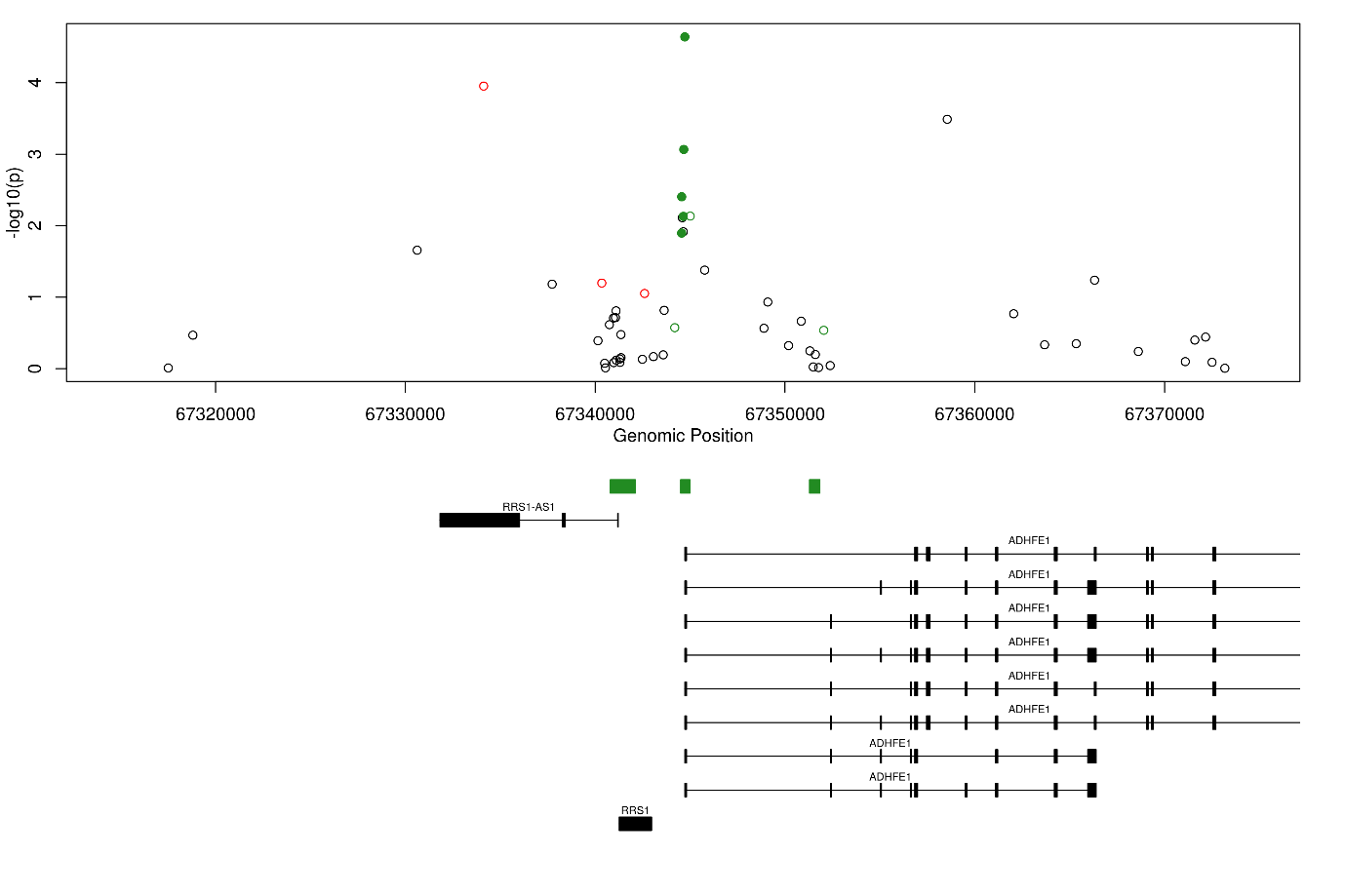


**C**


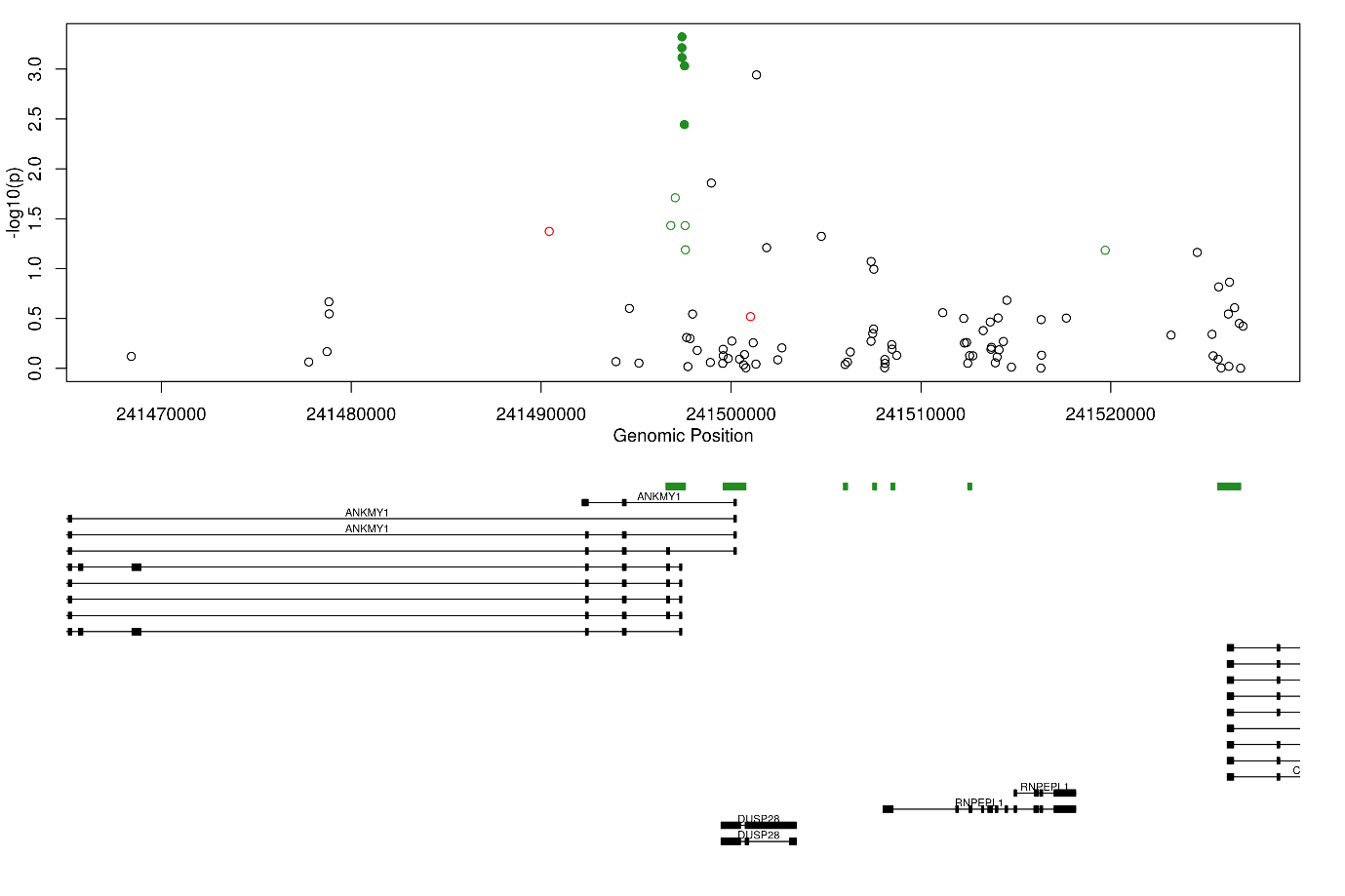


**Supplementary Figure 5 cont.**

**D**


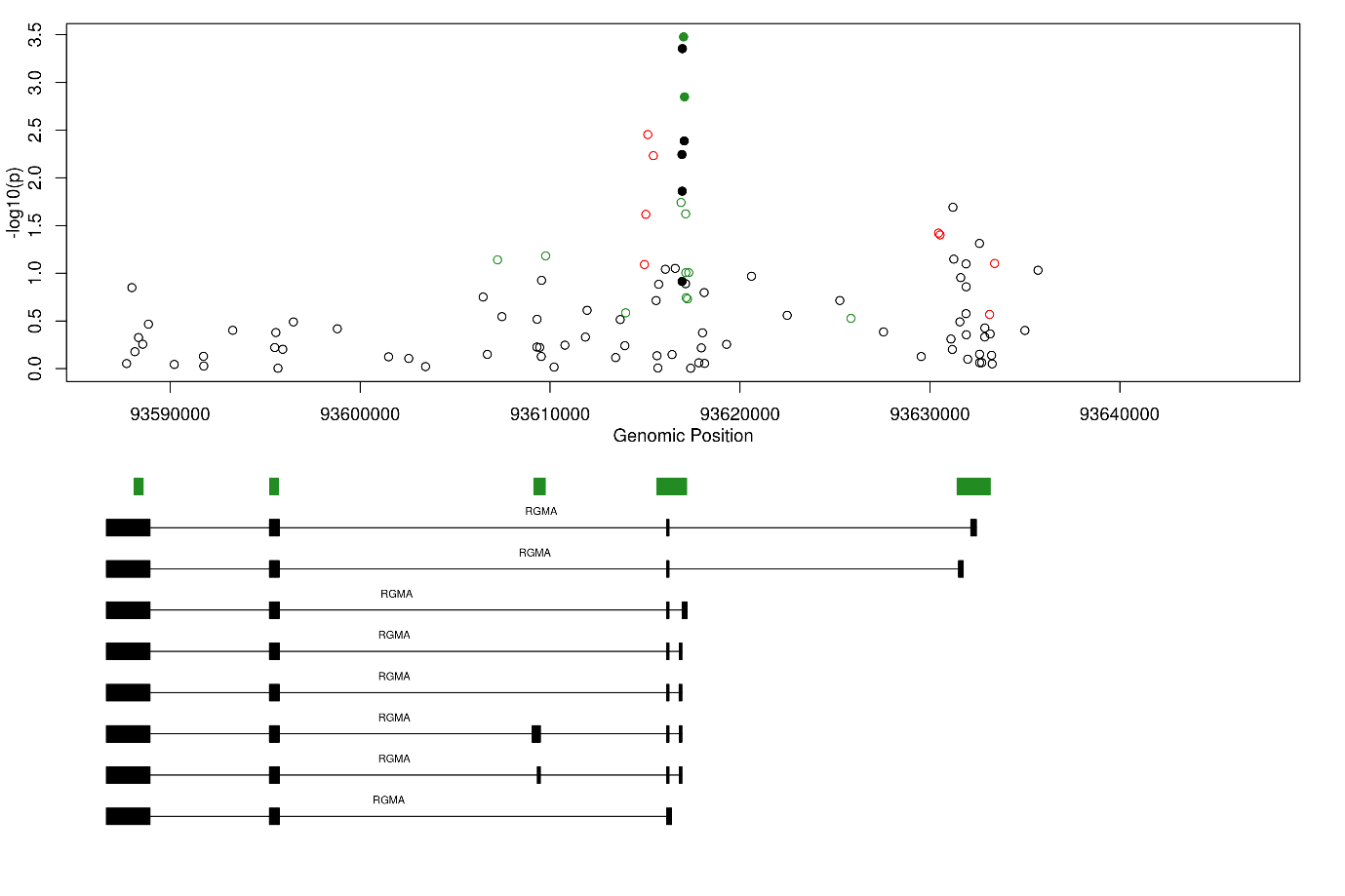


**E**

**
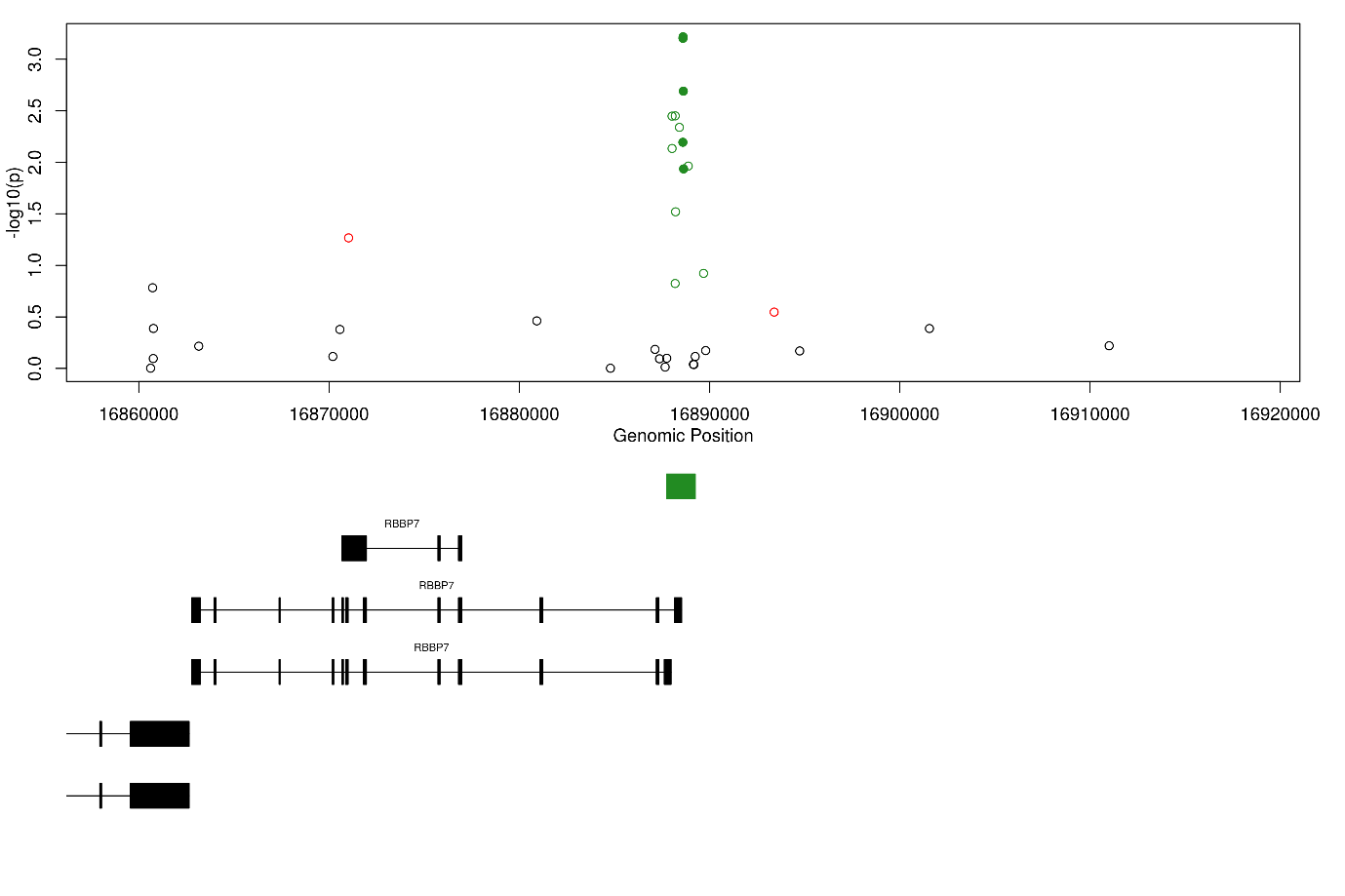
**

**Supplementary Figure 5 cont.**

**F**


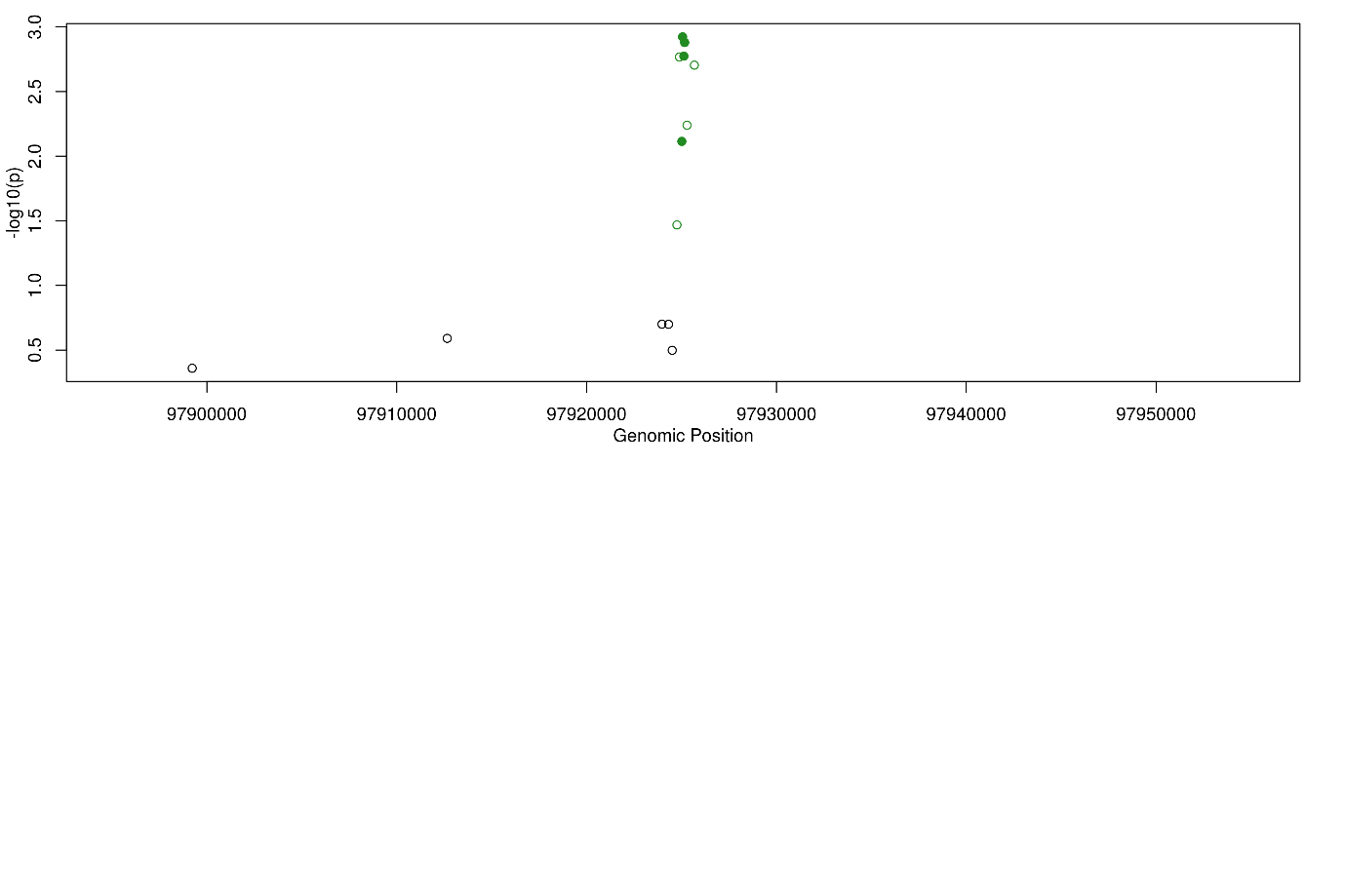


**G**


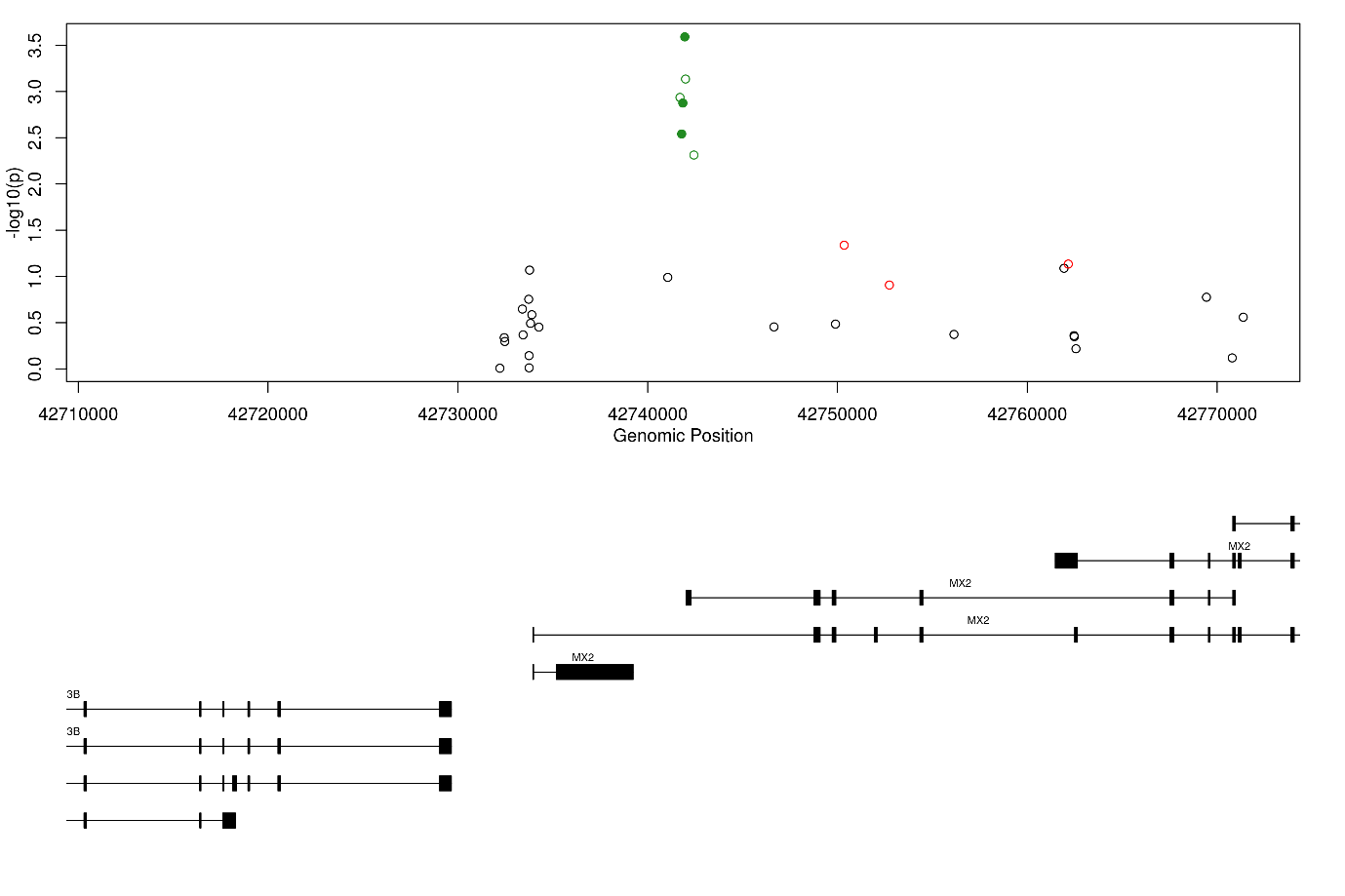


**Supplementary Figure 5 cont.**

**H**


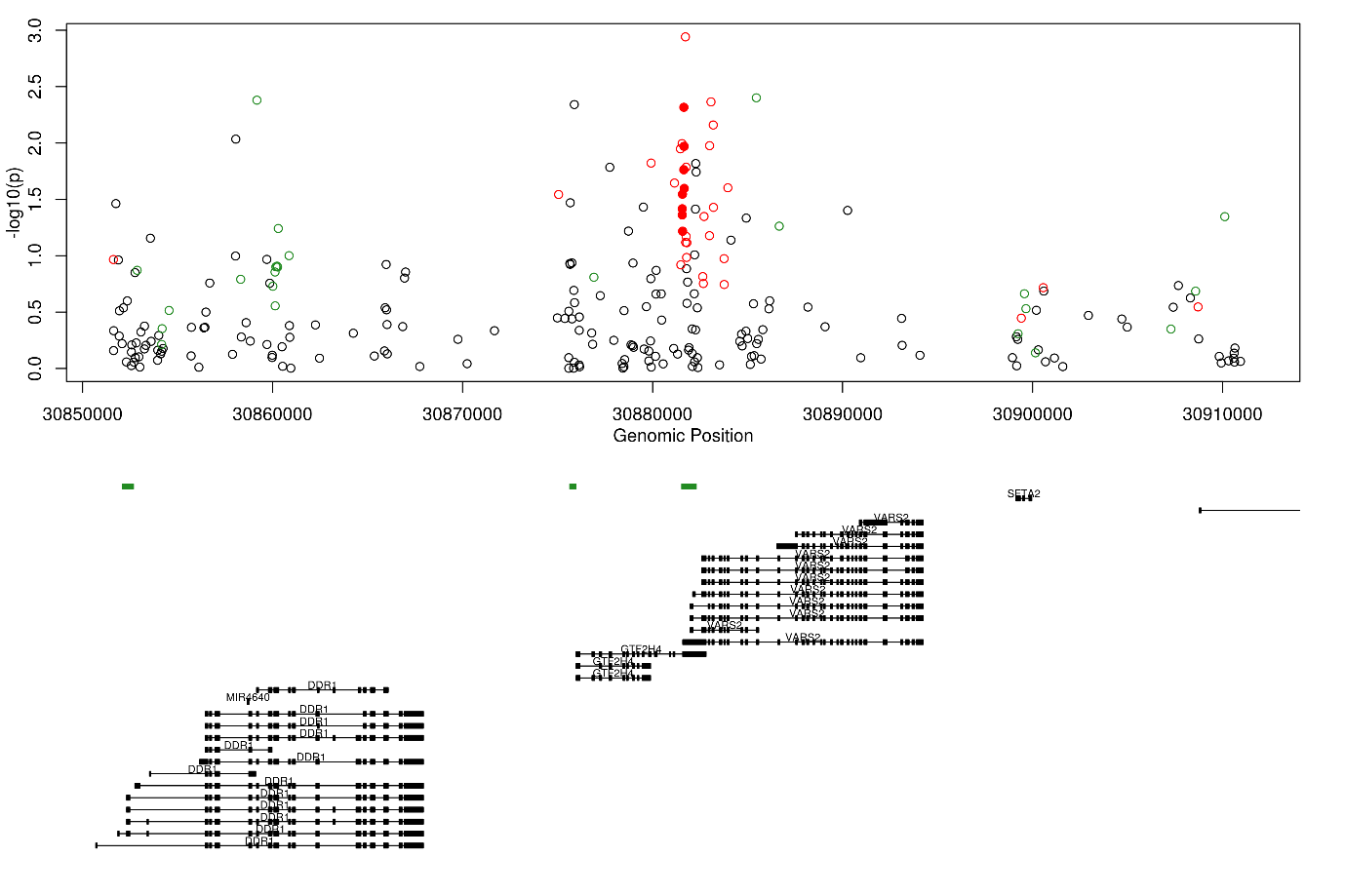


**Supplementary Figure 6: Differentially methylated regions (DMRs) associated with CSF Aβ40 levels**. We identified two DMRs, including (**A**) five probes in the *TGFBI* gene (chr5:135364552-135364580) and (**B**) five probes in the *ANKMY1* gene (chr2:241497412-241497554). The X-axis shows genomic position, whilst the Y-axis shows -log10(p). Red probes (circles) represent a positive effect size (ES) ≥ 1%, green probes (circles) represent a negative ES ≥ 1% and black probes (circles) represent an ES smaller than 1%. Filled circles denote the probes in the DMR. ES is defined as the % methylation difference across the range of values. The gene tracks are shown in black underneath, with CpG islands in green. Full details on the DMRs can be found in Supplementary Table 15C.

**A**


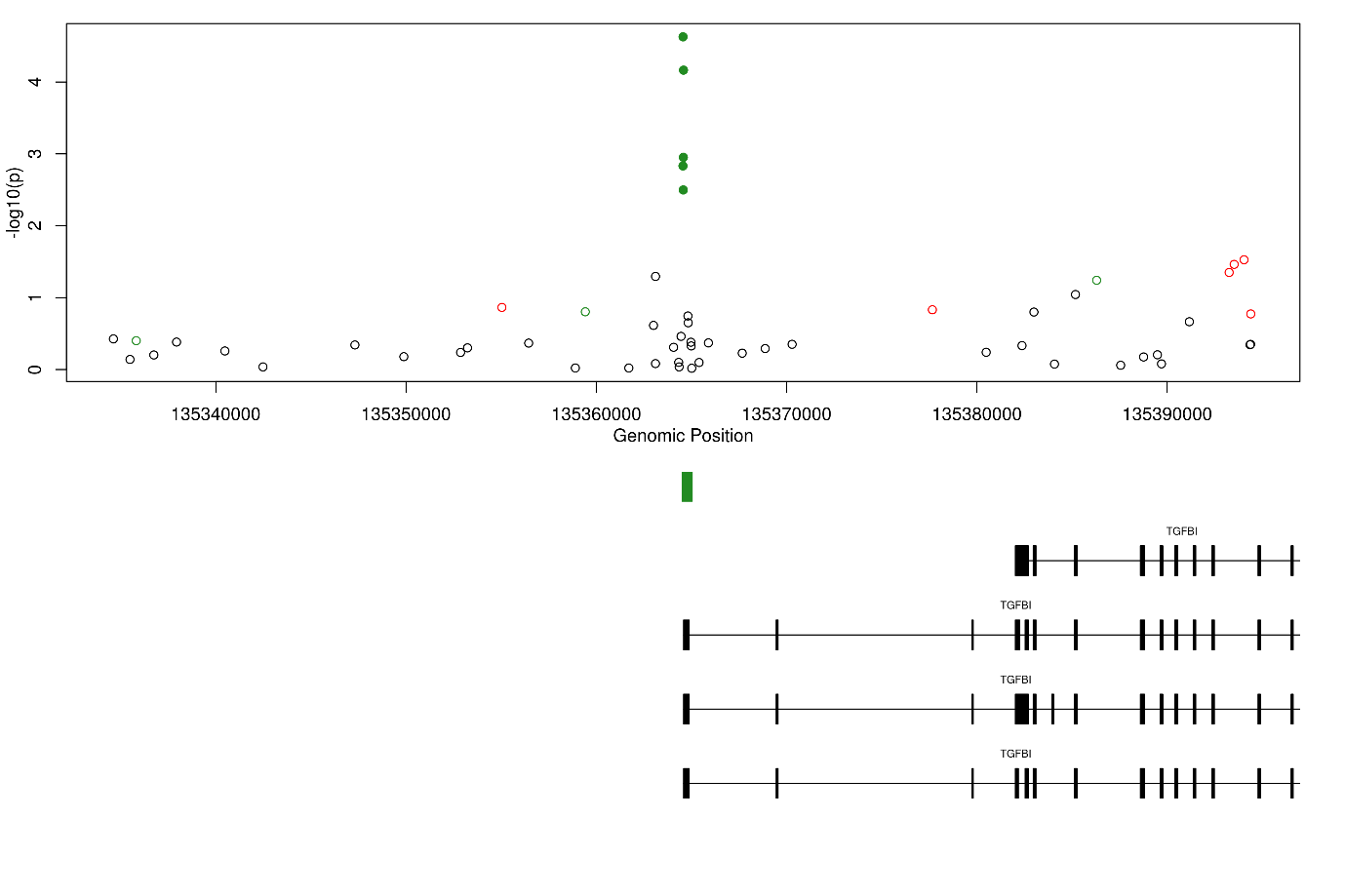


**Supplementary Figure 6 cont.**

**B**


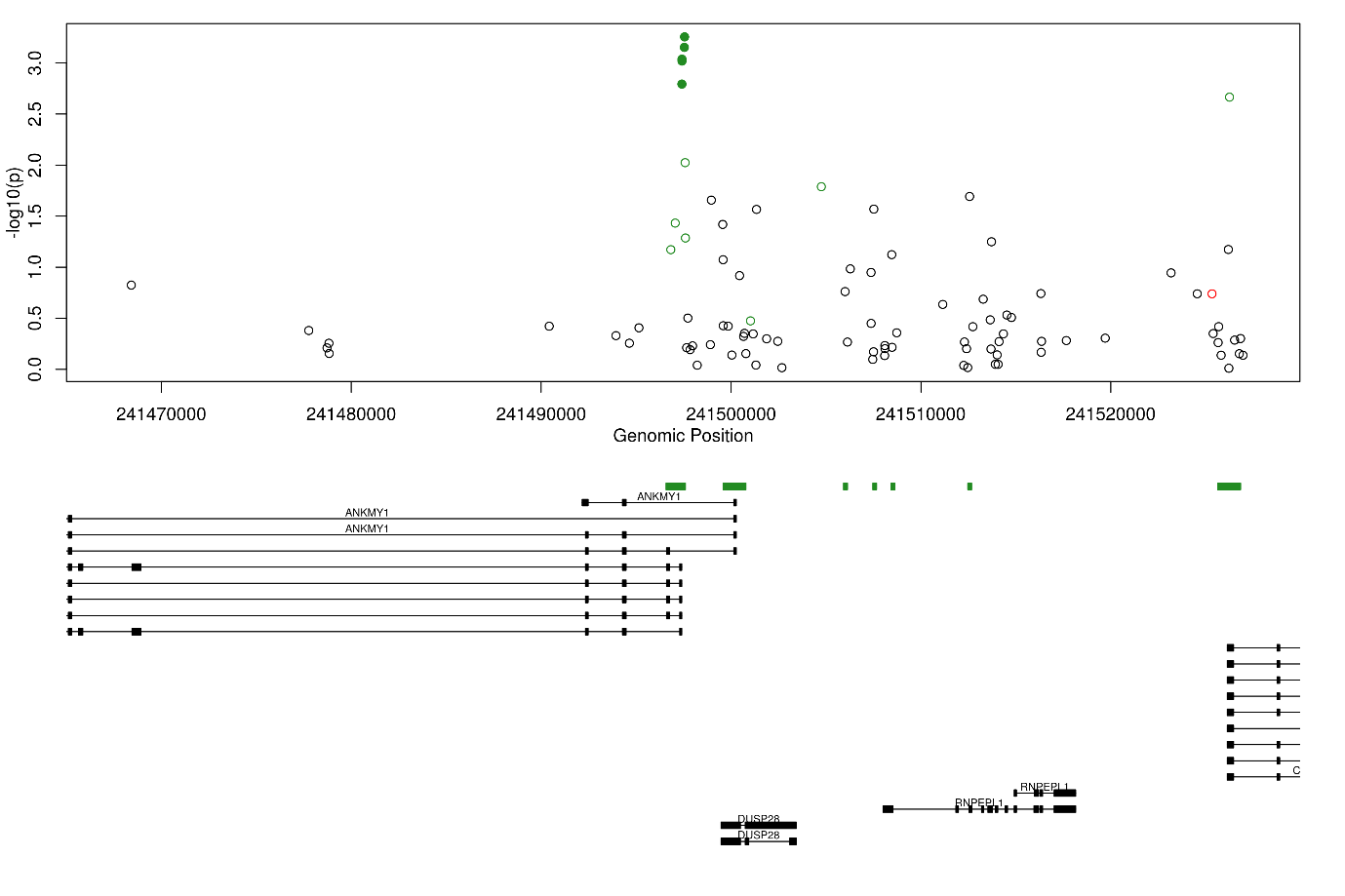


**Supplementary Figure 7: Differentially methylated regions (DMRs) associated with CSF Aβ38 levels**. We identified two DMRs, including (**A**) four probes in the *STRA6* gene (chr15:74495276-74495401) and (**B**) five probes in the *TGFBI* gene (chr5:135364552-135364580). The X-axis shows genomic position, whilst the Y-axis shows -log10(p). Red probes (circles) represent a positive effect size (ES) ≥ 1%, green probes (circles) represent a negative ES ≥ 1% and black probes (circles) represent an ES smaller than 1%. Filled circles denote the probes in the DMR. ES is defined as the % methylation difference across the range of values. gene tracks are shown in black underneath, with CpG islands in green. Full details on the DMRs can be found in Supplementary Table 15D.

**A**


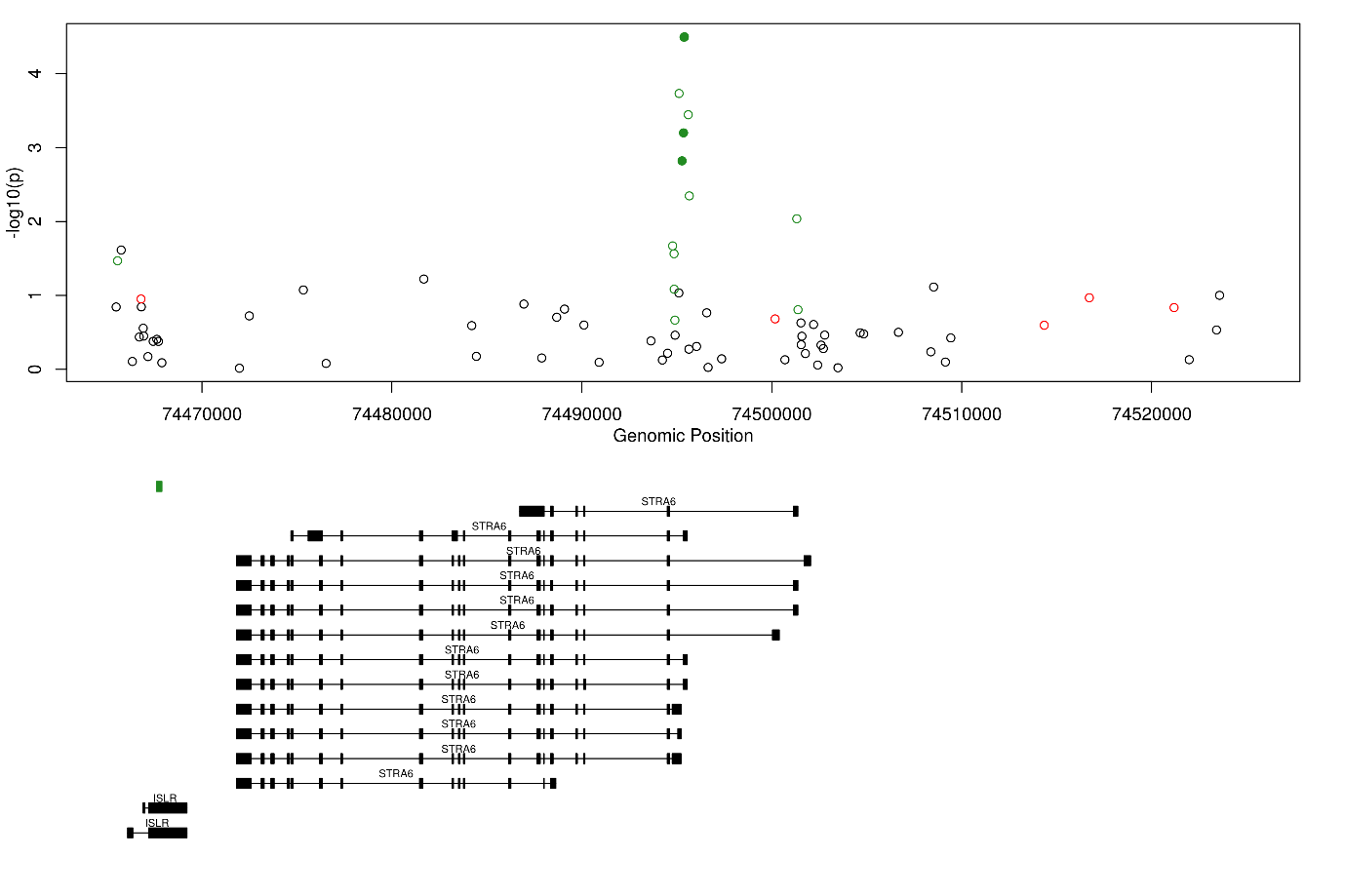


**Supplementary Figure 7 cont.**

**B**


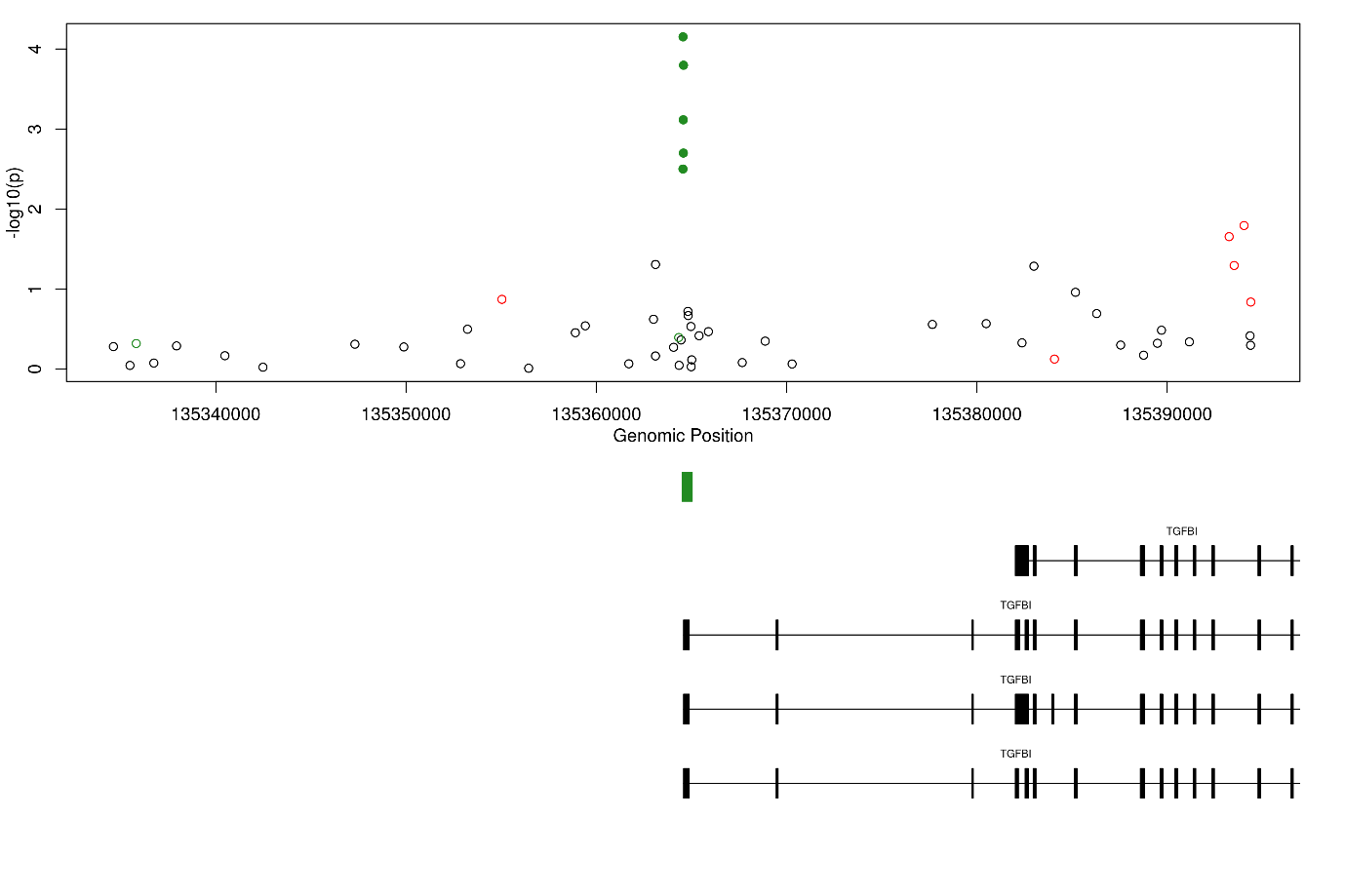


**Supplementary Figure 8: Differentially methylated regions (DMRs) associated with CSF Aβ Z-score**. We identified four DMRs, including (**A**) five probes in the *MX2* gene (chr21:42741698-42741991), (**B**) 15 probes in the *ZFP57* gene (chr6:29648225-29648623), (**C**) six probes in the *FURIN* gene (chr15:91415964-91416118) and (**D**) five probes in the *CD24* gene (chrY:21154667-21154816). The X-axis shows genomic position, whilst the Y-axis shows -log10(p). Red probes (circles) represent a positive effect size (ES) ≥ 1%, green probes (circles) represent a negative ES ≥ 1% and black probes (circles) represent an ES smaller than 1%. Filled circles denote the probes in the DMR. ES is defined as the % methylation difference across the range of values. The gene tracks are shown in black underneath, with CpG islands in green. Full details on the DMRs can be found in Supplementary Table 15E.

**A**


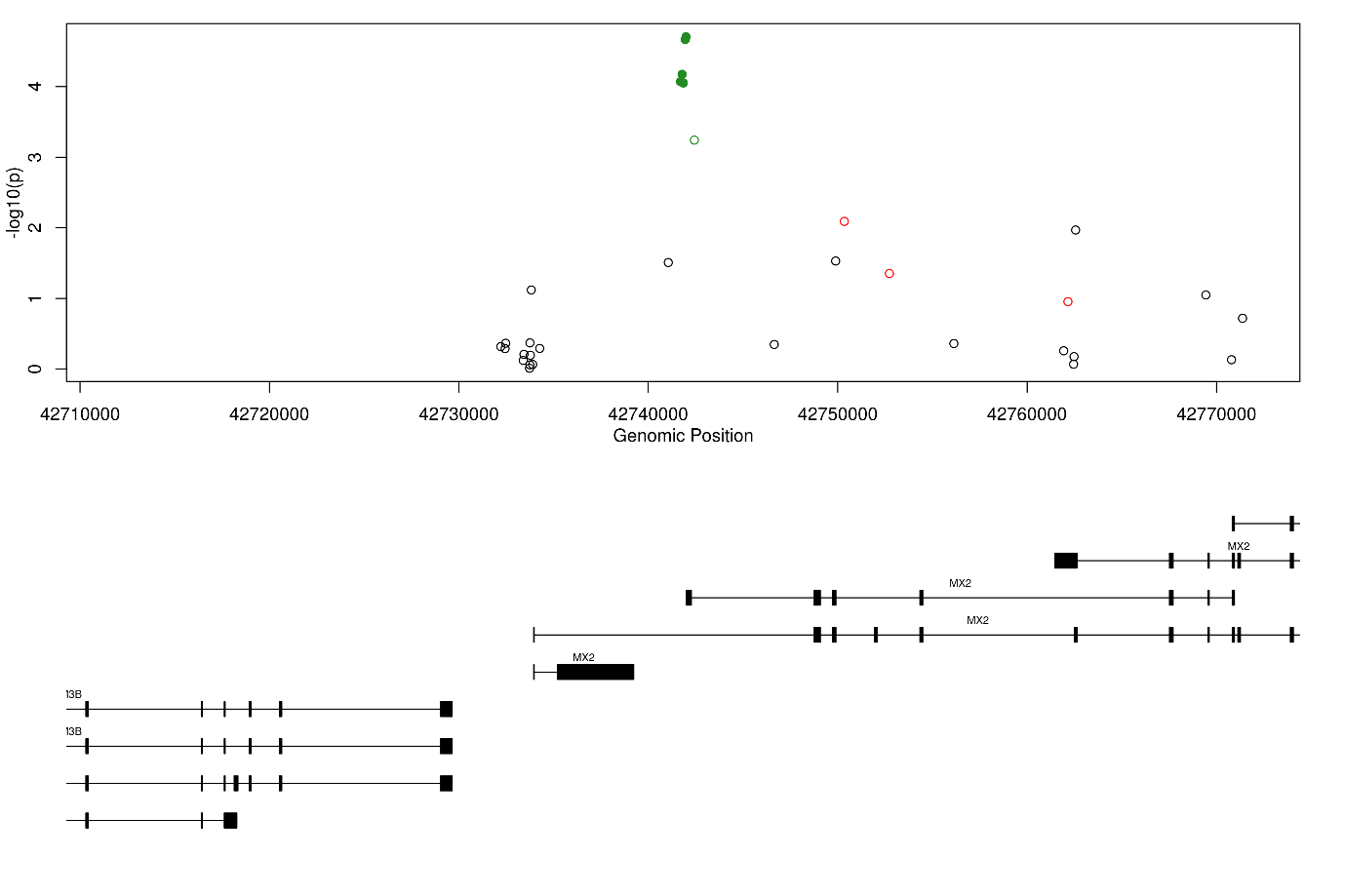


**Supplementary Figure 8 cont.**

**B**


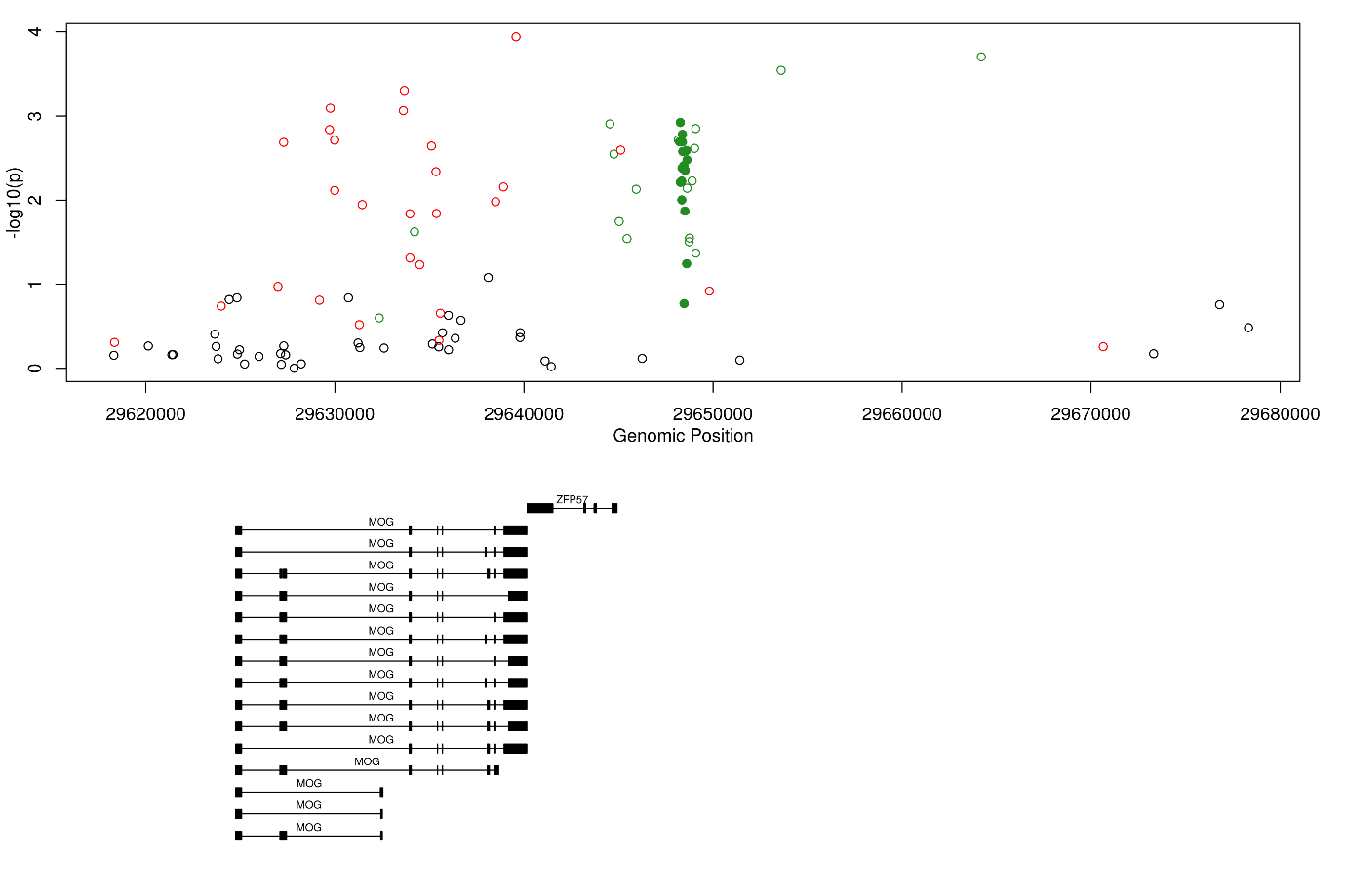


**C**


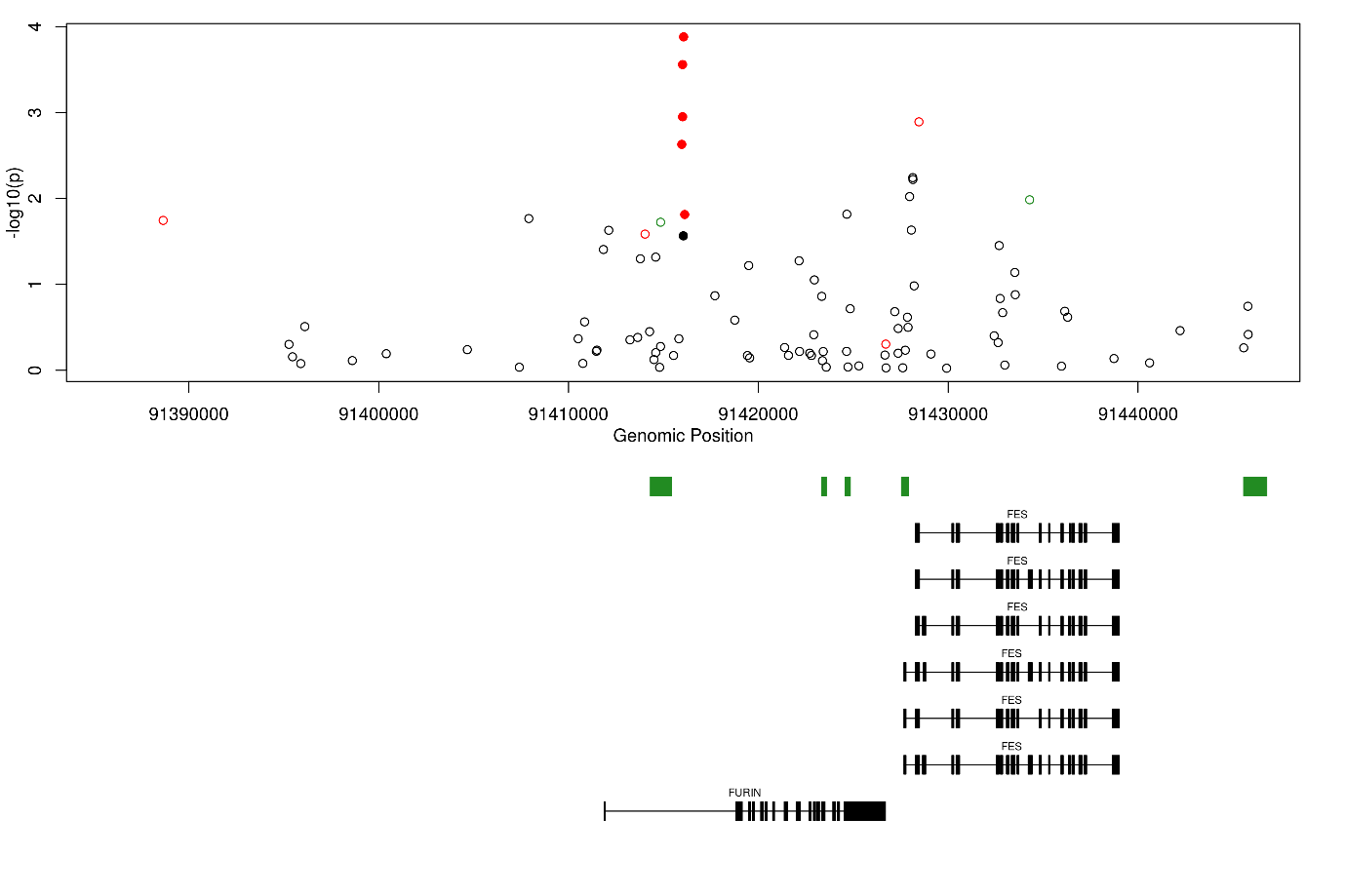


**Supplementary Figure 8 cont.**

**D**


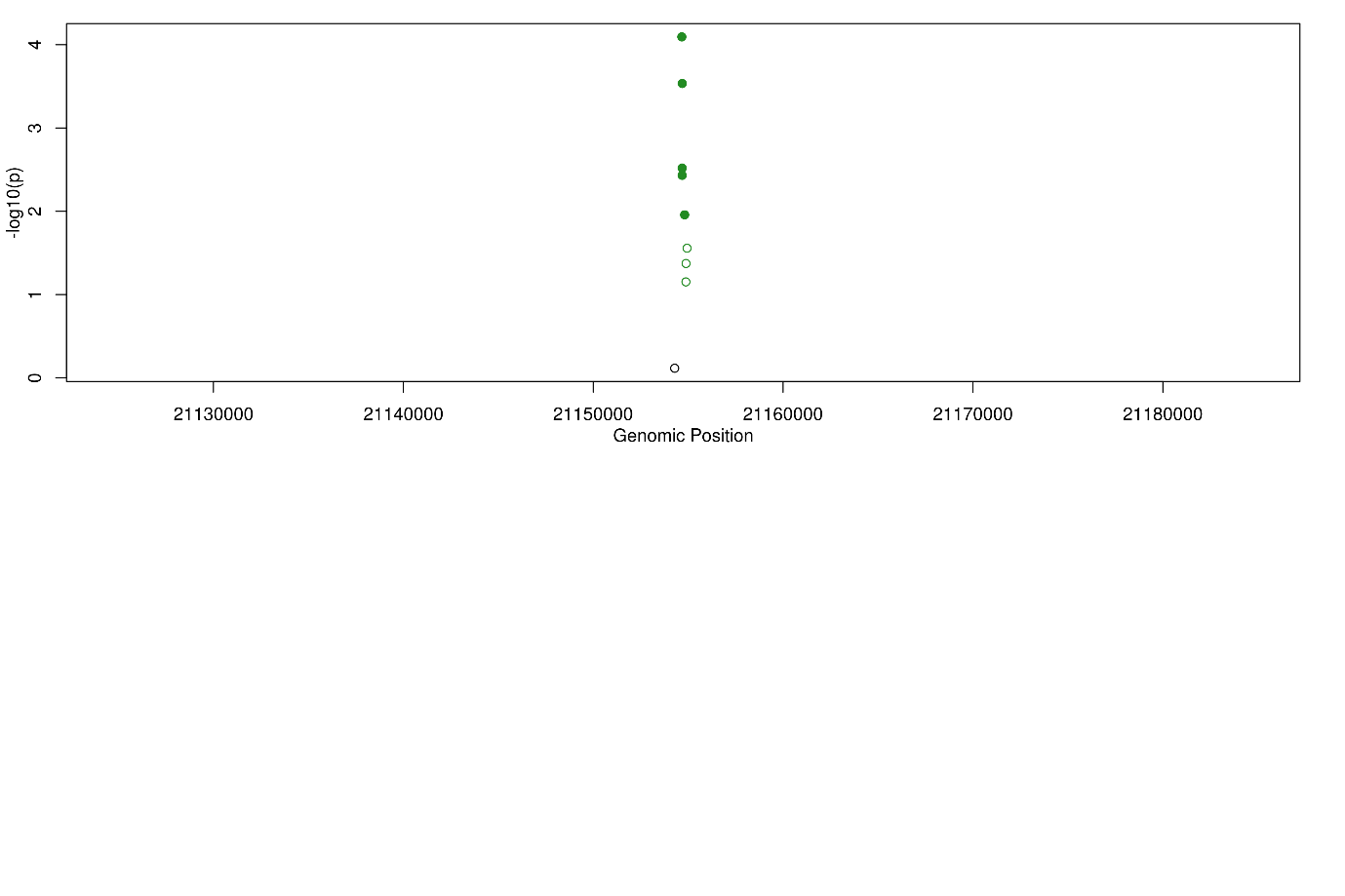


**Supplementary Figure 9: The differentially methylated region (DMR) associated with CSF Aβ42/40 ratio**. We identified one DMR spanning 12 probes in the *ZFP57* gene (chr6:29648225-29648525). The X-axis shows genomic position, whilst the Y-axis shows -log10(p). Red probes (circles) represent a positive effect size (ES) ≥ 1%, green probes (circles) represent a negative ES ≥ 1% and black probes (circles) represent an ES smaller than 1%. Filled circles denote the probes in the DMR. ES is defined as the % methylation difference across the range of values. The gene tracks are shown in black underneath, with CpG islands in green. Full details on the DMR can be found in Supplementary Table 15F.


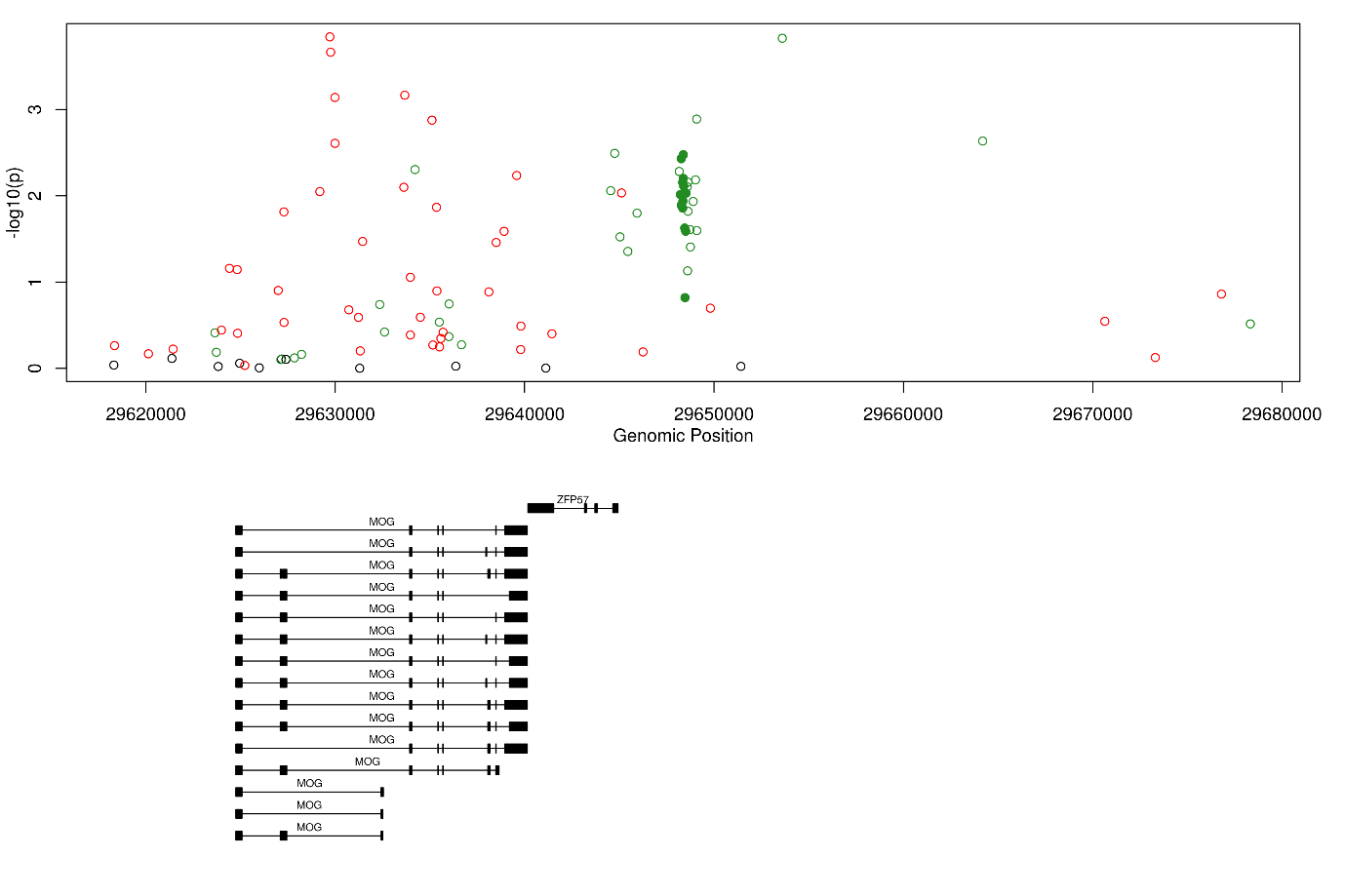


**Supplementary Figure 10: The differentially methylated region (DMR) associated with amyloid status**. We identified one DMR spanning five probes in the *MX2* gene (chr21:42741698-42741991). The X-axis shows genomic position, whilst the Y-axis shows -log10(p). Red probes (circles) represent a positive effect size (ES) ≥ 1%, green probes (circles) represent a negative ES ≥ 1% and black probes (circles) represent an ES smaller than 1%. Filled circles denote the probes in the DMR. ES is defined as the % methylation difference across the range of values. The gene tracks are shown in black underneath, with CpG islands in green. Full details on the DMR can be found in Supplementary Table 15G.


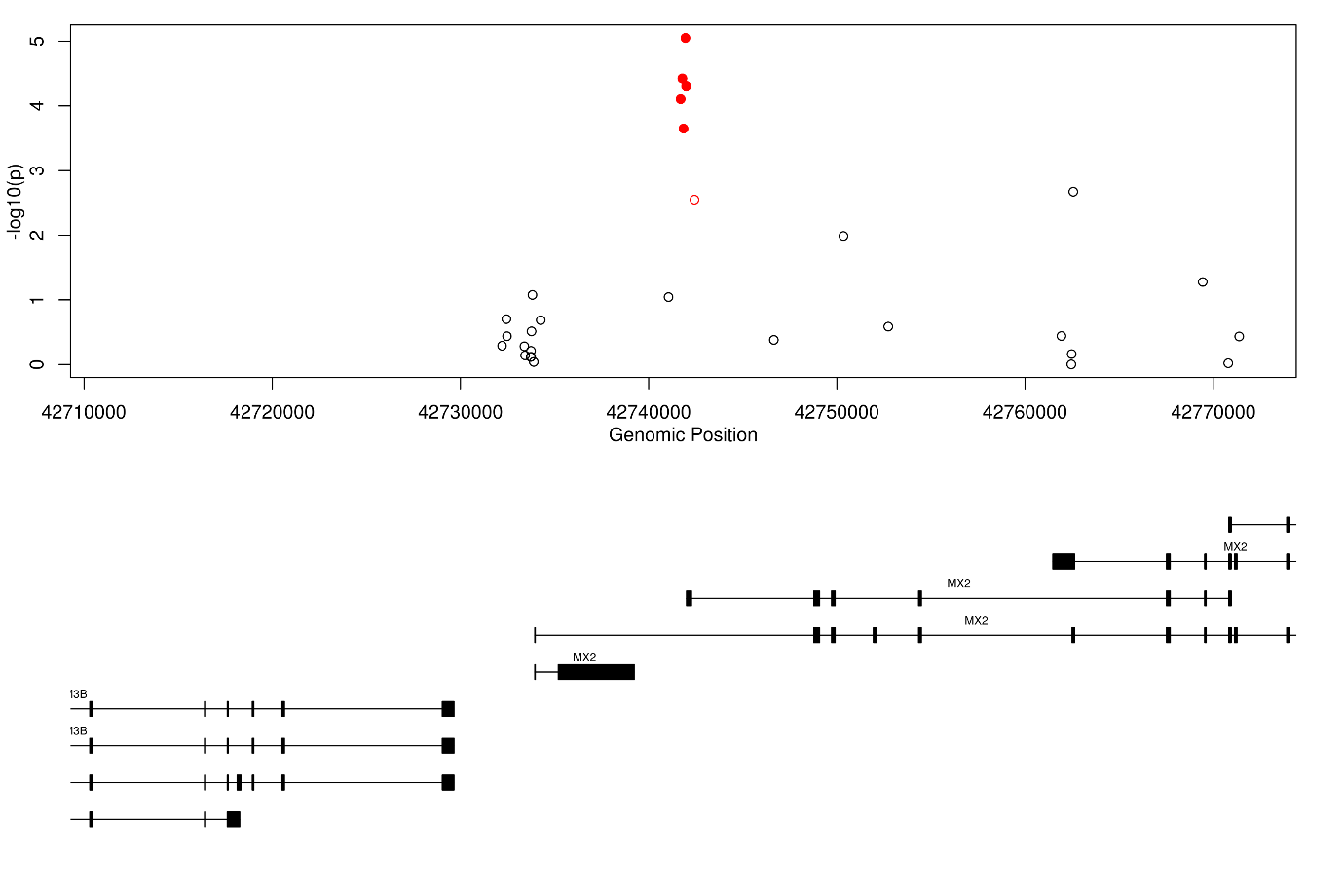


**Supplementary Figure 11: Differentially methylated regions (DMRs) associated with CSF YKL-40 levels**. We identified three DMRs, including eight probes in the *CHI3L1* gene (chr1:203155737-203156784) (shown in Figure 1B), (**A**) seven probes in the *HS3ST3B1* gene (chr17:14206774-14207036) and (**B**) four probes in the *CYP26C1* gene (chr10:94820892-94820988). The X-axis shows genomic position, whilst the Y-axis shows -log10(p). Red probes (circles) represent a positive effect size (ES) ≥ 1%, green probes (circles) represent a negative ES ≥ 1% and black probes (circles) represent an ES smaller than 1%. Filled circles denote the probes in the DMR. ES is defined as the % methylation difference across the range of values. The gene tracks are shown in black underneath, with CpG islands in green. Full details on the DMRs can be found in Supplementary Table 19A.

**A**


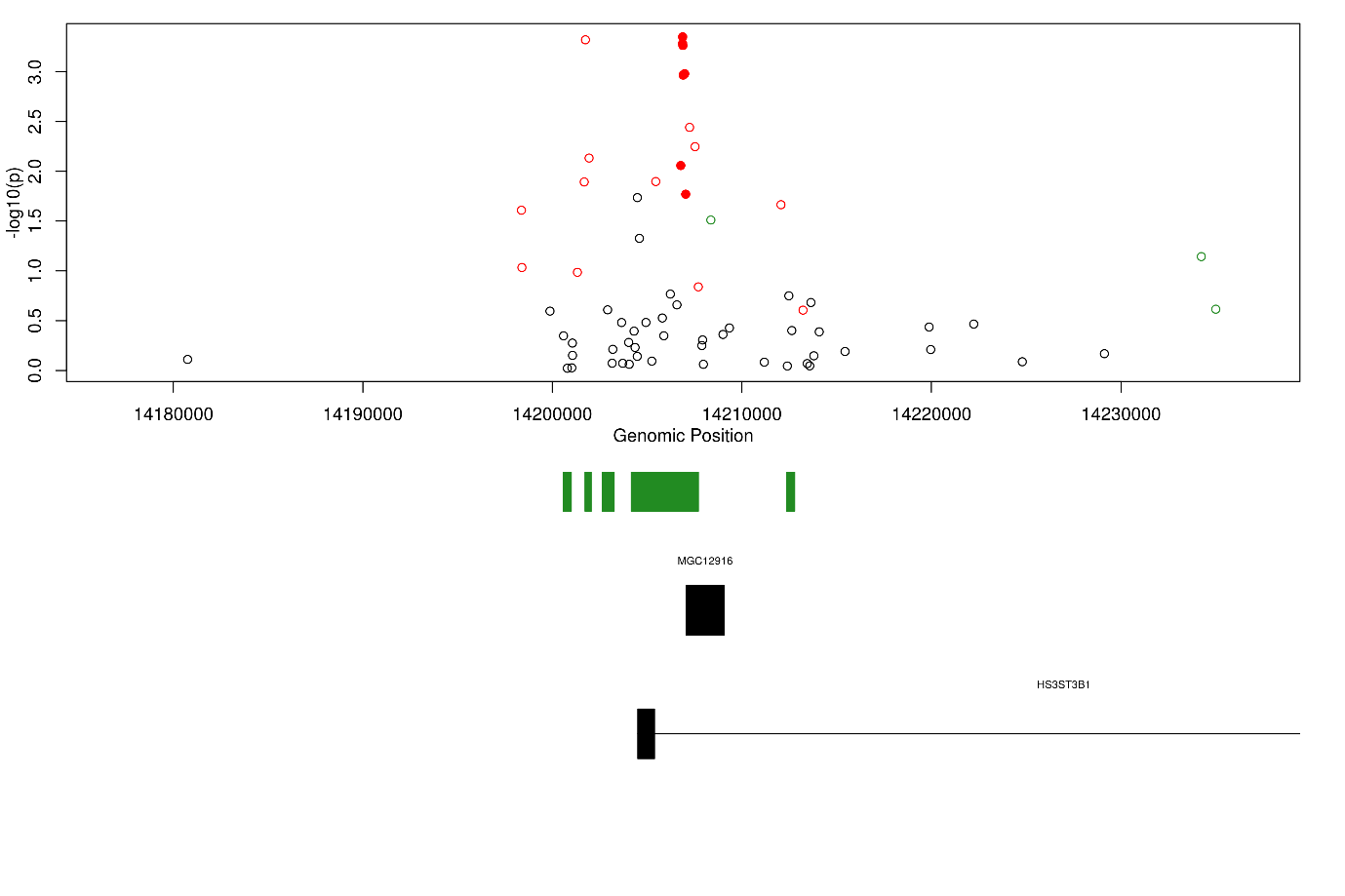


**Supplementary Figure 11 cont.**

**B**


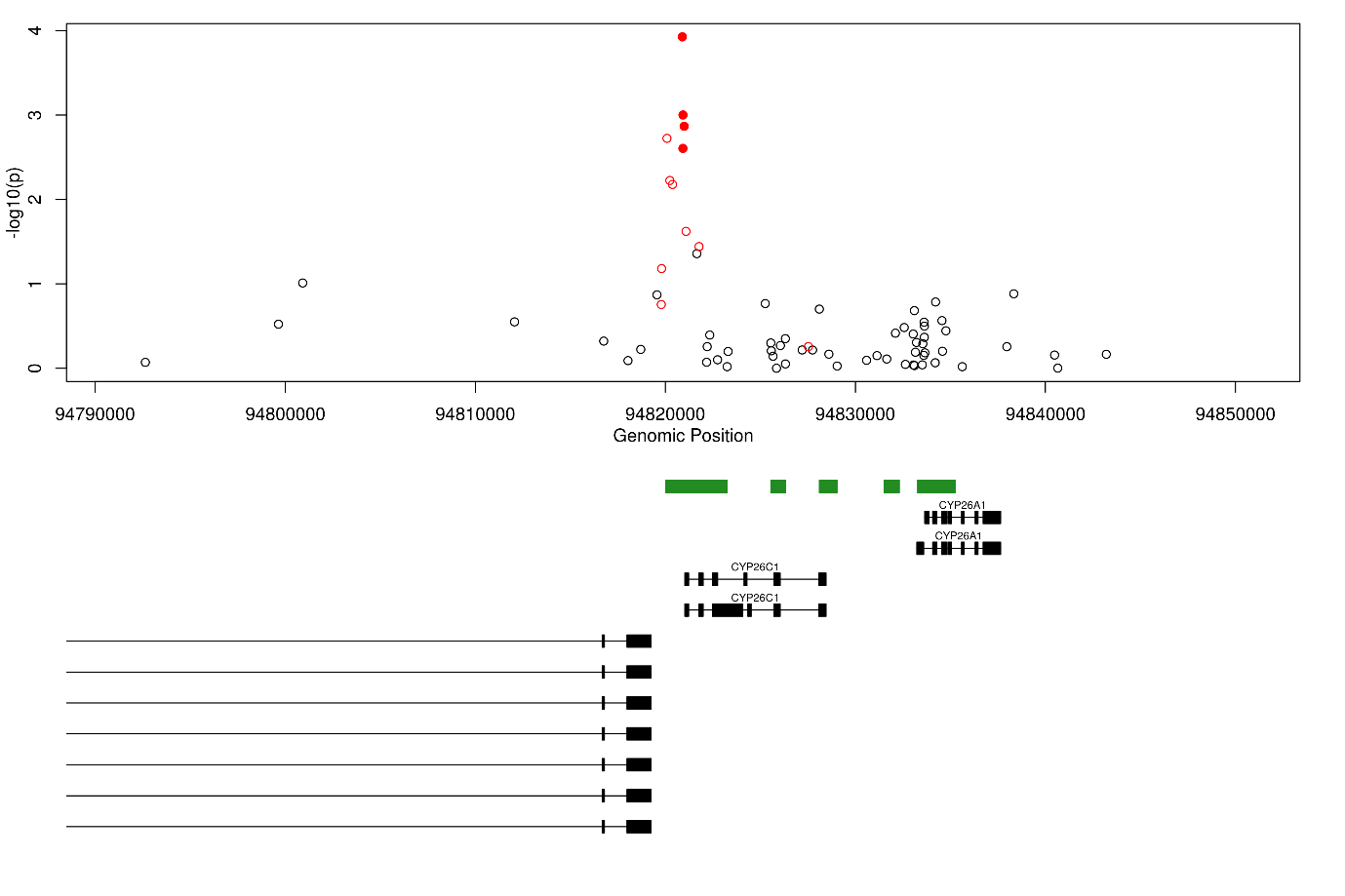


**Supplementary Figure 12: Differentially methylated regions (DMRs) associated with CSF NFL**. We identified seven DMRs, including (**A**) six probes in the *TEX12* gene (chr11:112037969-112038173), (**B**) eight probes in the *SORD* gene (chr15:45315202-45315438), (**C**) ten probes in the *S100A13* gene (chr1:153599573-153599831), (**D**) three probes in the *CCDC71L* gene (chr7:106301534-106301660), (**E**) five probes in the *NAALAD2* gene (chr11:89867809-89867976), (**F**) five probes in the *STK16* gene (chr2:220110237-220110385), (**G**) three probes in the *CNTN3* gene (chr3:74316889-74316993) and (**H**) four probes in the *PRDM9* gene (chr5:23507562-23507617). The X-axis shows genomic position, whilst the Y-axis shows -log10(p). Red probes represent a positive effect size (ES) ≥ 1%, green probes represent a negative ES ≥ 1% across the range of the analysis. Filled circles denote the probes in the DMR. ES is defined as the % methylation difference across the range of values. The gene tracks are shown in black underneath, with CpG islands in green. Full details on the DMRs can be found in Supplementary Table 19B.

**A**


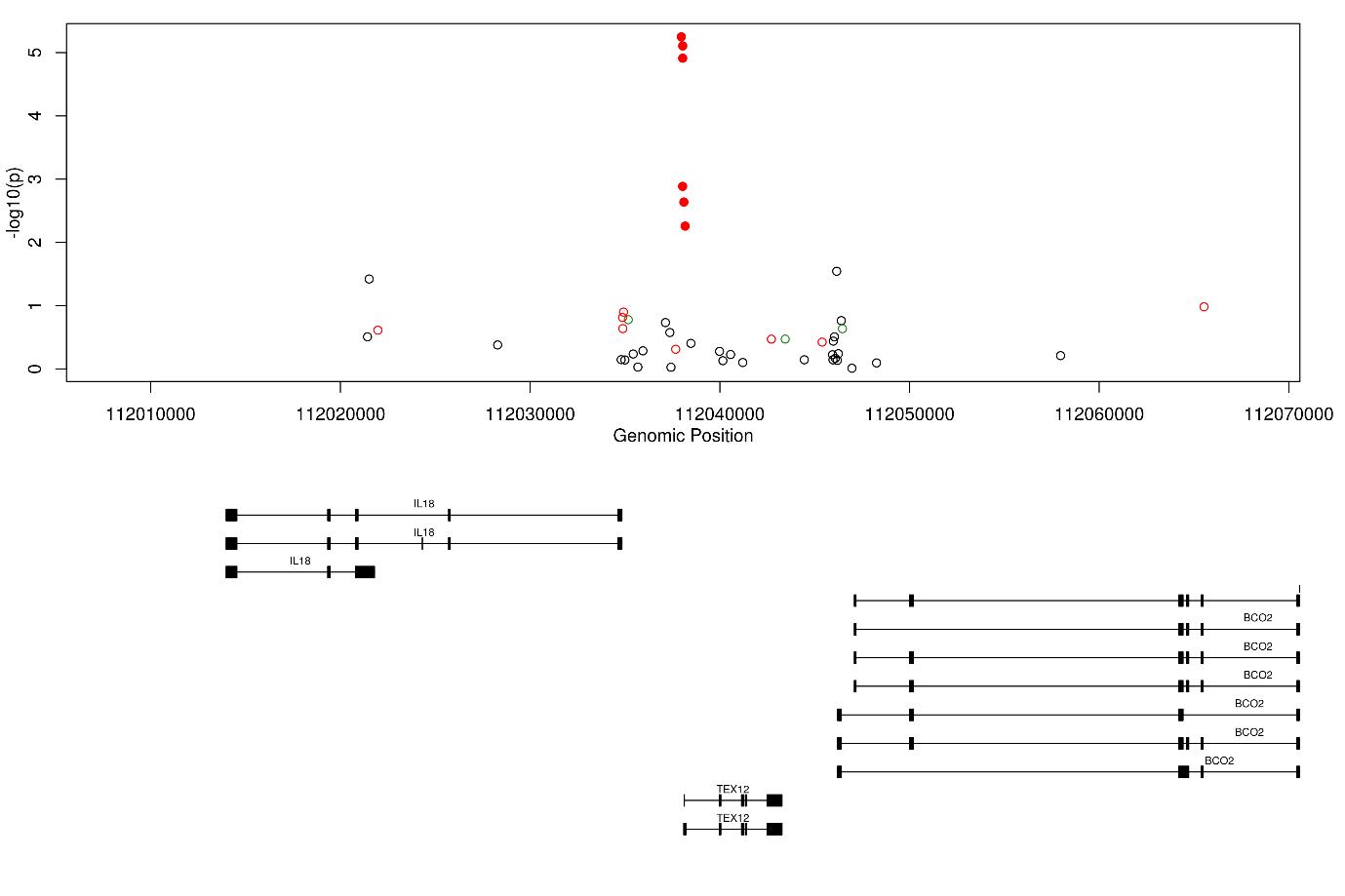


**Supplementary Figure 12 cont.**

**B**


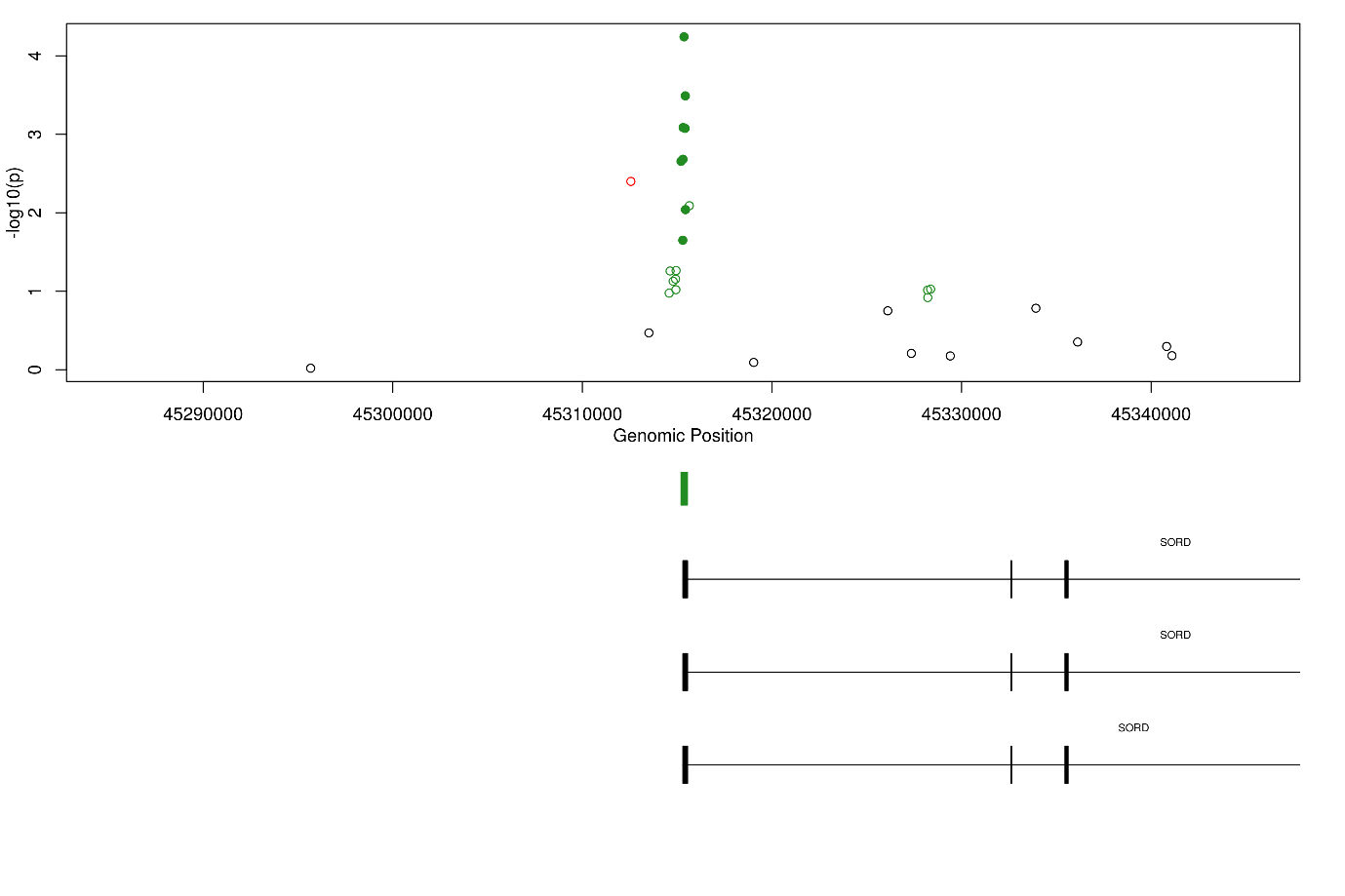


**C**


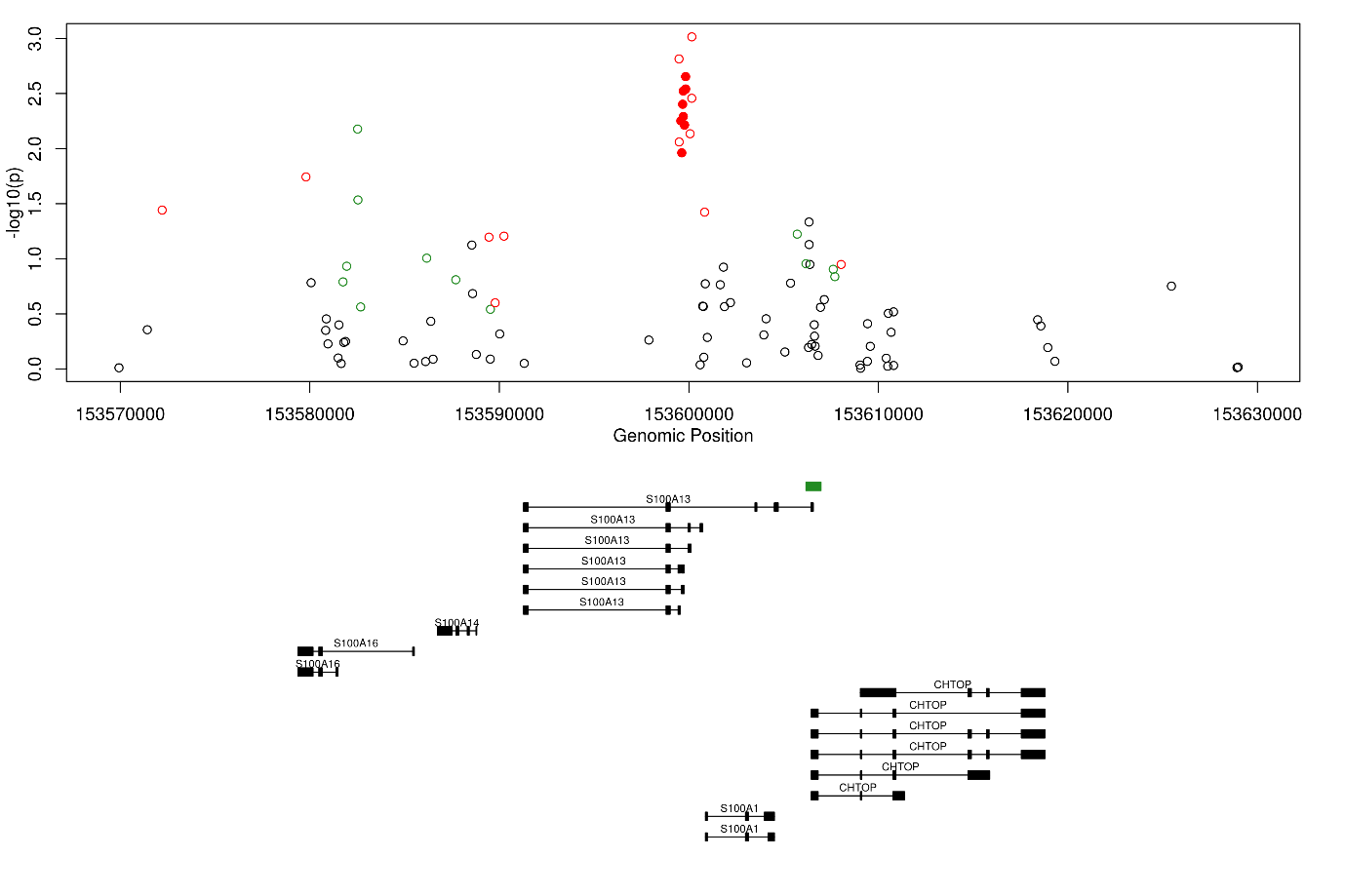


**Supplementary Figure 12 cont.**

**D**


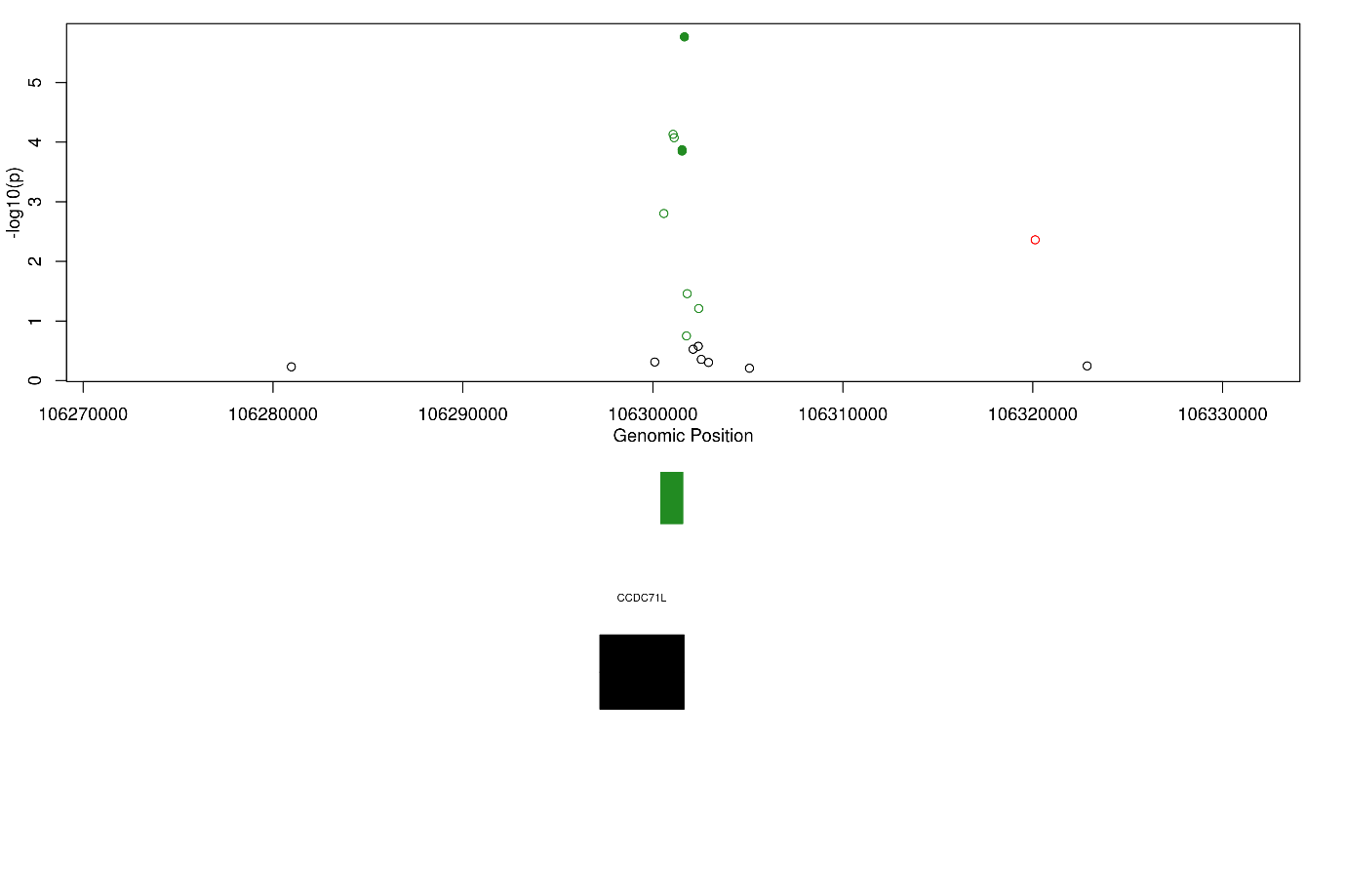


**E**

**
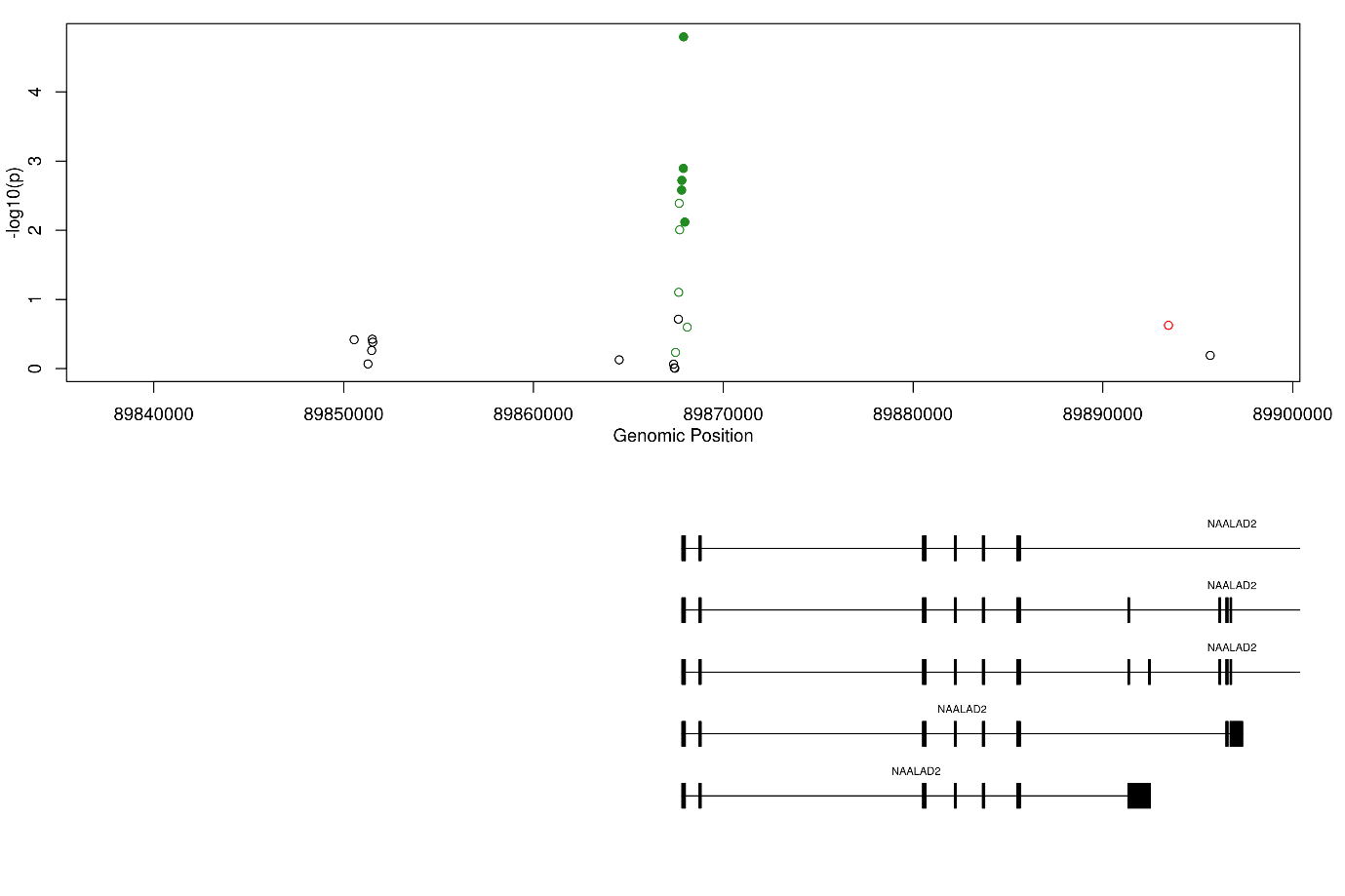
**

**Supplementary Figure 12 cont.**

**F**


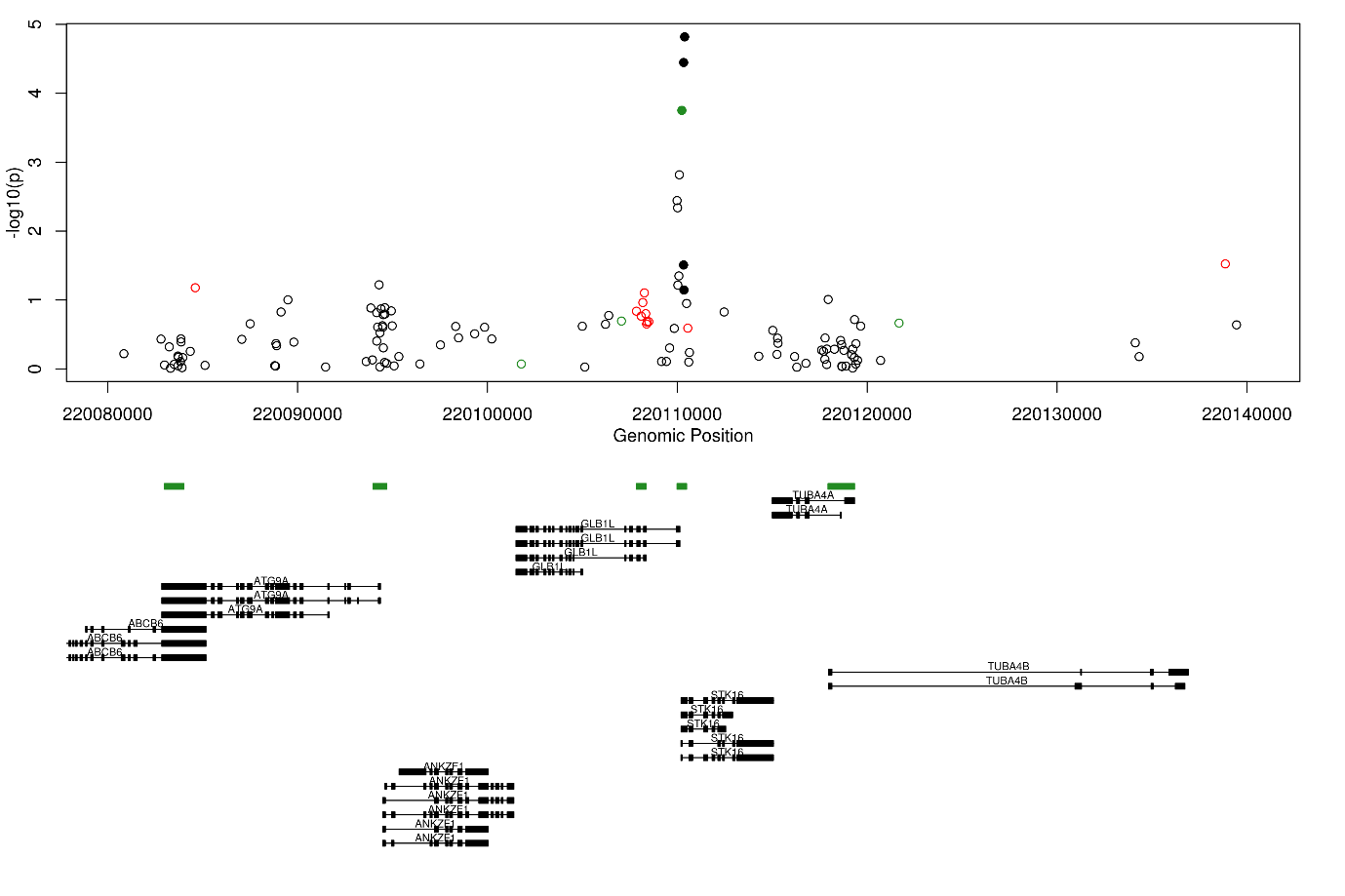


**G**


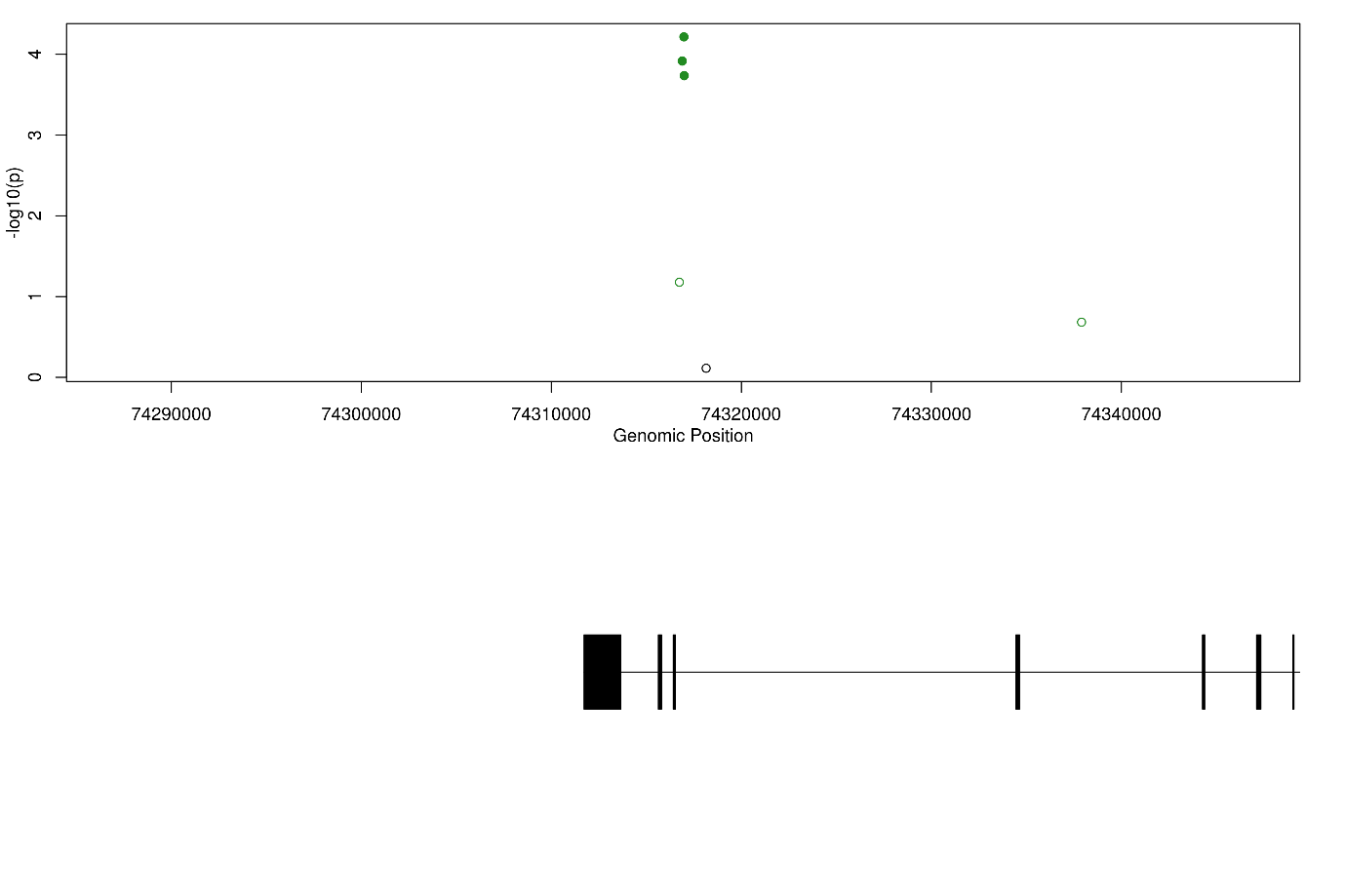


**Supplementary Figure 12 cont.**

**H**


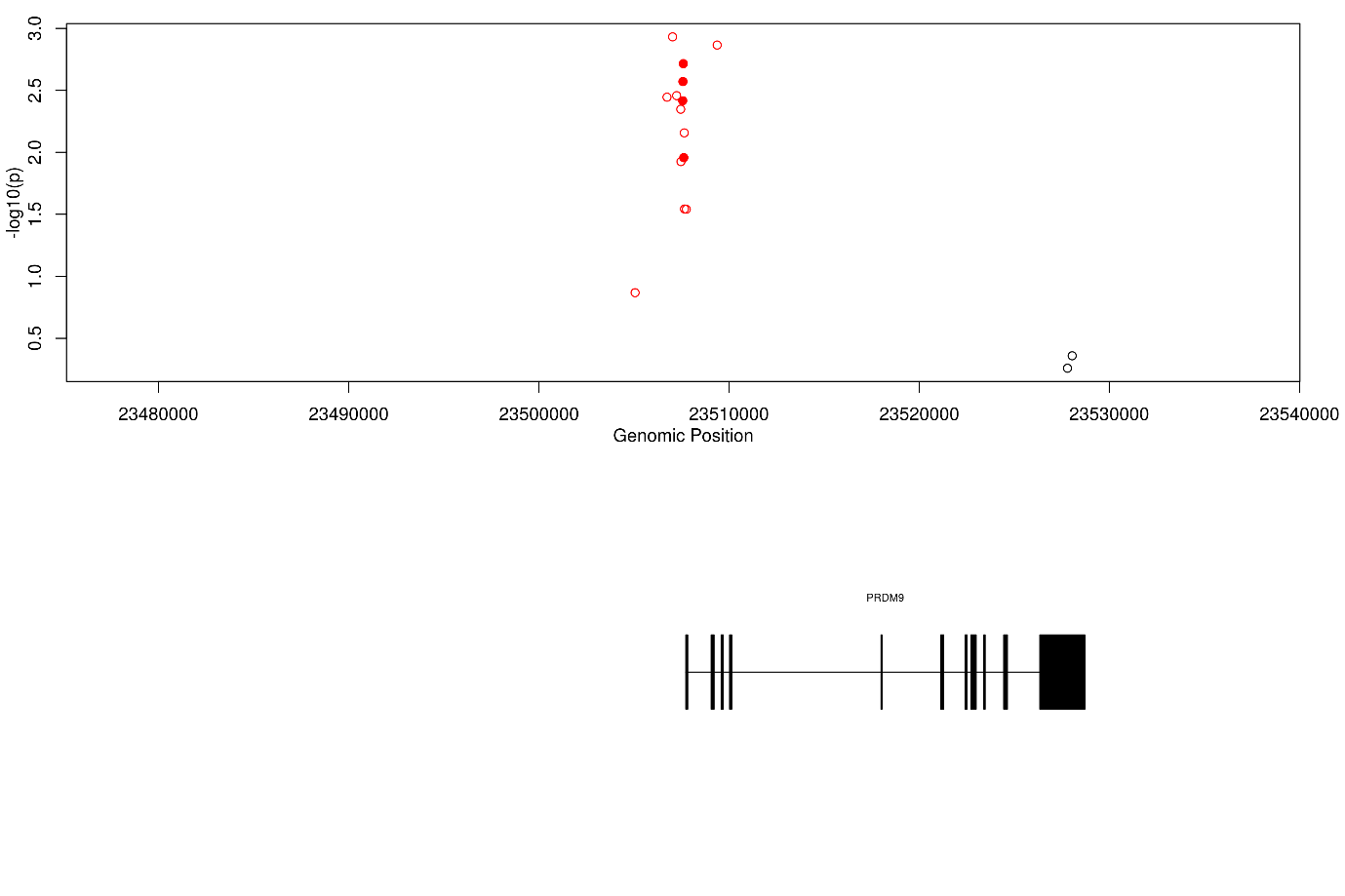


**Supplementary Figure 13: Differentially methylated regions (DMRs) associated with CSF neurogranin**. We identified four DMRs, including (**A**) six probes upstream of the *AVP* gene (chr20:3065473-3065698), (**B**) seven probes in the *SORD* gene (chr15:45315297-45315438), (**C**) four probes in the *STRA6* gene (chr15:74495276-74495401) and (**D**) three probes in the *FAR2* gene (chr12:29302016-29302179). The X-axis shows genomic position, whilst the Y-axis shows -log10(p). Red probes represent a positive effect size (ES) ≥ 1%, green probes represent a negative ES ≥ 1%. Filled circles denote the probes in the DMR. ES is defined as the % methylation difference across the range of values. The gene tracks are shown in black underneath, with CpG islands in green. Full details on the DMRs can be found in Supplementary Table 19C.

**A**


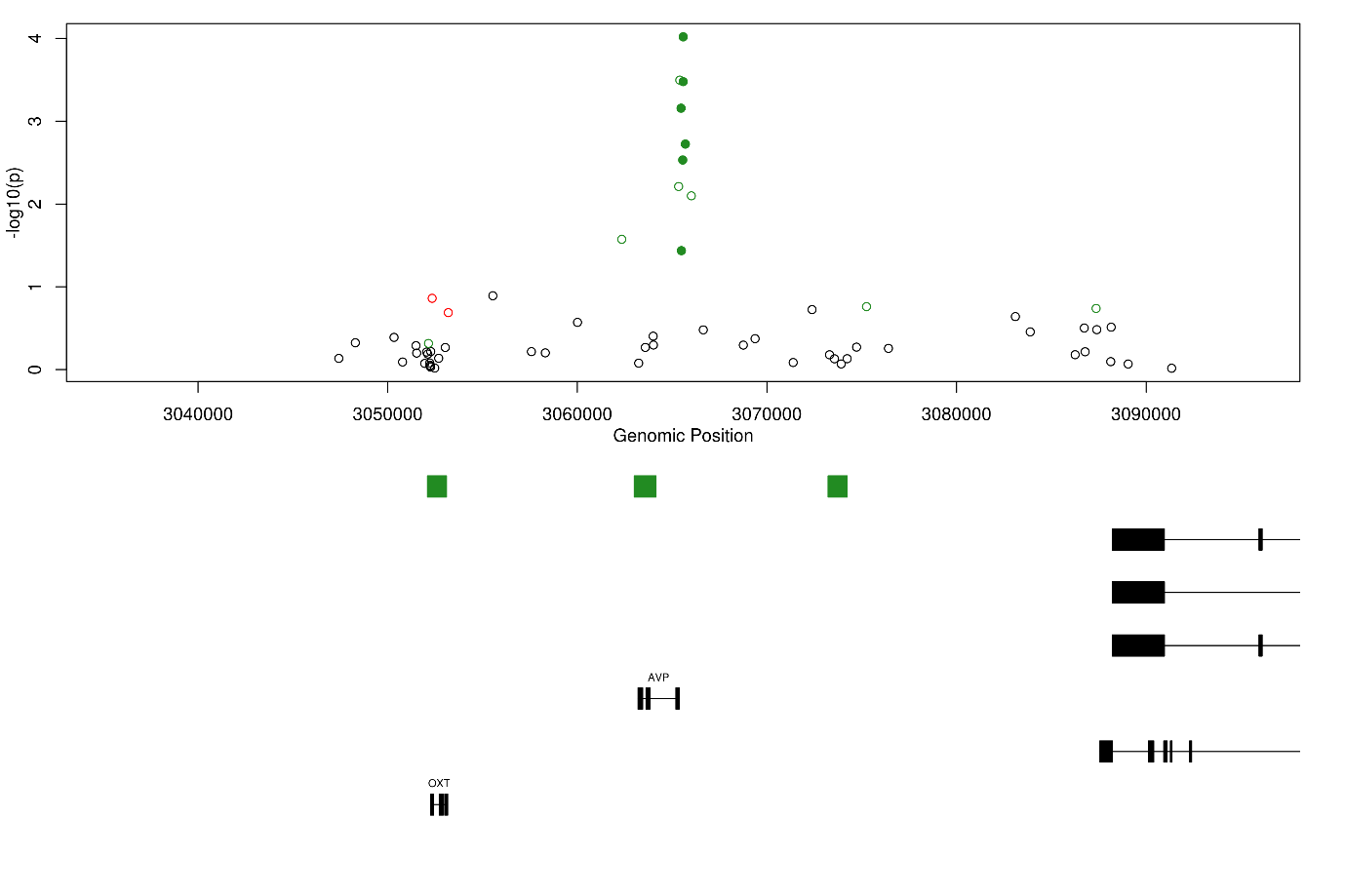


**Supplementary Figure 13 cont.**

**B**


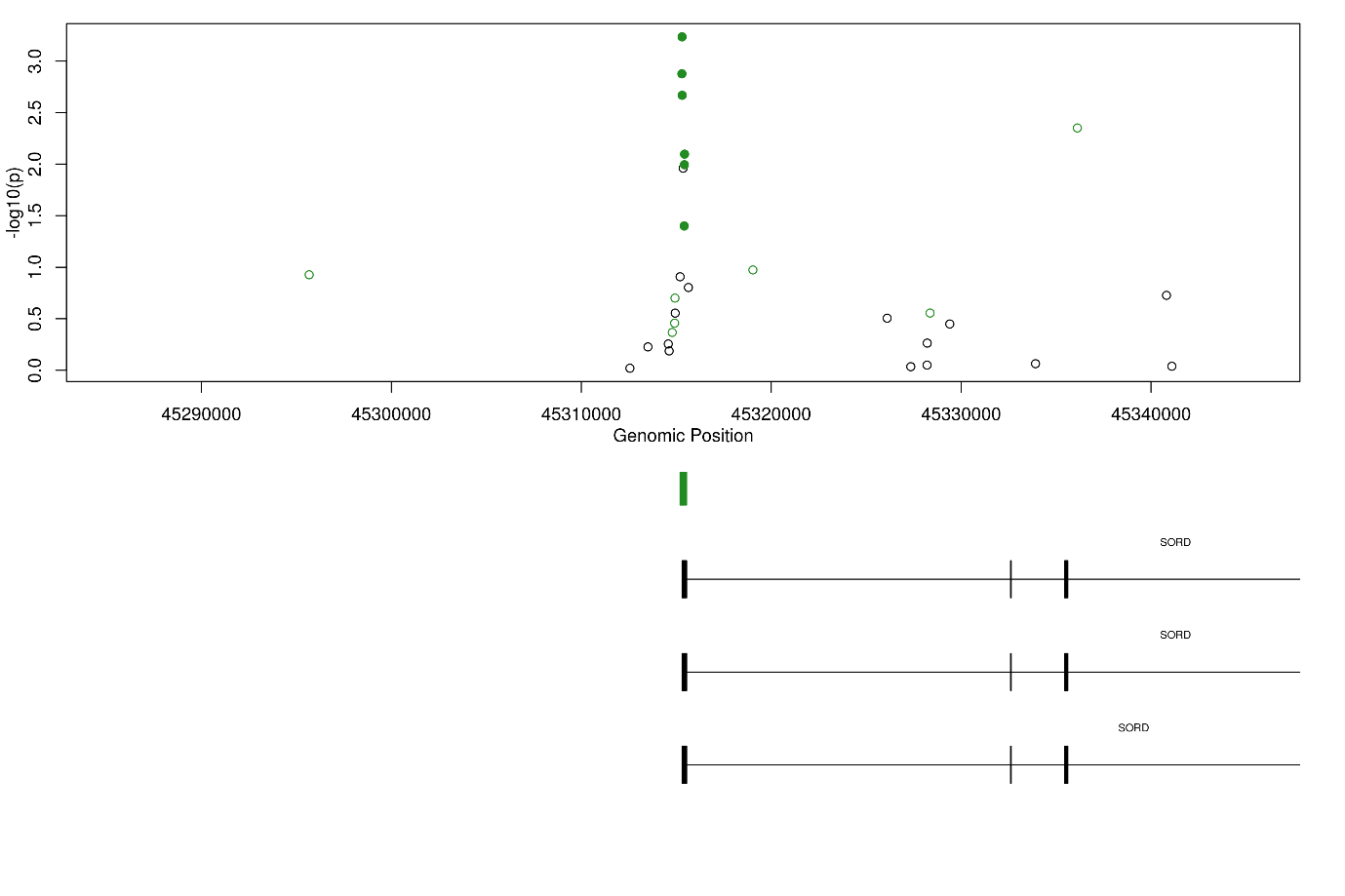


**C**

**
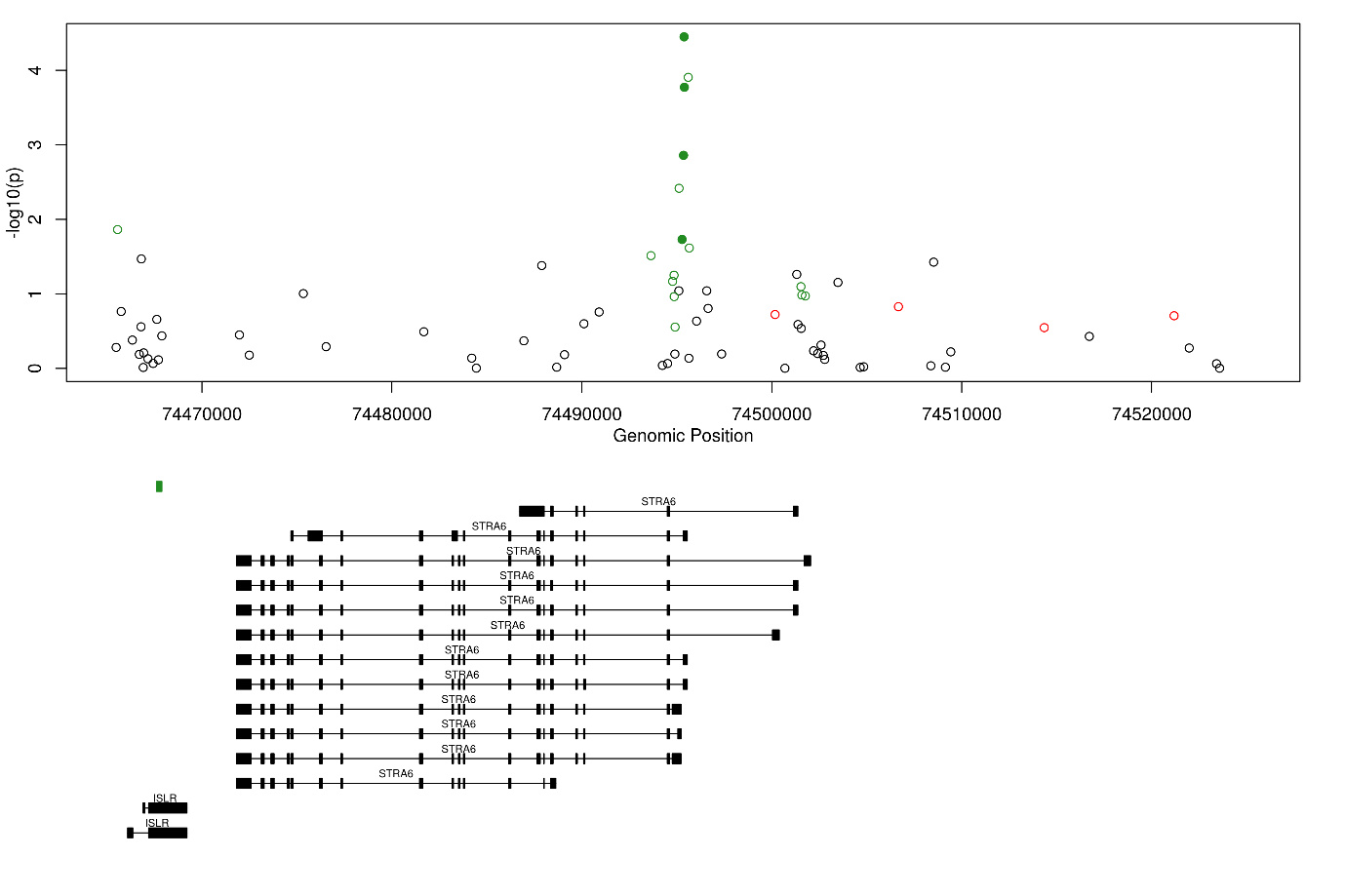
**

**Supplementary Figure 13 cont.**

**D**


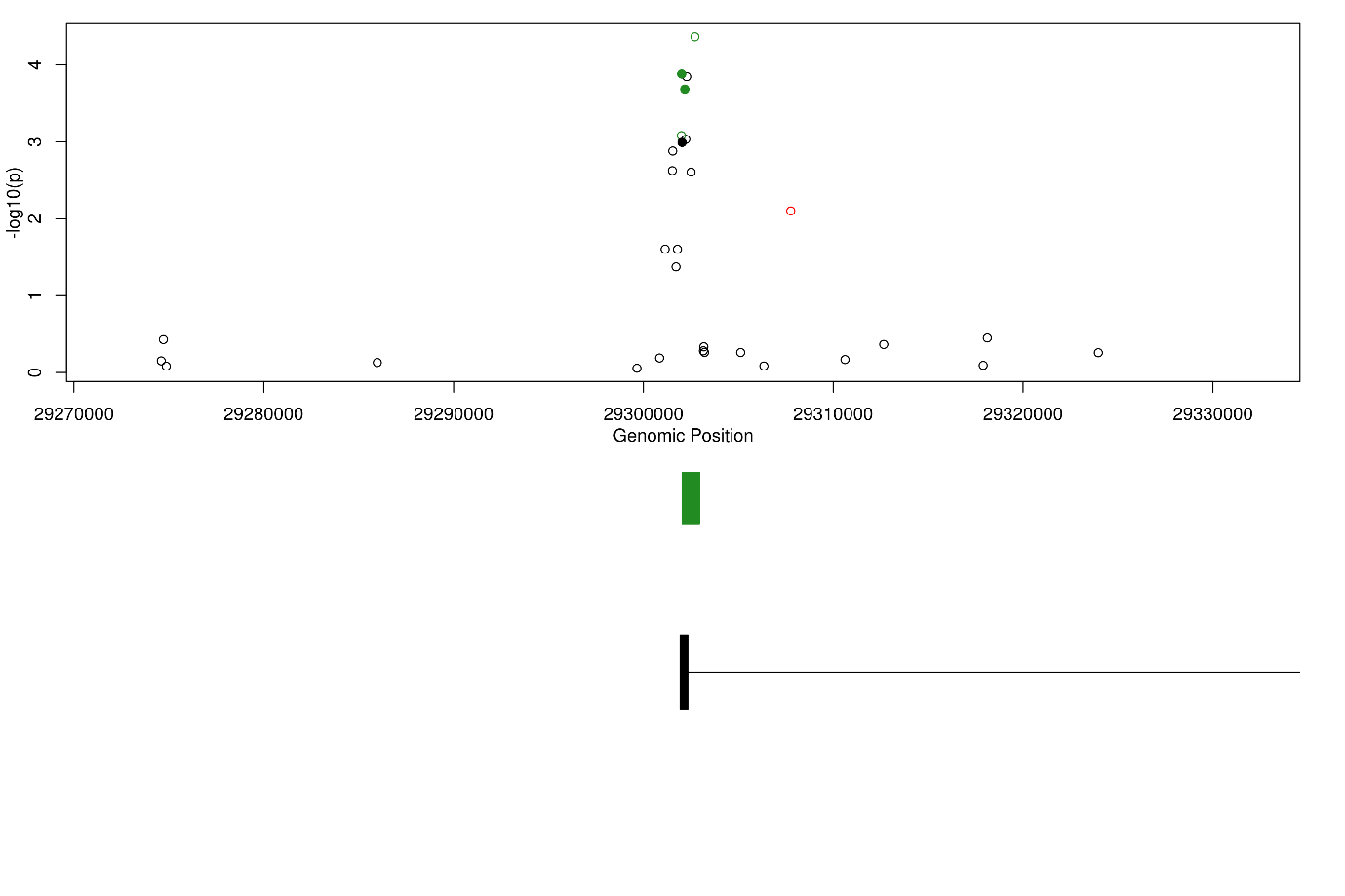


**Supplementary Figure 14: Heatmaps highlighting correlations between module eigengenes and various tau, amyloid and other CSF AD-relevant biomarkers**. Shown is the Pearson’s correlation coefficient (*r*) (upper value) and *P*-value (lower value) for the correlation between each of the 52 modules and disease status (diagnosis), four tau measures (p-tau assay Z-score, abnormal p-tau, t-tau assay z-score, abnormal t-tau), eight amyloid measures (abnormal Aβ42, Aβ42, Aβ40, Aβ38, Aβ Z-score, Aβ42/40 ratio, Aβ42/40 ratio dichotomized, amyloid status) and three other biomarkers of neuroinflammation (YKL-40), neurodegeneration (NFL) and synaptic dysfunction (neurogranin). The heatmap is divided to show 26 modules in (**A**) and 26 modules in (**B**). Modules were taken forward for downstream analyses if they passed the multiple testing threshold of *P* < 9.62 x 10^-4^, which accounts for the 52 modules tested.

**A**


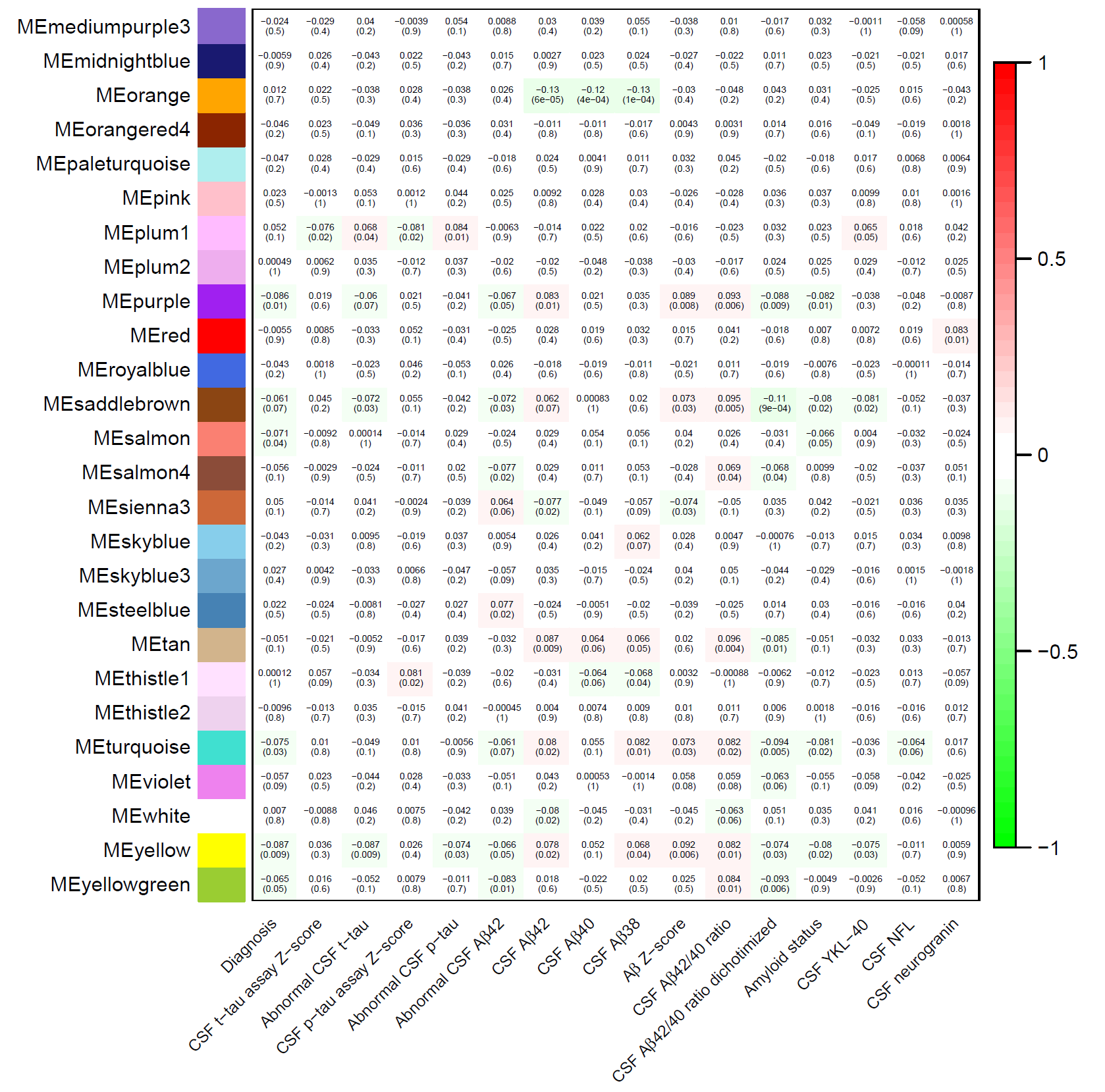


**Supplementary Figure 14 cont.**

**B**


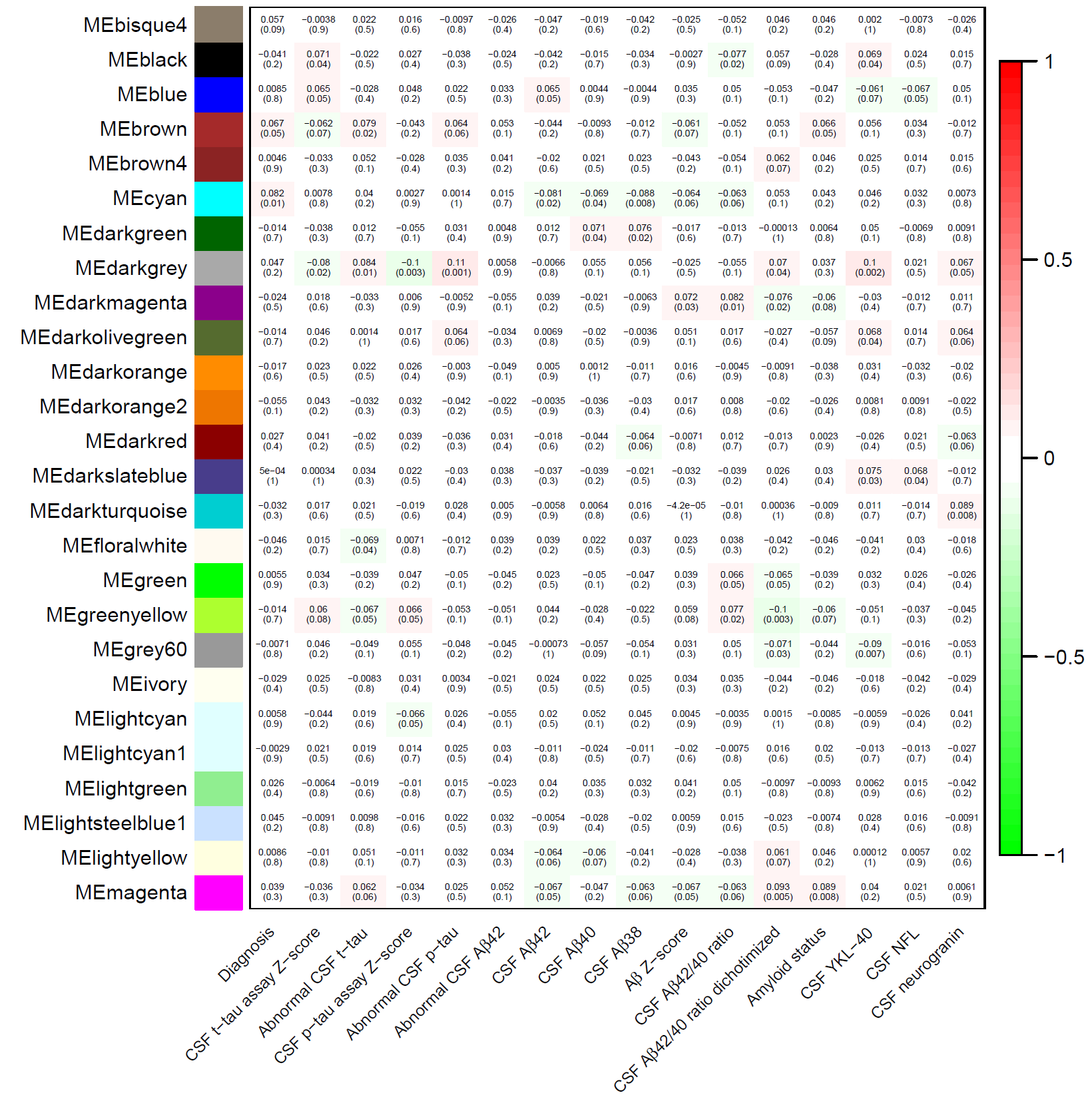

Supplement: Supplementary file 1 — Supporting Information [file ALZ-20-6722-s001.docx]
